# Computational analysis of TP53 mutational landscape unveils key prognostic signatures and distinct pathobiological pathways in head and neck squamous cell cancer

**DOI:** 10.1038/s41416-020-0984-6

**Published:** 2020-07-20

**Authors:** Vito Carlo Alberto Caponio, Giuseppe Troiano, Iolanda Adipietro, Khrystyna Zhurakivska, Claudia Arena, Domenica Mangieri, Marco Mascitti, Nicola Cirillo, Lorenzo Lo Muzio

**Affiliations:** 1 Department of Clinical and Experimental Medicine, University of Foggia, Foggia, Italy; 2 Department of Medical and Surgical Sciences, Biomedical Unit ‘E. Altomare’, University of Foggia, Foggia, Italy; 3 Department of Clinical Specialistic and Dental Sciences, Marche Polytechnic University, Ancona, Italy; 4 Melbourne Dental School, The University of Melbourne, Melbourne, VIC Australia

**Keywords:** Gene ontology, Head and neck cancer, Mutagenesis, Prognostic markers

## Abstract

**Background:**

Mutations of the tumour-suppressor gene TP53 are the most frequent somatic genomic alterations in head and neck squamous cell carcinoma (HNSCC). However, it is not yet clear whether specific TP53 mutations bear distinct clinical and pathophysiological significance in different HNSCC subgroups.

**Methods:**

A systematic bioinformatics appraisal of TP53 mutations was performed on 415 HNSCC cases available on The Cancer Genome Atlas (TCGA). The following features were analysed and correlated with known clinicopathological variables: mutational profile of TP53, location (within secondary structure and predicted domains of p53 protein) and well-known hotspot mutations. Interactome–genome–transcriptome network analysis highlighted different gene networks. An algorithm was generated to develop a new prognostic classification system based on patients’ overall survival.

**Results:**

TP53 mutations in HNSCCs exhibited distinct differences in different anatomical sites. The mutational profile of TP53 was an independent prognostic factor in HNSCC. High risk of death mutations, identified by our novel classification algorithm, was an independent prognostic factor in TCGA HNSCC database. Finally, network analysis suggested that distinct p53 molecular pathways exist in a site- and mutation-specific manner.

**Conclusions:**

The mutational profile of TP53 may serve as an independent prognostic factor in HNSCC patients, and is associated with distinctive site-specific biological networks.

## Background

Mutations of the tumour-suppressor gene TP53 are among the most common genomic alterations occurring in malignancy, including head and neck squamous cell carcinoma (HNSCC). HNSCC is the sixth most common cancer with an annual incidence of ~600,000 new cases worldwide. HNSCC frequently presents as a locally advanced disease,^1^ and is characterised by biologically and molecularly diverse groups of tumours. More than 90% of HNSCCs involve the mucosal surfaces of the oral cavity, oropharynx and larynx.^2^ HNSCC frequently presents in individuals with a history of tobacco and alcohol consumption.^3,4^ Both tobacco and alcohol cause DNA damage with an increased likelihood of generating mutations in cancer-related genes.^5^ Consistently, several studies have shown that such carcinogens contribute to the mutational profile of TP53.^6–9^

TP53 encodes p53, a protein that regulates the expression of a vast array of target genes. In early studies, p53 was considered an oncogene because of the lower expression level in normal cells compared with the cancerous one.^10^ In 1989, Levine et al. showed that wild-type p53 works as an oncosuppressor.^11^ Because of its important role in cancer biology, p53 has been defined “the guardian of the genome”. The protein is involved in different cellular functions, such as apoptosis, differentiation and cell-cycle control. In addition, it plays a central role in the control of cell proliferation and death in response to various urges like DNA damage, hypoxia, oxidative stress, DNA mutations and nutrient deprivation.^12^ Not surprisingly, TP53 gene alterations are frequent in a large proportion of human cancers, and occur in a tissue-specific manner. For example, TP53 mutation rates vary from 2.2% in renal cell carcinoma to 89% and 94.9% in endometrial carcinoma and serous ovarian cancers, respectively.^13^ Inherited TP53 mutations lead to a wide spectrum of early-onset cancers.^14^ In contrast to other tumour-suppressor genes that are mainly altered by truncating mutations, the majority of TP53 mutations are missense substitutions (75%). Other alterations include frameshift insertions and deletions (9%), nonsense mutations (7%), silent mutations (5%) and other infrequent alterations.^15^ However, whether different types of TP53 mutations bear distinct clinical and pathophysiological significance in HNSCC has not been elucidated so far.

Structurally, p53 is a multifunctional 393-residue protein; it is encoded by a gene localised on chromosome 17p13.1 and is composed of 25,772 bases. The protein is constituted by three subunits: N-terminal, Core domain and C-terminal. The N-terminal subunit is composed of a transactivation domain (residues 1–42) and a proline-rich domain (residues 63–97). The central core domain (residues 98–292) is composed of a single unit that contains sequence-specific DNA-binding activity of p53 (DNA-binding domain). The C-terminal domain is characterised of a flexible linker region (residues 293–323), a tetramerisation domain (residues 324–355) and C-terminal regulatory domain (residues 363–393) that undergoes a number of post-translational modifications such as acetylation and phosphorylation.^16,17^ A growing body of evidence now suggests that differential mutational profiles of TP53 gene can influence disease prognosis in several types of tumours; for example, distinct TP53 mutations are independent predictors of survival in CD20+ lymphomas.^18^ The same results have also been reported for ALK+ NSCLC,^19^ hepatocellular carcinoma, HNSCC, acute myeloid leukaemia, clear-cell renal cell carcinoma (RCC), papillary RCC, uterine endometrial carcinoma and thymoma.^20^ Whether mutations in p53 subdomains differentially affect disease prognosis, however, has not been elucidated so far.

The aim of this study was to investigate the mutational landscape of TP53, and to correlate these molecular features with clinical variables. To do so, we used a bioinformatics approach by analysing data from The Cancer Genome Atlas (TCGA) database.^21^ The results from this analysis revealed that a wide landscape of TP53 mutations exists in HNSCC and, for the first time, demonstrated that these mutations are associated with distinct clinical behaviour in a site-specific manner.

## Materials and methods

### Data source and data collection

This study has been performed according to the Recommended Guidelines for Validation, Quality Control and Reporting of TP53 Variants Clinical Practice.^22^

TCGA data have been accessed and downloaded through UCSC Xena Browser (https://xena.ucsc.edu/). Data for mRNA expression profile of TP53 gene (ENSG00000141510) were downloaded as RNAseq data HTSeq-Fragments Per Kilobase Million (FPKM)—dataset ID TCGA-HNSC/Xena_Matrices/TCGA-HNSC.htseq_fpkm.tsv, and the mutational profile with the variant-allele frequency (VAF), such as the type of mutation (MuTect2 Variant Aggregation and Masking—dataset ID TCGA-HNSC/Xena_Matrices/TCGA-HNSC.mutect2_snv.tsv). The mutation dataset was also download from the cBioPortal for cancer genomics website. This website was also useful for downloading the RPPA-Z-score expression of p53 protein (http://www.cbioportal.org).^23^ In this analysis, only patients with a single mutation of the TP53 gene were included. Patients with double mutations on the same gene or mismatching mutation type in the two datasets were excluded. At the end of the inclusion process, 415 patients’ profiles were eligible for statistical analysis. Genomic Data Commons (GDC) Data Portal (https://portal.gdc.cancer.gov/) was used to download clinical and follow-up information. Data were pasted and organised in SPSS 21.0 in order to perform the statistical analysis for the evaluation of clinical and prognostic correlations. The amino-acid sequence changes were assessed and used to classify mutation profiles according to the secondary structure of p53 protein. In the Research Collaboratory for Structural Bioinformatics Protein Data Bank (RCSB-PDB), it was possible to retrieve the Secondary Structure data for the TP53 protein (https://www.rcsb.org/pdb/explore/remediatedSequence.do?params.showJmol=false&structureId=3Q01).^24^

### Data grouping

Of 415 patients with HNSCC included in the study (Supplementary Table 1A–D), 129 patients had no mutations in the TP53 gene and were defined as “wild type” (WT); meanwhile, 286 patients had one single mutation in the gene sequence (MUT) (of these, 51 patients had a frameshift mutation, 8 an inframe mutation, 152 a missense mutation, 26 a splice-site mutation and 49 patients had a stop mutation). The p53 mRNA expression for 411 patients (log2 (fpkm + 1)) in the TCGA Database ranged between 0.71 and 6.47 with a mean of 3.740184 (S.D. 1.2168926) and a median of 3.88. The p53 RPPA-Z-score protein was available for only 173 patients and ranged from −4.3211 to 3.0036 with a mean of −0.001732 (S.D. 0.91044) and a median of −0.0933. Both for mRNA expression and RPPA-Z score, patients were classified as high and low expression using the median as threshold. It is important to note that RPPA-Z-score protein was available for only 173 patients, and it is not representative of the whole cohort.

Patients were also divided according to VAF, intended as the proportion of DNA molecules bringing the variant. From 40 to 64% VAF, patients were classified as being heterozygous loci; meanwhile, 65–100% VAF patients were grouped as homozygous loci.^25^ VAF was reported for 271 patients and ranged from 5 to 97% with a mean of 46.73% (S.D. 19.33%).

In order to further evaluate the relation of the location and the type of mutation with the clinicopathological characteristics, patients were divided according to

1. The position of the mutated base on the DNA sequence of the gene, such as in the N-terminal transactivation domain (residues 1–97), in the DNA-binding domain (residues 98–292) and in the C-terminal domain (residues 293–393).^16^ In total, 19 patients had a mutation in the N-terminal domain, 37 patients in the C-terminal domain and 230 patients in the DNA-binding domain. In total, 152 of 230 mutations in the DNA-binding domain were missense.
2. The secondary structure extracted from the RCSB-PDB, such as mutation affecting the helix region (3/10 helix, alpha-helix structure), a strand region (beta bridge, beta strand), a turn region (turn, bend)^26^ and an unknown region, for which no secondary structure is assigned.
3. Well-known hotspot mutations, such as the ones occurring in the residues 175, 245, 248, 273 and 282.^27^ In particular, residue R175 was affected in 5 patients, G245 in 7 patients, R248 in 11 patients, R273 in 14 patients and R282 in 6 patients (43 hotspot mutations/286 total mutations). In addition to these frequent spots, we decided to include new residues, which were also frequently mutated; only residues involved in at least six patients included in the cohort were also included as new hotspots mutations; such sites were H179 (seven patients), H193 (six patients), R196 (eight patients) and R213 (seven patients).
4. The residues involved in the zinc ion ligand, such as C176, H179, C238 and C242, which were involved in 17 on 286 patients.
5. Mutations were classified according to the type of single-amino-acid substitution, such as transition and transversion. In order to investigate random deamination^28^ or tobacco smoke- related mechanisms of mutations,^29^ transitions of C–T in CpG islands and transversions of G:C–T:A were also highlighted. In addition, mutations involving CpG sites, reported in http://p53.iarc.fr/p53Sequence.aspx, were also investigated.
6. Mutations in conserved residues were compared with their non-conserved sites, according to a previously reported analysis by Martin et al.^30^ Conserved residues were retrieved from their online platform at http://bioinf.org.uk/p53/analysis/index.html#conserved, such as pro98, phe113, lys120, ser121, val122, thr125, ser127, leu130, lys132, leu137, lys139, pro142, pro151, pro152, arg158, ala159, lys164, val172, val173, arg175, pro177, his178, his179, arg196, glu198, gly199, tyr205, asp208, ser215, val216, val218, pro219, tyr220, glu221, pro223, thr230, asn239, ser240, ser241, cys242, met243, gly244, gly245, asn247, arg249, ile251, thr253, leu257, gly262, leu265, gly266, arg267, phe270, glu271, val272, cys275, ala276, cys277, pro278, gly279, arg280, asp281 and arg282.
7. Martin et al. also characterised amino-acid substitutions according to their ability to donate or accept hydrogen bonds. The amino acids K, R and W are only able to donate H^+^; meanwhile, E and D are only able to accept hydrogen bonds. The amino acids H, N, Q, S, T and Y are both able to accept and donate as reported by Baker et al.^31^ Patients were classified as missense-disruptive mutation with substitution of K, R and W with E and D and vice versa, or in the case of forming H^+^ bond amino acids, substituted by non-forming H^+^ bonds.
8. We also categorised two further amino-acid substitutions, such as (1) mutations resulting in a substitution by proline, and (2) mutations from native glycine in residues at codons 117, 154, 187, 244, 245 and 262. These kinds of mutations, because of their sidechain features, are more restricted in the allowed conformations.^30^

a) All these analyses were performed in the TCGA database of patients with squamous cell carcinoma of the head and neck (HNSCC). Patients were further categorised into four main subgroups:^32^

1. Oral cavity (OC), such as alveolar ridge, buccal mucosa, floor of the mouth, hard palate, oral tongue, general oral cavity and lips
2. Oropharynx (OP), such as base of the tongue, oropharynx and tonsils
3. Hypopharynx (HP)
4. Larynx (L)

Data were also download for oesophagus and lung squamous cell carcinoma, in order to investigate and compare the TP53 mutational landscape among these groups of cancers. UCLA, TCGA and ICGC oesophagus databases from cBioPortal and TCGA (PanCancer Atlas) lung squamous cell carcinoma, were downloaded following the previous descripted criteria, including only patients with histologically confirmed squamous cell carcinoma.

### Interactome–genome–transcriptome network analysis based on co-alteration data

In order to highlight co-differential gene network between WT and MUT groups, we combined interactome–transcriptome analysis^33^ and interactome–genome analysis to construct dynamic, tumour-specific networks based on predicted changes of p53 interactors resulting from genetic (mutations, CNA) or transcriptional (mRNA expression) modifications occurring in the same HNSCC cohorts. Specifically, patients were compared according to WT/MUT and subsite (OC, OP, HP and L) to evaluate if different genes/pathways were modified in relation to the distinct mutational profile of TP53 at different subsites. cBioPortal gene network tool highlights alterations per each gene, such as mutations, CNA or mRNA dysregulation. Moreover, cBioPortal gene network tool was used to build the interactome by filtering the interactions according to “controls state change of”, “controls transport of”, “controls of phosphorylation of”, “control expression of”, “in complex with” and “neighbour of”. This analysis was also performed between HPV-positive and HPV-negative OP tumours. When more than 50 neighbour genes existed in the network, these were ranked by genomic alteration frequency within the selected group of patients. To provide an effective visualisation of networks that highlighted the most relevant genes to the query, we adopted an alteration frequency of 16.9% as cut-off^23^. For HP subgroup, a 33.3% cut-off was applied because of the low number of patients included. Gene ontology (GO) analysis to evaluate the main pathway alterations in each subgroup analysis was performed by using http://geneontology.org/ tool^34^ and retrieving the results from PANTHER.^35^

Gene, percentage and type of both alteration and cell function are summarised in Supplemental material (Supplementary Table 3A–J), by including only the results with a fold enrichment over ten.

### Statistical analysis

Because of the non-normal distribution of variables, non-parametric tests were used (normal distribution of variables was explored through Shapiro–Wilk normality test). Spearman rank- correlation analysis was performed to investigate the relation between the expression profile of p53, the mutational profile and the clinicopathological characteristics. For dichotomous variables, chi-square test was used. The difference in expression between groups was further investigated through the non-parametric test of Mann–Whitney or by Kruskal–Wallis one-way or two-way ANOVA test. Bonferroni–Holm false-discovery rate was applied to correct for multiple comparisons. Kaplan–Meier analysis with log-rank test was applied to explore differences in the overall and disease-free survival by univariate analysis. In order to estimate the effect of clinicopathological variables, a multivariate Cox regression model was built, including the following parameters as covariates: age, gender, staging and grading. All tests were performed by using SPSS 21.0 and STATA 16.0; only *P* < 0.05 results were considered statistically significant.

### Survival prediction algorithm

We generated an algorithm based on modifications of the algorithm previously reported by Poeta et al.^36^ In Poeta’s algorithm, patients are grouped as bringing a TP53-disruptive mutation versus conservative mutation. Stop, frameshift, inframe and splice mutations are classified as disruptive, together with missense mutations in L2–L3 segment of the protein (codons 163–195 and 236–251) with changes in charge or polarity of the substituted amino acid. Any missense mutation outside L2–L3 segment or in L2–L3 segment without changes in charge or polarity, are considered conservative. First, we applied this algorithm to TCGA head and neck cancer, and then we implemented this model in order to highlight patients at high risk of death, according to deleterious missense substitutions in the secondary structure of the protein. Mutations were reclassified as disruptive if

- in homozygous loci, such as DNA–VAF from 65 to 100%;^25^
- in zinc ligand involved;
- changing from K, R and W to E and D and vice versa, or in the case of forming H^+^ bond amino acids, substituted by non-forming H^+^ bonds;
- affecting an amino acid in a non-assigned secondary structure (unknown, as reported in RCSB-PDB http://www.rcsb.org/pdb/explore/remediatedSequence.do?structureId=1TUP).

Mutations were reclassified as conservative if assigned to any other secondary structure, when maintaining their ability to donate or accept hydrogen bonds. Harrell’s C-statistic, AIC (Akaike information criterion) and BIC (Bayesian information criterion) were used to assess possible improvements of the prediction model.

## Results

### Survival analysis of TP53 mutational landscape reveals novel prognostic signatures

**Fig. 1:**
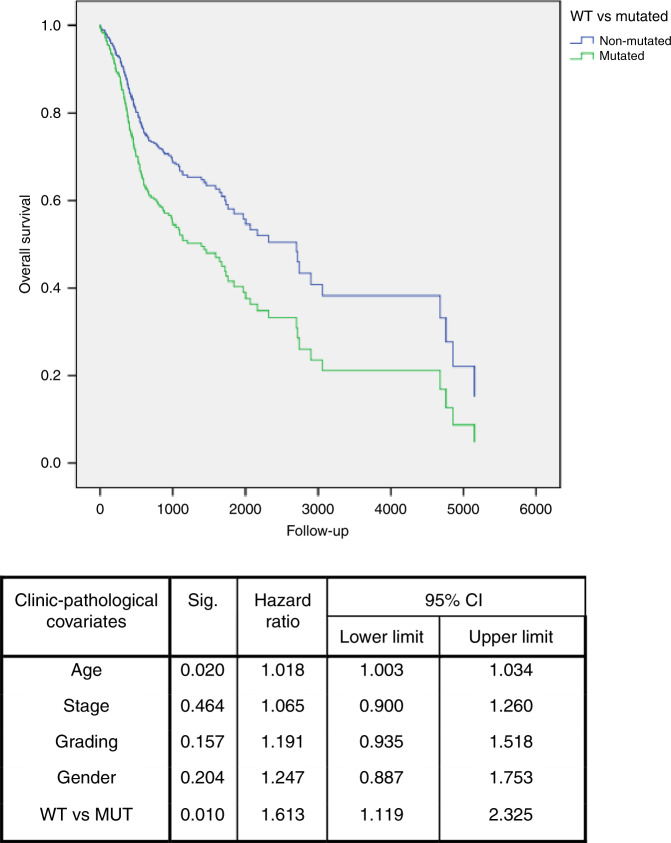
Overall survival of TP53-mutated patients. Multivariate survival analysis revealed that patients with mutations (MUT) in TP53 gene sequence (green line) showed a worse survival compared to patients with wild-type (WT) TP53 (blue line).

**Fig. 2:**
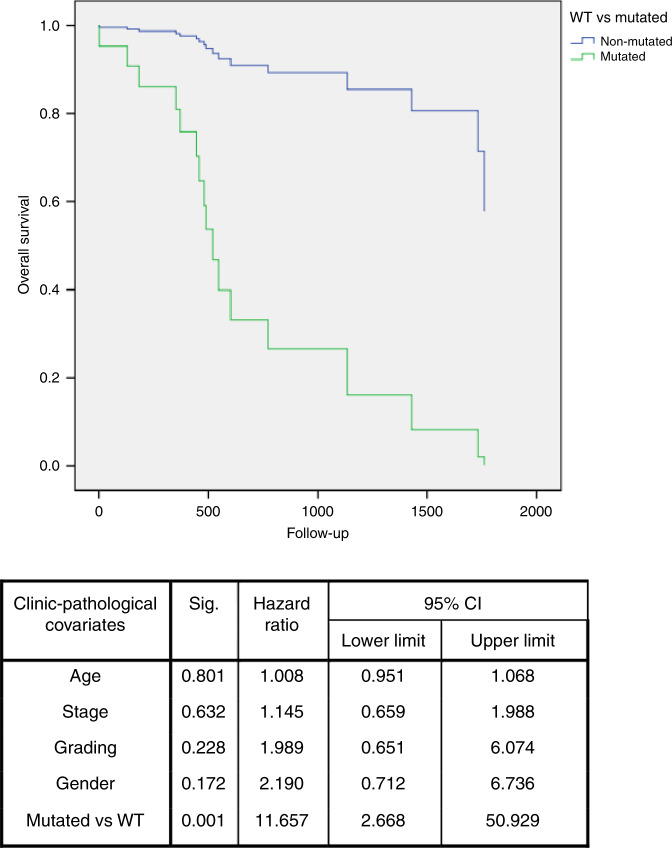
Overall survival of patients with oropharynx squamous cell carcinoma. Multivariate survival analysis, including only oropharynx squamous cell carcinoma, revealed that subjects with mutations (MUT) in TP53 gene sequence (green line) had a worse survival compared to patients with wild-type (WT) TP53 (blue line).

We aimed to investigate whether the presence of mutations in the TP53 gene correlated with the prognosis of HNSCC. By comparing wild-type (WT) HNSCCs with the ones with mutated TP53, univariate survival analysis showed a worse overall survival for patients carrying one mutation in TP53 gene. Multivariate Cox regression analysis confirmed that TP53 mutation was an independent prognostic factor in HNSCC patients (multivariate analysis: HR = 1.613; 95% CI: 1.119–2.325; *P* = 0.010) (Fig. 1). Interestingly, in the OP subgroup, the mutated profile was an independent prognostic factor of overall survival (multivariate analysis: HR = 11.657; 95% CI: 2.668–50.929; *P* = 0.001) (Fig. 2); meanwhile, it was close to the threshold of statistical significance for disease-free survival (multivariate analysis: HR = 5.773; 95% CI: 0.896–37.174; *P* = 0.065).

Next, we investigated whether there was an association between specific characteristics of the mutation and patients’ survival, as follows.

#### Structural domains

When analysing TP53 mutations according to the predicted p53 domains affected (N-terminal, C-terminal or DNA-binding domain), no differences in survival emerged in subgroups, except larynx, where at the univariate analysis, patients with mutations in the DNA-binding domain had a worse overall survival than those with mutations in the N-terminal segment of the gene (multivariate analysis: HR = 0.223; 95% CI: 0.050–0.998; *P* = 0.050) (Supplementary Fig. 1).

#### Secondary structure

**Fig. 3:**
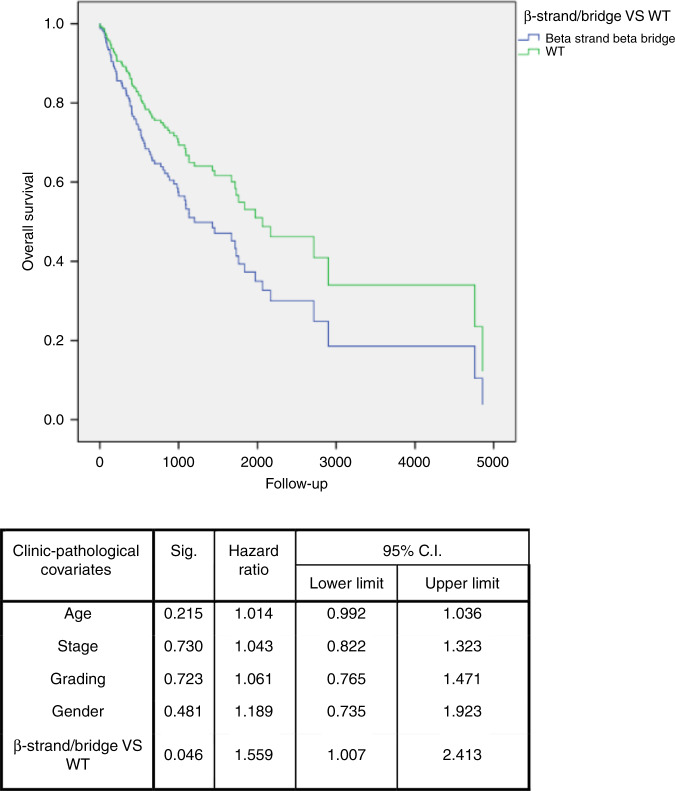
Overall survival of patients with TP53 mutations in β-strand/bridge. Multivariate survival analysis in head and neck squamous cell carcinoma, showing that patients with mutations in β-strand/bridge of p53 secondary structure (blue line) have a worse survival compared to patients with wild-type (WT) TP53 gene (green line).

TP53 mutations were then analysed according to their occurrence in the predicted secondary structure of the protein. No differences in survival emerged between WT patients and those with mutations in the helix or in turn region of the protein. Patients with mutations in a strand region had a worse overall survival, both in HNSCC (multivariate analysis: HR = 1.559; 95% CI: 1.007–2.413; *P* = 0.046) (Fig. 3) and larynx (multivariate analysis: HR 0.071; 95% CI: 0.005–0.935; *P* = 0.044) subgroups, compared with WT. Poor overall survival was also detected for patients with mutations in unknown regions. In particular, patients in HNSCC (multivariate analysis: HR = 2.476; 95% CI: 1.525–4.019; *P* < 0.001) (Supplementary Fig. 2), OP (multivariate analysis: HR = 0.072; 95% CI: 0.011–0.490; *P* = 0.007) and L (univariate analysis: HR = 0.133; 95% CI: 0.03–0.598; *P* = 0.008) subgroups had a worse overall survival compared with the WT group.

#### Hotspot mutations

Survival analysis was also performed in order to investigate whether particular hotspot mutations could influence patients’ prognosis. Specifically, the comparison was performed between hotspot mutations and non-hotspot residues. Mutations affecting the residue R175 were associated with a worse overall survival in HNSCC (multivariate analysis: HR = 6.855; 95% CI: 1.635–28.75; *P* = 0.008) compared with the non-hotspot group. The same result emerged from the analysis of the residue H193 (multivariate analysis: HR = 3.578; 95% CI: 1.380–9.277; *P* = 0.009). Finally, R213 was linked to poor overall survival in HNSCC (univariate analysis: *P* = 0.024) (Supplementary Fig. 3). Although the results from this analysis could be clinically relevant, it should be noted that the small sample size available for this cohort bears high risk of bias; therefore, the results should be further validated in larger sample size.

#### Type of mutation

Differences in mutation type (frameshift, missense, inframe, splice site and stop) showed variable prognostic capabilities. In particular, missense (multivariate analysis: HR = 1.688; 95% CI: 1.129–2.526; *P* = 0.011) and stop (multivariate analysis: HR = 2.016; 95% CI: 1.220–3.332; *P* = 0.006) mutations were predictive of worse overall survival compared with WT patients in HNSCC.

#### Variant-allele frequency

**Fig. 4:**
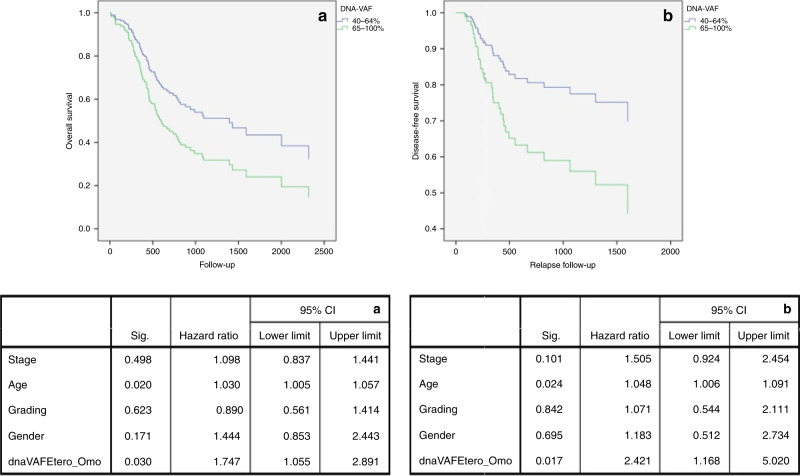
Overall and Relapse-free survival according to DNA-VAF. Multivariate survival analysis for patients with head and neck squamous cell carcinoma, showing that subjects with homozygous mutations in TP53 gene sequence (green line) showed a worse overall (**a**) and relapse-free (**b**) -survival, compared to patients with heterozygous mutations (blue line).

In the last analysis, HNSCC patients with higher VAF, such as those carrying the mutation in homozygous loci, reported a worse overall survival (multivariate analysis: HR = 1.747; 95% CI: 1.055–2.891; *P* = 0.030) and higher risk of relapse (multivariate analysis: HR = 2.421; 95% CI: 1.168–5.020; *P* = 0.017) compared with patients with lower VAF (i.e. mutations in heterozygous loci) (Fig. 4). Differential mRNA expression did not influence the overall survival in HNSCC and its subgroups.

Finally, because cancers arising in lung and oesophagus share common histopathological characteristics with HNSCC, we compared available datasets for these groups of carcinomas. Surprisingly, the results failed to show an association between p53 mutations and prognosis. In particular, in lung squamous cell cancer, patients with wild-type TP53 had worse overall survival compared with patients with mutated p53 (multivariate analysis: HR = 0.636; 95% CI: 0.437–0.926; *P* = 0.018). The results are summarised in Supplementary Table 2.

Taken together, these data demonstrate that TP53 mutations are not only predictors of patient survival but, also, that different types of mutations have distinct prognostic significance in HNSCC.

### TP53 genotype correlates with expression profile and clinicopathological variables

#### Correlation with TP53 transcripts

The expression of p53 mRNA differed between WT and MUT patients (Mann–Whitney *P* < 0.001) in HNSCC. In the WT cohort, the expression (log2 (fpkm + 1)) ranged from 0.71 to 6.47 with a mean of 4.2825 and a median of 4.39. In the MUT cohort, mRNA expression varied from 0.76 to 5.85 with a mean of 3.492 and a median of 3.665. This differential expression was also reported in the OP subgroup (Mann–Whitney *P* < 0.001) where in the WT cohort, the expression ranged from 2.23 to 6.47 with a mean of 5.1579 and a median of 5.21; meanwhile, for the MUT cohort, the expression reported was from 1.82 to 5.62 with a mean of 3.6789 and a median of 3.90.

mRNA expression was also variable in HPV^+^ and HPV^−^ tumours, in particular in OP subgroup (Mann–Whitney *P* = 0.006). In HPV^−^ tumours, the expression ranged with a mean of 3.86 and a median of 3.73; meanwhile, HPV^+^ tumours reported a mean of 5.2373 and a median of 5.1754. A two-way ANOVA was conducted that examined the effect of the anatomical subsite and mutational status on TP53 mRNA expression. There was a statistically significant interaction between the anatomical subsite and mutational status on mRNA expression, two-way ANOVA *P* = 0.003. Simple main effect analysis showed that mutational status significantly affected mRNA expression (*P* < 0.001), but there were no differences between anatomical subsites (*P* = 0.684). Bonferroni–Holm post hoc test showed a differential mRNA expression between OP and the other subsites (O, L and HP; *P* < 0.001) and a higher mRNA expression in WT, missense and inframe MUT, compared with frameshift, splice and stop MUT (*P* < 0.001) (Supplementary Fig. 4). mRNA expression poorly correlated with its protein expression (Spearman rank-correlation test ρ = 0.382, *P* < 0.001). Of interest, TP53 mRNA expression differed among tumour grades (Bonferroni–Holm post hoc test G1 vs G2 *P* = 0.045; G1 vs G3 *P* < 0.001; G2 vs G3 *P* = 0.031) with the following expression means in G1 of 3.2434, in G2 of 3.6753 and in G3 of 4.0424.

#### Correlation with clinicopathological variables

Chi-square test showed a differential ratio between WT/MUT patients and tumour subgroup (OC, OP, HP and L) and HPV positive/negative (*P* < 0.001). In total, 176/246 MUT in OC (Bonferroni post hoc test *P* = 0.08239), 19/62 in OP (Bonferroni post hoc test *P* < 0.000001), 5/9 in HP (Bonferroni post hoc test *P* = 0.40597) and 78/90 in L (Bonferroni post hoc test *P* = 0.00002); for the HPV status, 3/30 HPV-positive patients were mutated in TP53 gene; meanwhile, 52/64 were mutated in HPV-negative tumours. In addition, perineural invasion was more frequent in MUT (*P* = 0.031); 28/78 WT reported perineural invasion against 101/201 MUT. This event was also notable in the OC subgroup where 21/50 WT reported perineural invasion against 82/140 MUT (*P* = 0.044). Interestingly, 110/143 smoking patients were mutated against 176/272 non-smokers (*P* = 0.011).

Spearman analysis showed a correlation between DNA–VAF and grading in HNSCC (*ρ* = 0.131*, P* = 0.035), in particular higher DNA–VAF was present in patients with higher tumour grade (Kruskal–Wallis *P* = 0.041—Bonferroni–Holm post hoc test G1 vs G2 *P* = 0.134; G1 vs G3 *P* = 0.059; G2 vs G3 *P* = 1.00). The results from Spearman analysis in head and neck cancer are shown in Supplementary Fig. 5.

Different characteristics were also found between male and female patients. Chi-square test showed a higher RPPA-Z-score protein expression in males, compared with females, 65/119 males and 17/51 females (*P* = 0.011). In addition, the occurrence of hotspot mutations taken into consideration, differed between genders. Mutations in H193 occurred only in males; meanwhile, of 8 patients mutated in R196, 6/8 were female patients (chi-square test *P* = 0.003). R196 resulted mutated only in the OC subgroup. In the last analysis, R273 mutations were characterised by a missense mutation by a substitution of the R (arginine) amino acid to C (cysteine) or H (histidine). Changes in C involved one male over five patients; meanwhile, H involved seven male patients over nine (chi-square test *P* = 0.036). R273 missense mutations were also linked to alcohol consumption; in particular, of five patients with a change from R to C, four reported alcohol consumption in the anamnesis; meanwhile, 8/9 patients with H change did not report alcohol history (chi-square test *P* = 0.01); the same result was also detected for the OC subgroup (chi-square test *P* = 0.044).

Cigarette consumption was also considered as a clinical variable in HNSCC. Transitions of C–T in CpG islands are reported to be a common consequence of random deamination; meanwhile, transversions of G:C–T:A are tobacco smoke-related mechanisms of mutations.^28,29^ Chi-square test (*P* = 0.018) showed a lower number of transversion events in never-smoker patients (6/16) (Bonferroni post hoc test *P* = 0.00237). A higher number of transversion of G:C–T:A events occurred in current smokers (24/31) (Bonferroni post hoc test *P* = 0.1815) and in ex-smokers less than 15 years (20/25) (Bonferroni post hoc test *P* = 0.1423). Indeed, a higher number of smoked cigarettes emerged in patients with transversions, compared with patients with transitions of C–T in CpG islands (Mann–Whitney *P* = 0.032).

Patients with mutations in Alpha secondary structure showed lower number of smoked packs of cigarettes, against Turn and Bend (Mann–Whitney *P* = 0.005), unknown (Mann–Whitney *P* = 0.03) and β-strand/bridge patients (*P* = 0.021). Although Bonferroni–Holm post hoc test failed to find significant difference (*P* = 0.190; *P* = 0.288; *P* = 0.096, respectively), chi-square test showed a significant difference between the smoking history and the secondary structure involved. In particular, only 8/124 current smokers reported a mutation in Alpha secondary structure (*P* = 0.019). Bonferroni post hoc-adjusted *P* values are reported in Supplementary Table 4.

Collectively, our data show distinct correlations between TP53 genotype, p53 expression profile and clinicopathological features of HNSCC.

### Network analysis reveals distinct alterations in HNSCC subgroups

Interactome–genome–transcriptome analysis was undertaken to build a dynamic network that highlighted the TP53 interactors that underwent genomic (mutations, CNA) or translational (mRNA expression) modifications in HNSCC. TP53 networks differed substantially in WT- and p53-mutated HNSCC subgroups (Supplementary Fig. 6–15).

In cancer arising from the oral cavity, there were several molecules differentially involved in TP53 network (Supplementary Figs. 6 and 7, Table 3A/B). Both WT and MUT TP53 groups shared common alterations of CDKN2A, TP63 and DROSHA. In WT, the main modification involved the cellular response to DNA-damage stimulus, with changes affecting PMS2, CDK9, DDB2 and EPHA2. In MUT, changes in NDRG1, GSK3B, SNAI2, BCL6, CCNK, PRKDC and RRM2B affected mainly the intrinsic apoptotic process and the transition of the G1/S cell-cycle phase.

In HNSCC of the oropharynx (Supplementary Figs. 8, 9, Table 3C/D), both WT and MUT were associated with alterations in BCL6, TP63, GSK3B, CDKN2A, CCNK and DROSHA. Surprisingly, CDKN2A in WT resulted altered only in 18.2% of cases (13.6% reported mRNA upregulation, 2.3% homozygous deletion and 2.3% mutation), whereas in MUT, CDKN2A was affected in 78.9% of cases (57.9% reported homozygous deletion and 21.1% mutation). In particular, WT group resulted affected by deficiencies in both intrinsic and extrinsic apoptotic signals, cell-cycle growth checkpoint at G1/S phase, mismatch repair, positive histone deacetylation, negative regulation of cell–matrix adhesion, fatty acid biosynthetic process, cellular response to starvation, negative regulation of intracellular oestrogen receptor signalling pathway, morphogenesis of embryonic epithelium and negative regulation of phosphatidylinositol 3-kinase signalling. These mechanisms are regulated in particular by PCNA, BCL6, FAS, GSK3B, TP63, TSC2, BRCA1, MDM2, PTEN, TP73, DYRK1A, MSH2, PRKAB1, PRKDC, CDK1, MDM4, RRM2B and E2F2.

DGR8, AGO4 and DROSHA regulated primary miRNA processing, involved in gene silencing in the WT OP subgroup.

CX3CL1 and MYB showed common alteration in both WT TP53 OP and HPV^+^ OP subgroups, with positive regulation of transforming growth factor beta production.

BCL2, TRIM28 and CCNK emerged to be involved in the regulation of viral genome replication, sharing common alterations in both WT TP53 OP and HPV^+^ OP group.

In MUT, there were different molecules involved in the TP53 network. Noncoding RNA (ncRNA) transcription was linked to alterations in CCNK and CDK9, and in particular, HIF1A and YY1 resulted in a positive regulation of pri-miRNA transcription by RNA polymerase II. CCNK and CDK9 were found also in the HPV^–^ OP subgroup. In terms of pathways, the main alterations involved the apoptotic process, due to alterations in CDKN2A, NDRG1, TP63, BCL6, PRKAB2, PPP2CB, PRMT5, HIF1A, KAT5 and TTC5. Notably, alterations resulted in positive regulation of glycolytic process, beta-catenin-TCF complex assembly, regulation of cellular respiration and positive regulation of epithelial cell proliferation, led by TP63, MYC, KAT5 and HIF1A. The main alterations involved autophagy mechanisms, due to changes in PRKAB2, HIF1A, KAT5 and GSK3B. At last, changes in SERPINE1, GSK3B and TP63 resulted in modifications in epithelial differentiation.

In HNSCC of the larynx (Supplementary Figs. 10, 11, Table 3E/F), CDKN2A, BCL6, TP63, GSK3B and NDRG1 were altered in both WT and MUT; CSNK2A1 and CREBBP were downregulated in WT, whereas the same resulted upregulated in MUT. In terms of pathways, the main alteration in WT affected the signal transduction by p53-class mediators and downstream stress-activated MAPK cascade, due to modifications affecting MAPK13, UBB, TRAF6, MAPKAPK2 and DYRK1A.

In MUT, a defect emerged in the crosstalk between regulation of cell growth and cellular response to hypoxia and gamma radiation, as suggested by the alteration of PRKDC, MYC, COP1, CREBBP, NDRG1 and WRN.

Alterations in the hypopharynx subgroups differed between wild-type and mutated TP53 groups (Supplementary Figs. 14, 15, Table 3I/J). CDKN2A showed common alterations in both groups. In the WT group, PLK3, BCL2L1, CX3CL1, BAX and NGFR all resulted in upregulation in their mRNA expression, with some cases of amplification or mutation. These were mainly involved in the negative regulation of apoptotic process, and in the regulation of cell-cycle G1/S-phase transition. Interestingly, CX3CL1 resulted participating in positive regulation of calcium-independent cell-to-cell adhesion, regulation of stem cell proliferation and regulation of cell–matrix adhesion. NGFR seems to be related to the positive regulation of pri-miRNA transcription by RNA polymerase II and together with CDKN2A, in RAS protein signal transduction.

MUT group showed a higher number of alterations, and CDKN2A was mainly affected. DDIT4 resulted in mRNA upregulation in 60% of cases, affecting the intrinsic apoptotic signalling pathway in response to DNA damage by p53-class mediator and negative regulation of ATP metabolic process. Alterations in metabolic pathways could be of interest in this group of patients because of changes in fatty acid biosynthetic process, vasoconstriction, ATP metabolic process and rhythmic process involving EDN2, PRKAG1, HTT, CSNK2A1, HGF, HIF1A, PRKAB2 and SNAI2. Of interest in this group are also changes in chromatin assembly and silencing because of mRNA upregulation in 40% of cases of HIST1H1D and downregulation of HIRA.

In addition, differences in the TP53 network between HPV^−^ and HPV^+^ HNSCCs were compared. OP HPV^−^ tumours showed common alterations with HPV^+^ tumours of the same subsite (Supplementary Figs. 12, 13, Table 3G/H). In particular, TP63, BCL6, GSK3B, DROSHA and CCNK showed similar modifications. CDKN2A mRNA resulted in upregulation in 22.2% of HPV^+^ tumours, whereas HPV^−^ tumours showed homozygous deletion and mutations in 50% and 33.3%, respectively.

In HPV^−^, a wide number of molecules were involved in a negative regulation of cell–matrix adhesion, stem cell differentiation, negative regulation of epithelial cell differentiation and regulation of intracellular oestrogen receptor signalling pathway. Of interest, an alteration in lipid metabolism emerged in alterations in PRKAB2 and PRKAA2, with consequences in lipophagy, carnitine shuttle and fatty acid transmembrane transport.

HPV^+^ showed modifications of both intrinsic and extrinsic apoptotic processes, due to alterations in histone phosphorylation, peptidyl–threonine phosphorylation, peptidyl–serine phosphorylation and protein autophosphorylation processes, which lead to changes in ubiquitination. Notably, in this cohort, there was an upregulation of PCNA mRNA in 48.1% of samples.

The complete list of altered nodes and their function is reported in Supplementary Table 3A-J and Figs. 6–15.

Taken together, these data show that distinct TP53 molecular networks are associated with HNSCC in a site- and mutation-specific manner. Notwithstanding these differences in molecular pathways, all HNSCC tumours share a common alteration landscape in the crosstalk between cellular stress response, cell-cycle progression and apoptotic process.

### Survival prediction algorithm results

**Fig. 5:**
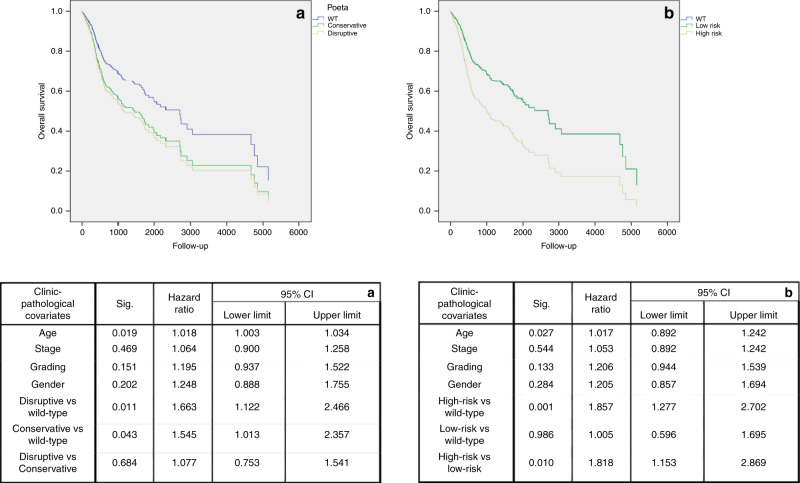
Overall survival for algorithms of TP53 mutational status. Multivariate survival analysis of patients with head and neck squamous cell carcinoma, showing differences among patients with disruptive (yellow line) and conservative (green line) mutations in TP53 gene according to Poeta's classification (**a**); and patients with high risk (yellow line) and low risk (green line) death mutations, according to our new classification system (**b**). Blue lines are, in both of cases, representative of wild-type (WT) patients.

By applying the Poeta algorithm (PA)^36^ on the TCGA database, we found that disruptive mutations had independent prognostic significance in HNSCC, although with small difference between disruptive and conservative mutations (disruptive vs conservative mutations, multivariate analysis: HR = 1.077; 95% CI: 0.753–1.541; *P* = 0.684); (disruptive vs wild-type, multivariate analysis: HR = 1.663; 95% CI: 1.122–2.466; *P* = 0.011); (conservative vs wild-type, multivariate analysis: HR = 1.545; 95% CI: 1.013–2.357; *P* = 0.043) (Fig. 5a). In addition, we integrated the biochemical information from PA to the ones from Martin et al.^30^ with the addition of our findings according to the predicted secondary structure and the number of mutated alleles. Patients were classified as carriers of high-risk death mutations, carriers of low-risk death mutations and wild type. Our model successfully identified patients at higher risk of death according to the mutational status, depending on the biochemical alterations, characteristics and predicted secondary structure. High risk of death mutations resulted to be an independent prognostic factor in TCGA head and neck database, with greater difference towards low risk of death mutations (high-risk vs low-risk mutations, multivariate analysis: HR = 1.818; 95% CI: 1.153–2.869; *P* = 0.010); (high-risk vs wild-type, multivariate analysis: HR = 1.857; 95% CI: 1.277–2.702; *P* = 0.001); (low-risk vs wild-type, multivariate analysis: HR = 1.005; 95% CI: 0.596–1.695; *P* = 0.986) (Fig. 5b). The results of Bonferroni post hoc multiple comparisons for both multivariate survival analyses are reported in Supplementary Table 5A/B.

Harrell’s C-statistic, Akaike information criterion (AIC) and Bayesian information criterion (BIC) were then used to compare the predictive performance of our model with the PA algorithm. C-statistic, AIC and BIC resulted to be 0.5400, 1829.253 and 1837.29, respectively, using the PA. The results for our model reported a Harrell’s C-statistic of 0.5700, AIC of 1819.828 and BIC of 1823.847. These results showed that our model performed better than the one published by Poeta (Very strong improvement ΔBIC = 13.443).^37^ The newly developed algorithm was applied to each subsite, namely OC, OP and L, as well as lung and oesophagus (HP was excluded because of the small number of samples available). As mentioned above, the dichotomous classification of mutational status was an independent prognostic factor only in OP. PA and our algorithm also performed well in this subgroup (PA: wild-type vs disruptive, multivariate analysis: HR = 0.082; 95% CI: 0.017–0.385; *P* = 0.002; our algorithm: wild-type vs high-risk, multivariate analysis: HR = 0.082; 95% CI: 0.017–0.402; *P* = 0.002). In OSCC, PA failed to find any significant prognostic class (wild-type vs disruptive, multivariate analysis: HR = 0.771; 95% CI: 0.483–1.232; *P* = 0.277 and conservative vs disruptive mutations, multivariate analysis: HR = 0.815; 95% CI: 0.520–1.277; *P* = 0.372); meanwhile, our algorithm found a class of mutation with a better overall survival (wild-type vs high-risk, multivariate analysis: HR = 0.714; 95% CI: 0.458–1.113; *P* = 0.137); (low-risk vs high-risk, multivariate analysis: HR = 0.499; 95% CI: 0.283–0.878; *P* = 0.016).

**Fig. 6:**
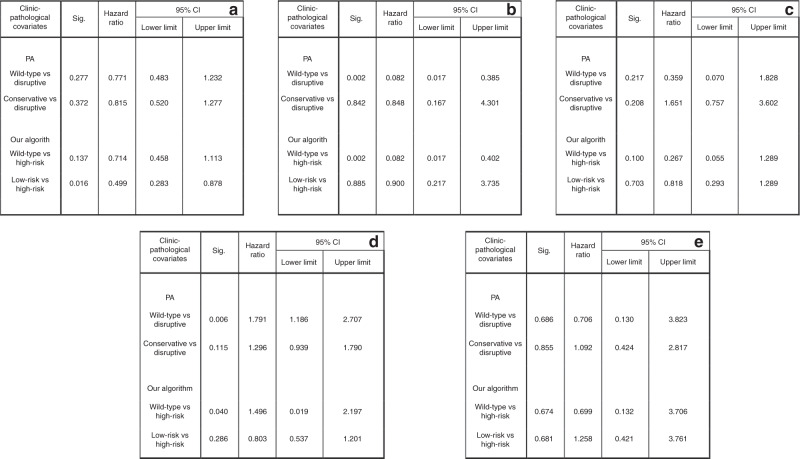
Overall survival of Poeta's versus our new proposed classification system. Multivariate survival analysis in patient with squamous cell carcinoma of: oral cavity (**a**); oropharynx (**b**); larynx (**c**); lung (**d**) and oesophagus (**e**). Differences in survival are shown according to Poeta's algorithm (PA) and to our new algorithm.

In L and oesophagus, both algorithms failed to find any significant results, while lung tumours showed a unique behaviour. Specifically, wild-type p53 was associated with a worse overall survival compared with the whole group of patients carrying mutations (multivariate analysis: HR = 1.572; 95% CI: 1.080–2.287; *P* = 0.018). When the PA was applied, it emerged that patients with disruptive mutations had a better overall survival, compared both with wild-type (multivariate analysis: HR = 1.791; 95% CI: 1.186–2.707; *P* = 0.006) and nondisruptive mutated patients (multivariate analysis: HR = 1.296; 95% CI: 0.939–1.790; *P* = 0.115). Our algorithm, meanwhile, was able to distinguish a group of high-risk mutations (multivariate analysis: HR = 0.803; 95% CI: 0.537–1.201; *P* = 0.286), although wild-type patients still reported the worst overall survival compared with low risk of death mutations (multivariate analysis: HR = 1.496; 95% CI: 1.019–2.197; *P* = 0.040). The results from both algorithms in each subsite are summarised in Fig. 6.

## Discussion

Many efforts have been made to classify mutations according to their influence on structural changes, and to investigate if they serve as prognostic factors, but limits have been identified due to the wideness of the mutational landscape of TP53. In this study, we propose a new classification method that identifies patients with mutations at high risk of death in squamous cell cancers and, in particular, in tumours from the head and neck district.

Of the 14 million new cancers diagnosed worldwide every year, 50–60% is characterised by at least one somatic variant of p53.^13^ In the HNSCC cohort from TCGA included in this study, 69.9% of patients expressed a mutation in TP53. A web platform has been created to collect and organise the increasing number of researches published about TP53 in cancer (http://www.p53.fr/).^15^

Due to the increase in detection of single mutations in TP53 gene, several studies have attempted to elucidate the correlation between mutational status and patients’ clinicopathological characteristics, with discordant results. Most published studies have employed different classifications and mutation profiles for their analyses. This is a reasonable approach, since a broad range of mutations can affect the TP53 gene and its encoded protein; for example, the 286 HNSCC patients included in our study exhibited 129 different kinds of mutation, of which R273 was the most frequent but occurred in only 13 patients.

One of the simplest and most used approach to translate TP53 mutational profile into clinically useful information is to compare wild-type and mutated patients, with the latter subgroup predicting death in different types of cancers. An extensive analysis of 33 TCGA studies showed that the effects of TP53 mutations on patients’ prognosis were statistically significant in nine malignancies (lung adenocarcinoma, hepatocellular carcinoma, HNSCC, acute myeloid leukaemia, clear-cell renal cell carcinoma (RCC), papillary RCC, chromophobe RCC, uterine endometrial carcinoma and thymoma).^20^ Although this method can be considered “quick and useful”, this approach does not take into account some biochemical and functional characteristics of single TP53 mutations. A previous comprehensive genomic study on the same TCGA cohort provided fundamental insights into the correlation between mutational profile of TP53 and HNSCC; however it did not take into consideration the anatomical sublocalisation of tumours in the HNSCC area, in which HPV-positive oropharynx subgroup frequently exhibits wild-type TP53 and favourable prognosis.^32^ After integrating the survival analysis according to the subsite of HNSCC onset, mutated TP53 resulted to be an independent prognostic factor for overall and disease-free survival only in OP. These results have salient clinical implications, because, cells with wild-type or higher TP53 expression are more susceptible to radiation therapy, and HPV^+^ tumours usually display higher radiosensitivity.^38^ Therefore, our data suggest that mutations in TP53 gene have a prognostic role in HNSCC, above all, in HPV^+^ OP tumours where mutational status of this gene should be investigated before considering treatment options.^39^ These findings could let us speculate that mutations affecting TP53 in HPV^+^ tumours make them more similar to HPV^−^ HNSCCs. Although this hypothesis should be analysed in future studies, the results of network analysis showed that MUT OP shared common alterations with other subgroups, in particular homozygous deletions and mutations affecting CDKN2A (78.9% of patients in MUT OP against 13.6% mRNA upregulation in WT OP). Because of the interaction between HPV E7 protein and host cells, CDKN2A mRNA upregulation was also observed in HPV^+^ tumours arising in the oropharynx. As it is known, HPV E7 protein ubiquitinates the protein of retinoblastoma (pRb) by binding to the cullin 2 ubiquitin ligase complex. Loss of pRb leads to the release of E2F with the transcription of S-phase genes. Hence, HPV^+^ tumours show an upregulation of CDKN2A as a consequence of negative feedback loop to control cell cycle, from pRb loss.^40^ In addition, CX3CL1 and MYB shared common alteration (mRNA upregulation) both in WT OP and HPV^+^ OP, leading to a positive regulation of transforming growth factor beta production. They could be involved in a cell-response mechanism due to the effect of E7 proteins. It is reported that E7 is able to prevent both Smad transcriptional activity and the ability of TGF-β to inhibit DNA synthesis.^41^ MYB has been shown to be related to HPV infection, above all in cervical cancer,^42,43^ but its role, together with CX3CL1, has never been elucidated in OP. It is worth noting that BRCA1 was also upregulated in WT OP patients and HPV^+^ tumours (29.5% and 29.6%, respectively). From our network analysis, BRCA1 was present in both subgroups in complex with the proliferating cell nuclear antigen (PCNA). PCNA is a protein produced in S phase of the cell cycle, and it acts in replication and repair machinery, favouring the cell-cycle progression.^44^ PCNA seems to be an important factor for progression to malignancy in HPV^+^ tumours, by activation of S phase of the cell cycle.^45^ BRCA1 is involved in genome-integrity machinery and cell-cycle checkpoint control.^46^ BRCA1 plays a critical role in homologous recombination repair, and cells with deficiency in BRCA1 are more sensible to drugs causing DNA breaks or to ionising radiation.^47^ Tian et al. showed that BRCA1 leads to mono- and polyubiquitination of PCNA by recruiting some effector proteins. It is reported that PCNA monoubiquitylation is necessary for efficient translesion synthesis. Through this mechanism, BRCA1 promotes translesion DNA synthesis and progression of the cell cycle.^48,49^ Taken together, these findings suggest that BRCA1 mRNA upregulation with PCNA could have an important role in HPV + tumours of oropharynx and in chemo-radioresistance of these patients, by promoting translesion DNA synthesis and cell-cycle progression, changing its classical function as tumour suppressor to an oncogene, as already reported in cancer stem cell models of different kinds of tumours.^50^

Similar to what was performed in the previously cited genomic analysis,^20^ Poeta et al.^36^ applied their algorithm only for the whole HNSCC cohort without investigating the results for each subgroup. The reported algorithm was able to prognostically stratify HNSCC patients; in particular, using the wild-type group as reference, only tumours with disruptive mutations showed a worse overall survival, whereas patients with conservative or nondisruptive mutations did not. Similar results were obtained in this study by applying the PA on the TCGA HNSCC cohort (Fig. 5). In addition, conservative mutations were also linked to a worse overall survival, although without differences between disruptive and nondisruptive mutations. Starting from the findings of Poeta et al, we developed our own algorithm reclassifying mutations in high risk of death according to their homozygous alteration, their zinc ligand and H^+^-forming bond alteration. Martin et al. and Baker et al.^30,31^ already stressed the important role of hydrogen bonding in protein residues. Hence, for example, the mutation Y220C is considered nondisruptive in the PA, since in its tertiary structure, this residue is located far from the functional part. This mutation was reclassified as high risk since tyrosine (Y) is able to create a hydrogen bonding, conversely to what happens when substituted by a cysteine (C). The same can be stated for the residues involved in the zinc ligand, since it is important for the stabilisation of the p53/DNA complex.^30^ At last, mutations affecting residues in “unknown” predicted secondary structure were considered as high risk. Comparing the predictive capability of the two algorithms on the TCGA database, our model outperformed the PA. In particular, our classifier was able to better stratify a cohort of patients with higher risk of death, comparing it with both wild-type and nondisruptive mutation groups, while the PA was not able to find a significant difference between nondisruptive and disruptive mutations (Fig. 5). Subsequently, we investigated the predictive performance of both algorithms in each subgroup. Of interest, PA failed to find any significant prognostic class in OSCC, where the new model found a class of mutations with a better overall survival (wild-type vs high-risk, multivariate analysis: HR = 0.714; 95% CI: 0.458–1.113; *P* = 0.137); (low-risk vs high-risk, multivariate analysis: HR = 0.499; 95% CI: 0.283–0.878; *P* = 0.016). However, both algorithms failed to find any significant prognostic factor in larynx, with contradictory results in lung and oesophageal cancer. A meta-analysis, published in 2015,^51^ reported the same conflicting results in non-small-cell lung carcinoma, since TP53 mutations emerged to be associated with a worse overall survival compared with wild type. Although the meta-analysis included all patients with non-small-cell lung carcinoma, when performing subgroup analysis, only patients with early stages and affected by adenocarcinoma, took advantage of the wild-type status. The same results were reported in other studies.^52,53^ Molina-Vila et al.^54^ were the only to apply Poeta et al. classification^36^ in a cohort of advanced-stage non-small-cell lung cancer; only nondisruptive mutations were associated with a shorter survival. Our results cannot be compared, since we included only patients with squamous cell carcinoma, and our cohort consisted of only 80/438 patients with advanced stage (III–IV); because of these promising results, we included all patients in a whole cohort, including patients with head and neck-, oesophageal- and lung squamous cell carcinoma. Patients included in the survival analysis were 914 with complete data about survival status, follow-up time, mutational status, staging, age and gender (grading was removed because it was only available for head and neck patients). Of these, 249 were from oral cavity, 62 from oropharynx, 89 from larynx, 9 from hypopharynx, 72 oesophagheal cancers and 433 lung squamous cell cancers. In the multivariate Cox regression model, the dichotomous mutational status (WT/MUT) did not correlate with overall survival (multivariate analysis: HR = 1.174; 95% CI: 0.912–1.511; *P* = 0.214); we therefore applied both classification systems on the new cohort. PA failed to find any significant association between disruptive and conservative mutations with the overall survival (wild-type vs disruptive mutations, multivariate analysis: HR = 1.125; 95% CI: 0.856–1.478; *P* = 0.397) (wild-type vs nondisruptive mutations, multivariate analysis: HR = 1.239; 95% CI: 0.937–1.639; *P* = 0.133). Nondisruptive mutations showed even a worse overall survival compared with disruptive mutations (nondisruptive vs disruptive mutations, multivariate analysis: HR = 1.101; 95% CI: 0.880–1.378; *P* = 0.399). Conversely, the new proposed algorithm showed a better predictive performance; in particular, patients in the high-risk group showed a worse prognosis, while the low-risk group showed even a better overall survival compared with wild type (wild-type vs high-risk mutations, multivariate analysis: HR = 1.283; 95% CI: 0.991–1.663; *P* = 0.059); (wild-type vs low-risk mutations, multivariate analysis: HR = 0.881; 95% CI: 0.629–1.236; *P* = 0.464); (low- vs high-risk mutations, multivariate analysis: HR = 0.687; 95% CI: 0.518–0.911; *P* = 0.009).

For the first time, our study elucidated the mutational profile of TP53 gene in HNSCC subgroups. To the best of our knowledge, this was the first study to link different molecular aspects of TP53 alterations (mutational profile of TP53, coding gene structure, secondary structure and well-known hotspot mutations) to the clinical variables of HNSCC patients. Although most tumours arising from the mucosa of the head and neck district are studied together, the results from this study clearly show differences between the OC, OP and L subsites in terms of mutational profile and signalling pathways of TP53. Furthermore, this study suggests that there is a broad range of TP53 residues that could be mutated in HNSCC, which may determine differential effects in terms of mRNA and protein expression, secondary structure, apoptosis activity and DNA-binding affinity. This finding makes it difficult to develop drugs that target selective mutations of TP53 as these would have little implications in the clinical management of HNSCC patients. Finally, whilst this study indicates a prognostic role of TP53 mutations in HNSCC, the influence of TP53 status in cancer prognosis more broadly is still controversial and large, and well-standardised studies are needed.

## Supplementary information

Supplemental material

Suppl. Figures

## Data Availability

All data are downloadable from the following URLS: http://www.cbioportal.org, https://portal.gdc.cancer.gov/https://xena.ucsc.edu/.

## References

[1] Leemans CR, Braakhuis BJ, Brakenhoff RH (2011). The molecular biology of head and neck cancer. Nat. Rev. Cancer.

[2] Ali J, Sabiha B, Jan HU, Haider SA, Khan AA, Ali SS (2017). Genetic etiology of oral cancer. Oral. Oncol..

[3] Siegel RL, Miller KD, Jemal A (2016). Cancer statistics, 2016. CA Cancer J. Clin..

[4] Ferlay J, Soerjomataram I, Dikshit R, Eser S, Mathers C, Rebelo M (2015). Cancer incidence and mortality worldwide: sources, methods and major patterns in GLOBOCAN 2012. Int. J. Cancer.

[5] Hecht SS (2003). Tobacco carcinogens, their biomarkers and tobacco-induced cancer. Nat. Rev. Cancer.

[6] Menzies GE, Reed SH, Brancale A, Lewis PD (2015). Base damage, local sequence context and TP53 mutation hotspots: a molecular dynamics study of benzo[a]pyrene induced DNA distortion and mutability. Nucleic Acids Res..

[7] Tuna M, Amos CI, Mills GB (2019). Genome-wide analysis of head and neck squamous cell carcinomas reveals HPV, TP53, smoking and alcohol-related allele-based acquired uniparental disomy genomic alterations. Neoplasia.

[8] Li XC, Wang MY, Yang M, Dai HJ, Zhang BF, Wang W (2018). A mutational signature associated with alcohol consumption and prognostically significantly mutated driver genes in esophageal squamous cell carcinoma. Ann. Oncol..

[9] Mirghani H, Lacroix L, Rossoni C, Sun R, Auperin A, Casiraghi O (2018). Does smoking alter the mutation profile of human papillomavirus-driven head and neck cancers?. Eur. J. Cancer.

[10] Holloway E (2002). From genotype to phenotype: linking bioinformatics and medical informatics ontologies. Comp. Funct. Genomics..

[11] Finlay CA, Hinds PW, Levine AJ (1989). The p53 proto-oncogene can act as a suppressor of transformation. Cell.

[12] Kamada R, Toguchi Y, Nomura T, Imagawa T, Sakaguchi K (2016). Tetramer formation of tumor suppressor protein p53: Structure, function, and applications. Biopolymers.

[13] Kandoth C, McLellan MD, Vandin F, Ye K, Niu B, Lu C (2013). Mutational landscape and significance across 12 major cancer types. Nature.

[14] Petitjean A, Mathe E, Kato S, Ishioka C, Tavtigian SV, Hainaut P (2007). Impact of mutant p53 functional properties on TP53 mutation patterns and tumor phenotype: lessons from recent developments in the IARC TP53 database. Hum. Mutat..

[15] Olivier M, Eeles R, Hollstein M, Khan MA, Harris CC, Hainaut P (2002). The IARC TP53 database: new online mutation analysis and recommendations to users. Hum. Mutat..

[16] Bernard X, Robinson P, Nomine Y, Masson M, Charbonnier S, Ramirez-Ramos JR (2011). Proteasomal degradation of p53 by human papillomavirus E6 oncoprotein relies on the structural integrity of p53 core domain. PLoS ONE.

[17] Chen Y, Dey R, Chen L (2010). Crystal structure of the p53 core domain bound to a full consensus site as a self-assembled tetramer. Structure.

[18] Zenz T, Kreuz M, Fuge M, Klapper W, Horn H, Staiger AM (2017). TP53 mutation and survival in aggressive B cell lymphoma. Int. J. Cancer.

[19] Christopoulos, P., Dietz, S., Kirchner, M., Volckmar, A. L., Endris, V., Neumann, O. et al. Detection of TP53 mutations in tissue or liquid rebiopsies at progression identifies ALK+ lung cancer patients with poor survival. *Cancers* **11**, 124 (2019). 10.3390/cancers11010124 PMC6356563 30669647

[20] Li, V. D., Li, K. H. & Li, J. T. TP53 mutations as potential prognostic markers for specific cancers: analysis of data from The Cancer Genome Atlas and the International Agency for Research on Cancer TP53 Database. *J. Cancer Res. Clin. Oncol.* **145**, 625–636 (2019). 10.1007/s00432-018-2817-z PMC11810387 30542790

[21] Wang Z, Jensen MA, Zenklusen JC (2016). A practical guide to the cancer genome atlas (TCGA). Methods Mol. Biol..

[22] Leroy B, Ballinger ML, Baran-Marszak F, Bond GL, Braithwaite A, Concin N (2017). Recommended guidelines for validation, quality control, and reporting of TP53 variants in clinical practice. Cancer Res..

[23] Gao J, Aksoy BA, Dogrusoz U, Dresdner G, Gross B, Sumer SO (2013). Integrative analysis of complex cancer genomics and clinical profiles using the cBioPortal. Sci. Signal..

[24] Rose PW, Prlic A, Altunkaya A, Bi C, Bradley AR, Christie CH (2017). The RCSB protein data bank: integrative view of protein, gene and 3D structural information. Nucleic Acids Res..

[25] Strom SP (2016). Current practices and guidelines for clinical next-generation sequencing oncology testing. Cancer Biol. Med..

[26] Moon, S., Balch, C., Park, S., Lee, J., Sung, J. & Nam S. Systematic inspection of the clinical relevance of TP53 missense mutations in gastric cancer. *IEEE/ACM Trans. Comput. Biol. Bioinform.* **16**, 1693–1701 (2018). 10.1109/TCBB.2018.2814049 29994072

[27] Tahara T, Shibata T, Okamoto Y, Yamazaki J, Kawamura T, Horiguchi N (2016). Mutation spectrum of TP53 gene predicts clinicopathological features and survival of gastric cancer. Oncotarget.

[28] Ambs S, Bennett WP, Merriam WG, Ogunfusika MO, Oser SM, Harrington AM (1999). Relationship between p53 mutations and inducible nitric oxide synthase expression in human colorectal cancer. J. Natl Cancer Inst..

[29] Tretyakova N, Matter B, Jones R, Shallop A (2002). Formation of benzo[a]pyrene diol epoxide-DNA adducts at specific guanines within K-ras and p53 gene sequences: stable isotope-labeling mass spectrometry approach. Biochemistry.

[30] Martin AC, Facchiano AM, Cuff AL, Hernandez-Boussard T, Olivier M, Hainaut P (2002). Integrating mutation data and structural analysis of the TP53 tumor-suppressor protein. Hum. Mutat..

[31] Baker EN, Hubbard RE (1984). Hydrogen bonding in globular proteins. Prog. Biophys. Mol. Biol..

[32] Cancer Genome Atlas N. (2015). Comprehensive genomic characterization of head and neck squamous cell carcinomas. Nature.

[33] Cirillo N, Prime SS (2009). Desmosomal interactome in keratinocytes: a systems biology approach leading to an understanding of the pathogenesis of skin disease. Cell Mol. Life Sci..

[34] Mi H, Muruganujan A, Ebert D, Huang X, Thomas PD (2019). PANTHER version 14: more genomes, a new PANTHER GO-slim and improvements in enrichment analysis tools. Nucleic Acids Res..

[35] Thomas PD, Campbell MJ, Kejariwal A, Mi H, Karlak B, Daverman R (2003). PANTHER: a library of protein families and subfamilies indexed by function. Genome Res..

[36] Poeta ML, Manola J, Goldwasser MA, Forastiere A, Benoit N, Califano JA (2007). TP53 mutations and survival in squamous-cell carcinoma of the head and neck. N. Engl. J. Med..

[37] Raftery AE, Chunn JL, Gerland P, Sevcikova H (2013). Bayesian probabilistic projections of life expectancy for all countries. Demography.

[38] Eriksson D, Stigbrand T (2010). Radiation-induced cell death mechanisms. Tumour Biol..

[39] Smith EM, Wang D, Rubenstein LM, Morris WA, Turek LP, Haugen TH (2008). Association between p53 and human papillomavirus in head and neck cancer survival. Cancer Epidemiol. Biomark. Prev..

[40] Chung CH, Gillison ML (2009). Human papillomavirus in head and neck cancer: its role in pathogenesis and clinical implications. Clin. Cancer Res..

[41] Lee DK, Kim BC, Kim IY, Cho EA, Satterwhite DJ, Kim SJ (2002). The human papilloma virus E7 oncoprotein inhibits transforming growth factor-beta signaling by blocking binding of the Smad complex to its target sequence. J. Biol. Chem..

[42] Tian Y, Chen H, Qiao L, Zhang W, Zheng J, Zhao W (2018). CIP2A facilitates the G1/S cell cycle transition via B-Myb in human papillomavirus 16 oncoprotein E6-expressing cells. J. Cell Mol. Med..

[43] Martin CM, Astbury K, Kehoe L, O’Crowley JB, O’Toole S, O’Leary JJ (2015). The use of MYBL2 as a novel candidate biomarker of cervical cancer. Methods Mol. Biol..

[44] Branca M, Ciotti M, Giorgi C, Santini D, Di Bonito L, Costa S (2007). Up-regulation of proliferating cell nuclear antigen (PCNA) is closely associated with high-risk human papillomavirus (HPV) and progression of cervical intraepithelial neoplasia (CIN), but does not predict disease outcome in cervical cancer. Eur. J. Obstet. Gynecol. Reprod. Biol..

[45] Park JS, Rhyu KS, Kim CJ, Kim HS, Han KT, Ahn HK (1996). Presence of oncogenic HPV DNAs in cervical carcinoma tissues and pelvic lymph nodes associating with proliferating cell nuclear antigen expression. Gynecol. Oncol..

[46] O’Donovan PJ, Livingston DM (2010). BRCA1 and BRCA2: breast/ovarian cancer susceptibility gene products and participants in DNA double-strand break repair. Carcinogenesis.

[47] Moynahan ME, Chiu JW, Koller BH, Jasin M (1999). Brca1 controls homology-directed DNA repair. Mol. Cell..

[48] Tian F, Sharma S, Zou J, Lin SY, Wang B, Rezvani K (2013). BRCA1 promotes the ubiquitination of PCNA and recruitment of translesion polymerases in response to replication blockade. Proc. Natl Acad. Sci. USA.

[49] Gervai JZ, Galicza J, Szeltner Z, Zamborszky J, Szuts D (2017). A genetic study based on PCNA-ubiquitin fusions reveals no requirement for PCNA polyubiquitylation in DNA damage tolerance. DNA Repair.

[50] Gorodetska I, Kozeretska I, Dubrovska A (2019). BRCA genes: the role in genome stability, cancer stemness and therapy resistance. J. Cancer.

[51] Gu J, Zhou Y, Huang L, Ou W, Wu J, Li S (2016). TP53 mutation is associated with a poor clinical outcome for non-small cell lung cancer: evidence from a meta-analysis. Mol. Clin. Oncol..

[52] Fukuyama Y, Mitsudomi T, Sugio K, Ishida T, Akazawa K, Sugimachi K (1997). K-ras and p53 mutations are an independent unfavourable prognostic indicator in patients with non-small-cell lung cancer. Br. J. Cancer.

[53] Kashii T, Mizushima Y, Lima C, Noto H, Sato H, Saito H (1995). Evaluation of prognostic-significance of p53 gene alterations in patients with surgically resected lung-cancer. Int. J. Oncol..

[54] Molina-Vila MA, Bertran-Alamillo J, Gasco A, Mayo-de-las-Casas C, Sanchez-Ronco M, Pujantell-Pastor L (2014). Nondisruptive p53 mutations are associated with shorter survival in patients with advanced non-small cell lung cancer. Clin. Cancer Res..

