# Supplementary material for: Computational analysis of TP53 mutational landscape unveils key prognostic signatures and distinct pathobiological pathways in head and neck squamous cell cancer
Source: Br J Cancer. 2020 Jul 20;123(8):1302–14. doi: 10.1038/s41416-020-0984-6 (PMC7553957; doi:10.1038/s41416-020-0984-6)
Supplement: Supplementary file 1 — Supplemental material [file 41416_2020_984_MOESM1_ESM.docx]

Supplemental material Table 1:

1. Clinic-pathological characteristics of included patients with squamous cell carcinoma of the oral cavity (OC);
2. Clinic-pathological characteristics of included patients with squamous cell carcinoma of the larynx (L);
3. Clinic-pathological characteristics of included patients with squamous cell carcinoma of the oropharynx (OP).
4. Clinic-pathological characteristics of included patients with squamous cell carcinoma of the hypopharynx (HP);

| **Clinic-pathological information** | **Groups** | **Number of patients (OC)** |
| --- | --- | --- |
| Age | ≤ 65 years old | 153/245 |
|  | > 65 years old | 92/245 |
| Gender | Male | 162/246 |
|  | Female | 84/246 |
| Grade | 1-2 | 192/244 |
|  | 3 | 52/244 |
| Stage | I-II | 74/241 |
|  | III-IV | 167/241 |
| Subsite | Alveolar ridge | 15/246 |
|  | Buccal mucosa | 15/246 |
|  | Floor of the mouth | 46/246 |
|  | Hard palate | 5/246 |
|  | Oral cavity | 58/26 |
|  | Oral tongue | 104/246 |
|  | Lips | 3/246 |
| Mutational status for TP53 | Wild-type | 70/246  A |
|  | Mutated | 176/246 |

| **Clinic-pathological information** | **Groups** | **Number of patients (L)** |
| --- | --- | --- |
| Age | ≤ 65 years old | 60/90 |
|  | > 65 years old | 30/90 |
| Gender | Male | 72/90 |
|  | Female | 18/90 |
| Grade | 1-2 | 65/86 |
|  | 3 | 21/86 |
| Stage | I-II | 11/86 |
|  | III-IV | 75/86 |
| Mutational status for TP53 | Wild-type | 12/90  B |
|  | Mutated | 78/90 |

| **Clinic-pathological information** | **Groups** | **Number of patients (OP)** |
| --- | --- | --- |
| Age | ≤ 65 years old | 50/62 |
|  | > 65 years old | 12/62 |
| Gender | Male | 52/62 |
|  | Female | 10/62 |
| Grade | 1-2 | 33/52 |
|  | 3 | 19/52 |
| Stage | I-II | 15/60 |
|  | III-IV | 45/60 |
| Subsite | Base of the tongue | 20/62 |
|  | Oropharynx | 6/62 |
|  | Tonsils | 36/62 |
| HPV Status | Negative | 6/32 |
|  | Positive | 26/32 |
| Mutational status for TP53 | Wild-type | 43/62  C |
|  | Mutated | 19/62 |

| **Clinic-pathological information** | **Groups** | **Number of patients (HP)** |
| --- | --- | --- |
| Age | ≤ 65 years old | 6/9 |
|  | > 65 years old | 3/9 |
| Gender | Male | 6/9 |
|  | Female | 3/9 |
| Grade | 1-2 | 5/9 |
|  | 3 | 4/9 |
| Stage | I-II | 0/9 |
|  | III-IV | 9/9 |
| HPV Status | Negative | 2/3 |
|  | Positive | 1/3 |
| Mutational status for TP53 | Wild-type | 4/9  D |
|  | Mutated | 5/9 |

Supplemental material Table 2:

Univariate and multivariate overall and disease-free survival for head and neck, oral, oropharynx, hypopharynx, larynx, oesophagus and lung squamous cell carcinoma. HR=Hazard Ratio; p=*p-value*; N/A not calculable.

| Variable | ***Subsite*** | **Univariate Overall Survival** | **Multivariate Overall Survival** | **Univariate Disease-Free Survival** | **Multivariate Disease-Free Survival** |
| --- | --- | --- | --- | --- | --- |
|  |  |  |  |  |  |
| Wild-type TP53 vs Mutated | ***Head and Neck*** | **HR = 1,586; p = 0,009** | **HR = 1,525; p = 0,026** | HR = 1,132; p = 0,641 | HR = 1,195; p = 0,535 |
|  |  |  |  |  |  |
|  | ***Oral*** | HR = 1,107; p = 0,638 | HR = 1,171; p = 0,475 | HR = 0,856; p = 0,632 | HR = 0,844; p = 0,611 |
|  |  |  |  |  |  |
|  | ***Oropharynx*** | **HR = 6,669; p = 0,001** | **HR = 11,657; p = 0,001** | **HR = 4,441; p = 0,037** | **HR = 5,773; p = 0,065** |
|  |  |  |  |  |  |
|  | ***Hypopharynx*** | HR = 1,691; p =0,669 | HR = 1,200; p = 0,914 | HR = 1,266; p = 0,848 | HR = 1,395; p = 0,801 |
|  |  |  |  |  |  |
|  | ***Larinx*** | HR = 1,669; p = 0,124 | HR = 3,639; p = 0,108 | HR = 1,032; p = 0,968 | HR = 0,585; p = 0,638 |
|  |  |  |  |  |  |
|  | ***Oesophagus*** | HR = 1,183; p = 0,658 | HR = 1,452; p = 0,660 | HR = 0,835; p = 0,805 | HR = 0,468; p = 0,362 |
|  |  |  |  |  |  |
|  | ***Lung*** | **HR = 0,655; p =0,026** | **HR = 0,636; p =0,018** | HR = 1,012; p =0,975 | HR = 0,994; p =0,988 |
|  |  |  |  |  |  |
| Low vs high mRNA expression | ***Head and Neck*** | HR = 0,966; p = 0,820 | HR = 1,091; p = 0,581 | HR = 0,918; p = 0,715 | HR = 1,115; p = 0,651 |
|  |  |  |  |  |  |
|  | ***Oral*** | HR = 1,056; p = 0,774 | HR = 1,038; p = 0,847 | HR = 1,208; p = 0,513 | HR = 1,302; p = 0,273 |
|  |  |  |  |  |  |
|  | ***Oropharynx*** | **HR =0,41; p = 0,002** | HR = 0,001; p = 0,909 | HR = 0,278; p = 0,112 | HR = 0,210; p = 0,149 |
|  |  |  |  |  |  |
|  | ***Hypopharynx*** | HR = 2,377; p = 0,480 | HR = 2,344; p = 0,691 | HR = 1,818; p = 0,629 | HR = 4,316; p = 0,563 |
|  |  |  |  |  |  |
|  | ***Larinx*** | HR = 1,669; p = 0,124 | **HR = 2,142; p = 0,063** | HR = 1,041; p = 0,942 | HR = 2,569; p = 0,164 |
|  |  |  |  |  |  |
|  | ***Oesophagus*** | HR = 0,612; p = 0,338 | HR = 0,917; p = 0,886 | HR = 0,912; p = 0,849 | HR = 1,152; p = 0,783 |
|  |  |  |  |  |  |
|  | ***Lung*** | HR = 0,897; p =0,459 | HR = 0,941; p =0,685 | HR = 0,941; p =0,820 | HR = 1,014; p =0,960 |
|  |  |  |  |  |  |
| DNA-VAF | ***Head and Neck*** | **HR = 1,677; p = 0,031** | **HR = 1,747; p = 0,030** | **HR = 2,079; p = 0,042** | **HR = 2,421; p = 0,017** |
|  |  |  |  |  |  |
|  | ***Oral*** | HR = 1,652; p = 0,143 | HR = 1,469; p = 0,285 | HR = 1,933; p = 0,201 | **HR = 2,844; p = 0,061** |
|  |  |  |  |  |  |
|  | ***Oropharynx*** | **HR = 14,065; p = 0,025** | **HR = 111,994; p = 0,023** | N/A | N/A |
|  |  |  |  |  |  |
|  | ***Hypopharynx*** | N/A | N/A | N/A | N/A |
|  |  |  |  |  |  |
|  | ***Larinx*** | HR = 1,931; p =0,136 | **HR = 2,660; p = 0,056** | HR = 3,227; p = 0,155 | HR = 0,479; p = 2,637 |
|  |  |  |  |  |  |
|  | ***Oesophagus*** | HR = 1,669; p = 0,294 | HR = 0,613; p = 0,514 | HR = 0,580; p = 0,253 | HR = 0,739; p = 0,606 |
|  |  |  |  |  |  |
|  | ***Lung*** | HR = 1,250; p = 0,317 | HR = 1,359; p = 0,176 | HR = 1,406; p = 0,321 | HR = 1,558; p = 0,204 |
|  |  |  |  |  |  |
| Mutated in Zinc ligand vs mutated in other region | ***Head and Neck*** | HR = 1,044; p = 0,913 | HR = 0,899; p = 0,786 | HR = 0,466; p = 0,078 | HR = 0,505; p = 0,205 |
|  |  |  |  |  |  |
|  | ***Oral*** | HR = 1,654; p = 0,398 | HR = 1,024; p = 0,968 | HR = 0,436; p = 0,086 | HR = 0,487; p = 0,248 |
|  |  |  |  |  |  |
|  | ***Oropharynx*** | N/A | N/A | N/A | N/A |
|  |  |  |  |  |  |
|  | ***Hypopharynx*** | N/A | N/A | N/A | N/A |
|  |  |  |  |  |  |
|  | ***Larinx*** | HR =0,624; p =0,440 | HR =0,796; p =0,721 | N/A | N/A |
|  |  |  |  |  |  |
|  | ***Oesophagus*** | HR = 0,595; p = 0,393 | HR = 0,774; p = 0,810 | **HR = 0,313; p = 0,070** | HR = 0,357; p = 0,122 |
|  |  |  |  |  |  |
|  | ***Lung*** | HR = 0,834; p = 0,562 | HR = 0,919; p = 0,791 | HR = 0,700; p = 0,550 | HR = 0,930; p = 0,907 |
|  |  |  |  |  |  |
| Mutated in Zinc ligand vs WT | ***Head and Neck*** | HR = 0,637; p = 0,275 | HR = 0,496; p = 0,118 | HR = 0,394; p = 0,052 | HR = 0,610; p = 0,415 |
|  |  |  |  |  |  |
|  | ***Oral*** | HR = 1,393; p = 0,587 | HR = 0,959; p = 0,947 | HR = 0,491; p = 0,188 | HR = 0,504; p = 0,336 |
|  |  |  |  |  |  |
|  | ***Oropharynx*** | N/A | N/A | N/A | N/A |
|  |  |  |  |  |  |
|  | ***Hypopharynx*** | N/A | N/A | N/A | N/A |
|  |  |  |  |  |  |
|  | ***Larinx*** | HR =0,198; p =0,080 | HR =0,009; p =0,168 | N/A | N/A |
|  |  |  |  |  |  |
|  | ***Oesophagus*** | HR = 0,757; p = 0,691 | N/A | HR = 0,422; p = 0,356 | N/A |
|  |  |  |  |  |  |
|  | ***Lung*** | HR = 1,291; p = 0,464 | HR = 1,572; p = 0,209 | HR = 0,722; p = 0,634 | HR = 1,212; p = 0,798 |
|  |  |  |  |  |  |
| α-Helix vs Wild Type | ***Head and Neck*** | HR = 0,816; p = 0,418 | HR = 0,775; p = 0,422 | HR = 0,789; p = 0,533 | HR = 0,976; p = 0,956 |
|  |  |  |  |  |  |
|  | ***Oral*** | HR = 1,160; p = 0,677 | HR = 1,031; p = 0,937 | HR = 1,131; p = 0,804 | HR = 1,243; p = 0,722 |
|  |  |  |  |  |  |
|  | ***Oropharynx*** | **HR = 0,178; p = 0,047** | HR = 0,256; p = 0,185 | HR = 0,067; p = 0,059 | HR = 0,005; p = 0,117 |
|  |  |  |  |  |  |
|  | ***Hypopharynx*** | N/A | N/A | N/A | N/A |
|  |  |  |  |  |  |
|  | ***Larinx*** | HR =0,397; p =0,245 | HR =0,682; p =0,746 | HR =0,786; p =0,794 | HR =0,725; p =0,818 |
|  |  |  |  |  |  |
|  | ***Oesophagus*** | HR = 0,820; p = 0,713 | HR = 3,480; p = 0,564 | HR = 2,992; p = 0,371 | N/A |
|  |  |  |  |  |  |
|  | ***Lung*** | **HR = 1,830; p =0,053** | **HR = 1,830; p = 0,055** | HR = 1,089; p = 0,871 | HR = 1,151; p = 0,789 |
|  |  |  |  |  |  |
| α-Helix vs Turn;bend | ***Head and Neck*** | HR = 0,990; p = 0,977 | HR = 1,233; p = 0,595 | HR = 0,871; p = 0,754 | HR = 1,104; p = 0,843 |
|  |  |  |  |  |  |
|  | ***Oral*** | HR = 1,028; p = 0,950 | HR = 1,141; p = 0,804 | HR = 0,841; p = 0,764 | HR = 0,945; p = 0,942 |
|  |  |  |  |  |  |
|  | ***Oropharynx*** | HR = 0,474; p = 0,546 | HR = 0,226; p = 0,899 | HR = 1,618; p = 0,697 | N/A |
|  |  |  |  |  |  |
|  | ***Hypopharynx*** | N/A | N/A | N/A | N/A |
|  |  |  |  |  |  |
|  | ***Larinx*** | HR =1,438; p =0,658 | HR =2,620; p =0,358 | N/A | N/A |
|  |  |  |  |  |  |
|  | ***Oesophagus*** | HR = 1,139; p = 0,803 | HR = 1,185; p = 0,860 | HR = 2,043; p = 0,523 | HR = 2,189; p = 0,501 |
|  |  |  |  |  |  |
|  | ***Lung*** | HR = 1,275; p = 0,446 | HR = 1,345; p = 0,357 | HR = 1,290; p = 0,608 | HR = 1,497; p = 0,421 |
|  |  |  |  |  |  |
| α-Helix vs Unknow | ***Head and Neck*** | **HR = 1,930; p = 0,026** | HR = 1,660; p = 0,130 | HR = 0,978; p = 0,957 | HR = 1,119; p = 0,808 |
|  |  |  |  |  |  |
|  | ***Oral*** | HR = 1,495; p = 0,296 | HR = 1,273; p = 0,572 | HR = 0,788; p = 0,694 | HR = 0,763; p = 0,727 |
|  |  |  |  |  |  |
|  | ***Oropharynx*** | HR = 1,007; p = 0,994 | N/A | HR = 0,283; p = 0,379 | N/A |
|  |  |  |  |  |  |
|  | ***Hypopharynx*** | N/A | N/A | N/A | N/A |
|  |  |  |  |  |  |
|  | ***Larinx*** | **HR = 3,856; p =0,036** | HR =3,233; p =0,138 | HR = 1,578; p = 0,553 | HR = 1,800; p = 0,494 |
|  |  |  |  |  |  |
|  | ***Oesophagus*** | HR = 1,153; p = 0,769 | HR = 1,263; p = 0,778 | HR = 4,630; p = 0,158 | HR = 4,834; p = 0,199 |
|  |  |  |  |  |  |
|  | ***Lung*** | HR = 1,777; p = 0,071 | **HR = 2,124; p = 0,024** | HR = 0,764; p = 0,649 | HR = 0,888; p = 0,844 |
|  |  |  |  |  |  |
| α-Helix vs β-Strand;brdige | ***Head and Neck*** | HR = 1,248; p = 0,439 | HR = 1,111; p = 0,726 | HR = 0,719; p = 0,389 | HR = 0,821; p = 0,645 |
|  |  |  |  |  |  |
|  | ***Oral*** | HR = 1,314; p = 0,439 | HR = 1,108; p = 0,788 | HR = 0,916; p = 0,860 | HR = 1,059; p = 0,925 |
|  |  |  |  |  |  |
|  | ***Oropharynx*** | HR = 0,010; p = 0,440 | HR = 0,001; p = 0,403 | HR = 0,703; p = 0,775 | N/A |
|  |  |  |  |  |  |
|  | ***Hypopharynx*** | N/A | N/A | N/A | N/A |
|  |  |  |  |  |  |
|  | ***Larinx*** | HR =1,619; p =0,464 | HR =1,961; p =0,461 | HR = 0,419; p = 0,292 | HR = 0,914; p = 0,920 |
|  |  |  |  |  |  |
|  | ***Oesophagus*** | HR = 0,757; p = 0,563 | HR = 0,626; p = 0,567 | HR = 4,061; p = 0,192 | HR = 2,646; p = 0,433 |
|  |  |  |  |  |  |
|  | ***Lung*** | HR =1,164; p =0,565 | HR =1,161; p =0,619 | HR =1,007; p =0,988 | HR =1,108; p =0,825 |
|  |  |  |  |  |  |
| β-Strand;brdige vs Wild Type | ***Head and Neck*** | **HR = 0,632; p = 0,032** | **HR = 0,641; p = 0,046** | HR = 1,020; p = 0,952 | HR = 0,963; p = 0,916 |
|  |  |  |  |  |  |
|  | ***Oral*** | HR = 0,855; p = 0,543 | HR = 0,893; p = 0,663 | HR = 1,177; p = 0,680 | HR = 1,174; p = 0,690 |
|  |  |  |  |  |  |
|  | ***Oropharynx*** | **HR = 0,226; p = 0,044** | HR = 0,701; p = 0,753 | HR = 0,297; p = 0,197 | HR = 0,213; p = 0,276 |
|  |  |  |  |  |  |
|  | ***Hypopharynx*** | N/A | N/A | N/A | N/A |
|  |  |  |  |  |  |
|  | ***Larinx*** | **HR = 0,184; p = 0,037** | **HR = 0,071; p = 0,044** | HR = 1,217; p = 0,833 | HR = 1,129; p = 0,935 |
|  |  |  |  |  |  |
|  | ***Oesophagus*** | HR = 1,032; p = 0,943 | HR = 0,944; p = 0,966 | HR = 0,835; p = 0,824 | HR = 1,688; p = 0,593 |
|  |  |  |  |  |  |
|  | ***Lung*** | HR = 1,641; p = 0,026 | **HR = 1,721; p = 0,022** | HR = 1,112; p = 0,807 | HR = 1,049; p = 0,913 |
|  |  |  |  |  |  |
| β-Strand;brdige vs Turn;bend | ***Head and Neck*** | HR = 0,751; p = 0,325 | HR = 0,908; p = 0,751 | HR = 1,141; p = 0,738 | HR = 0,984; p = 0,969 |
|  |  |  |  |  |  |
|  | ***Oral*** | HR = 0,685; p = 0,281 | HR = 0,751; p = 0,439 | HR = 0,917; p = 0,860 | HR = 0,704; p = 0,486 |
|  |  |  |  |  |  |
|  | ***Oropharynx*** | HR = 2,399; p = 0,388 | N/A | HR = 4,215; p = 0,244 | N/A |
|  |  |  |  |  |  |
|  | ***Hypopharynx*** | N/A | N/A | N/A | N/A |
|  |  |  |  |  |  |
|  | ***Larinx*** | HR =1,090; p =0,897 | HR =1,541; p =0,552 | HR = 0,848; p = 0,887 | HR = 2,205; p = 0,594 |
|  |  |  |  |  |  |
|  | ***Oesophagus*** | HR = 1,548; p = 0,287 | HR = 2,930; p = 0,240 | HR = 0,743; p = 0,649 | HR = 1,042; p = 0,955 |
|  |  |  |  |  |  |
|  | ***Lung*** | HR = 1,047; p = 0,843 | HR = 1,019; p = 0,936 | HR = 1,275; p = 0,548 | HR = 1,222; p = 0,638 |
|  |  |  |  |  |  |
| β-Strand;brdige vs Unknow | ***Head and Neck*** | **HR = 1,651; p = 0,023** | HR = 1,566; p = 0,064 | HR = 1,297; p = 0,487 | HR = 1,203; p = 0,646 |
|  |  |  |  |  |  |
|  | ***Oral*** | HR = 1,169; p = 0,597 | HR = 1,239; p = 0,490 | HR = 0,873; p = 0,797 | HR = 0,690; p = 0,504 |
|  |  |  |  |  |  |
|  | ***Oropharynx*** | HR = 4,288; p = 0,216 | N/A | N/A | N/A |
|  |  |  |  |  |  |
|  | ***Hypopharynx*** | N/A | N/A | N/A | N/A |
|  |  |  |  |  |  |
|  | ***Larinx*** | **HR =3,167; p =0,011** | HR =1,689; p =0,363 | HR = 3,491; p = 0,104 | HR = 2,659; p = 0,281 |
|  |  |  |  |  |  |
|  | ***Oesophagus*** | HR = 1,694; p = 0,147 | **HR = 11,811; p = 0,019** | HR = 1,043; p = 0,941 | HR = 1,371; p = 0,647 |
|  |  |  |  |  |  |
|  | ***Lung*** | HR = 1,520; p = 0,068 | **HR = 1,638; p = 0,40** | HR = 0,901; p = 0,840 | HR = 0,756; p = 0,609 |
|  |  |  |  |  |  |
| Turn;bend vs Wild Type | ***Head and Neck*** | HR = 0,800; p = 0,456 | HR = 0,753; p = 0,376 | HR = 0,863; p = 0,709 | HR = 0,935; p = 0,876 |
|  |  |  |  |  |  |
|  | ***Oral*** | HR = 1,148; p = 0,701 | HR = 1,047; p = 0,903 | HR = 1,197; p = 0,720 | HR = 1,076; p = 0,886 |
|  |  |  |  |  |  |
|  | ***Oropharynx*** | **HR = 0,155; p = 0,032** | **HR = 0,079; p = 0,029** | **HR = 0,031; p = 0,005** | HR = 0,001; p = 0,075 |
|  |  |  |  |  |  |
|  | ***Hypopharynx*** | N/A | N/A | N/A | N/A |
|  |  |  |  |  |  |
|  | ***Larinx*** | N/A | N/A | N/A | N/A |
|  |  |  |  |  |  |
|  | ***Oesophagus*** | HR = 0,760; p = 0,576 | HR = 5,900; p = 0,348 | HR = 1,762; p = 0,517 | HR = 1,853; p = 0,590 |
|  |  |  |  |  |  |
|  | ***Lung*** | HR =1,529; p =0,095 | HR =1,577; p =0,077 | HR =0,849; p =0,731 | HR =0,935; p =0,893 |
|  |  |  |  |  |  |
| Turn;bend vs Unknow | ***Head and Neck*** | **HR = 2,062; p = 0,016** | HR = 1,622; p = 0,127 | HR = 1,067; p = 0,881 | HR = 1,196; p = 0,709 |
|  |  |  |  |  |  |
|  | ***Oral*** | HR = 1,486; p = 0,308 | HR = 1,343; p = 0,477 | HR = 0,880; p = 0,834 | HR = 1,034; p = 0,959 |
|  |  |  |  |  |  |
|  | ***Oropharynx*** | HR = 1,510; p = 0,662 | N/A | HR = 0,134 p = 0,104 | N/A |
|  |  |  |  |  |  |
|  | ***Hypopharynx*** | N/A | N/A | N/A | N/A |
|  |  |  |  |  |  |
|  | ***Larinx*** | HR =0,361; p =0,266 | HR =0,001; p =0,959 | HR = 0,845; p = 0,906 | HR = 0,00; p = 0,440 |
|  |  |  |  |  |  |
|  | ***Oesophagus*** | HR = 1,204; p = 0,665 | HR = 1,004; p = 0,996 | HR = 1,708; p = 0,428 | HR = 1,974; p = 0,333 |
|  |  |  |  |  |  |
|  | ***Lung*** | HR = 1,384; p =0 ,210 | **HR = 1,680; p = 0,051** | HR = 0,698; p = 0,513 | HR = 0,764; p = 0,629 |
|  |  |  |  |  |  |
| Unknow vs Wild Type | ***Head and Neck*** | **HR = 0,040; p = 0,000** | **HR = 0,404; p = 0,000** | HR = 0,793; p = 0,532 | HR = 0,877; p = 0,744 |
|  |  |  |  |  |  |
|  | ***Oral*** | HR = 0,704; p = 0,250 | HR = 0,711; p = 0,291 | HR = 1,410; p = 0,515 | HR = 1,610; p = 0,399 |
|  |  |  |  |  |  |
|  | ***Oropharynx*** | **HR = 0,174; p = 0,023** | **HR = 0,072; p = 0,007** | HR = 0,405; p = 0,467 | HR = 0,123; p = 0,251 |
|  |  |  |  |  |  |
|  | ***Hypopharynx*** | HR =0,270; p = 0,292 | HR =0,00; p = 0,965 | HR =0,342; p = 0,385 | HR =0,000; p = 0,291 |
|  |  |  |  |  |  |
|  | ***Larinx*** | **HR = 0,133; p = 0,008** | HR = 0,143; p = 0,092 | HR = 0,313; p = 0,303 | HR = 0,124; p = 0,262 |
|  |  |  |  |  |  |
|  | ***Oesophagus*** | HR = 0,698; p = 0,412 | **HR = 0,050; p = 0,058** | HR = 0,684; p = 0,644 | HR = 3,030; p = 0,596 |
|  |  |  |  |  |  |
|  | ***Lung*** | HR = 1,121; p = 0,647 | HR = 1,055; p = 0,834 | HR = 1,334; p = 0,614 | HR = 1,553; p = 0,461 |
|  |  |  |  |  |  |
| R273 in C vs H | ***Head and Neck*** | **HR = 0,182; p = 0,056** | HR = 0,254; p = 0,336 | **HR = 0,080; p = 0,028** | **HR = 0,056; p = 0,049** |
|  |  |  |  |  |  |
|  | ***Oral*** | HR = 0,280; p = 0,171 | HR = 0,458; p = 0,541 | **HR = 0,112; p = 0,056** | **HR = 0,060; p = 0,076** |
|  |  |  |  |  |  |
|  | ***Oropharynx*** | N/A | N/A | N/A | N/A |
|  |  |  |  |  |  |
|  | ***Hypopharynx*** | N/A | N/A | N/A | N/A |
|  |  |  |  |  |  |
|  | ***Larinx*** | N/A | N/A | N/A | N/A |
|  |  |  |  |  |  |
|  | ***Oesophagus*** | HR = 1,803; p = 0,615 | N/A | N/A | N/A |
|  |  |  |  |  |  |
|  | ***Lung*** | N/A | N/A | N/A | N/A |
|  |  |  |  |  |  |
| R248 in Q vs W | ***Head and Neck*** | HR = 2,049; p = 0,409 | HR = 1,994; p = 0,662 | HR = 0,434; p = 0,498 | HR = 0,001; p = 0,611 |
|  |  |  |  |  |  |
|  | ***Oral*** | HR = 3,236; p = 0,301 | HR = 10,773; p = 0,503 | HR = 65,289; p = 0,610 | N/A |
|  |  |  |  |  |  |
|  | ***Oropharynx*** | N/A | N/A | N/A | N/A |
|  |  |  |  |  |  |
|  | ***Hypopharynx*** | N/A | N/A | N/A | N/A |
|  |  |  |  |  |  |
|  | ***Larinx*** | N/A | N/A | N/A | N/A |
|  |  |  |  |  |  |
|  | ***Oesophagus*** | N/A | N/A | N/A | N/A |
|  |  |  |  |  |  |
|  | ***Lung*** | N/A | N/A | N/A | N/A |
|  |  |  |  |  |  |
| Mutations in N-Term vs C-Term | ***Head and Neck*** | HR = 1,792; p = 0,197 | HR = 2,171; p = 0,105 | HR = 1,839; p = 0,448 | HR = 0,827; p = 0,838 |
|  |  |  |  |  |  |
|  | ***Oral*** | HR = 1,200; p = 0,743 | HR = 1,147; p = 0,816 | HR = 1,014; p = 0,988 | HR = 0,639; p = 0,687 |
|  |  |  |  |  |  |
|  | ***Oropharynx*** | N/A | N/A | N/A | N/A |
|  |  |  |  |  |  |
|  | ***Hypopharynx*** | N/A | N/A | N/A | N/A |
|  |  |  |  |  |  |
|  | ***Larinx*** | HR =2,439; p =0,307 | HR =2,439; p =0,307 | N/A | N/A |
|  |  |  |  |  |  |
|  | ***Oesophagus*** | HR = 1,200; p = 0,743 | N/A | N/A | N/A |
|  |  |  |  |  |  |
|  | ***Lung*** | HR = 0,956; p = 0,919 | HR = 1,134; p = 0,784 | HR = 1,300; p = 0,711 | HR = 1,023; p = 0,977 |
|  |  |  |  |  |  |
|  |  |  |  |  |  |
|  |  |  |  |  |  |
| Mutations in DBD vs N-Term | ***Head and Neck*** | HR = 0,618; p = 0,195 | HR = 0,781; p = 0,523 | HR = 0,587; p = 0,460 | HR = 0,758; p = 0,389 |
|  |  |  |  |  |  |
|  | ***Oral*** | HR = 0,985; p = 0,972 | HR = 1,134; p = 0,781 | HR = 0,911; p = 0,898 | HR = 0,987; p = 0,986 |
|  |  |  |  |  |  |
|  | ***Oropharynx*** | N/A | N/A | N/A | N/A |
|  |  |  |  |  |  |
|  | ***Hypopharynx*** | N/A | N/A | N/A | N/A |
|  |  |  |  |  |  |
|  | ***Larinx*** | **HR = 0,233; p = 0,054** | **HR = 0,223; p = 0,050** | HR = 0,042; p = 0,489 | N/A |
|  |  |  |  |  |  |
|  | ***Oesophagus*** | HR = 1,388; p = 0,583 | N/A | N/A | N/A |
|  |  |  |  |  |  |
|  | ***Lung*** | HR = 1,151; p = 0,657 | HR = 1,091; p = 0,785 | HR = 1,199; p = 0,734 | HR = 1,192; p = 0,742 |
|  |  |  |  |  |  |
| Mutations in DBD vs C-Term | ***Head and Neck*** | HR = 1,087; p = 0,744 | HR = 1,142; p = 0,610 | HR = 1,148; p = 0,734 | HR = 1,218; p = 0,638 |
|  |  |  |  |  |  |
|  | ***Oral*** | HR = 1,122; p = 0,734 | HR = 1,293; p = 0,461 | HR = 1,064; p = 0,908 | HR = 1,368; p = 0,565 |
|  |  |  |  |  |  |
|  | ***Oropharynx*** | HR = 1,865; p = 0,339 | HR = 1,516; p = 0,632 | N/A | N/A |
|  |  |  |  |  |  |
|  | ***Hypopharynx*** | N/A | N/A | N/A | N/A |
|  |  |  |  |  |  |
|  | ***Larinx*** | HR =0,659; p =0,443 | HR = 0,818; p = 0,714 | HR = 0,566; p = 0,587 | HR = 0,319; p = 0,287 |
|  |  |  |  |  |  |
|  | ***Oesophagus*** | HR = 0,511; p = 0,261 | **HR = 0,064; p = 0,027** | HR = 0,390; p = 0,360 | N/A |
|  |  |  |  |  |  |
|  | ***Lung*** | HR = 1,042; p = 0,902 | HR = 1,083; p = 0,821 | HR = 1,526; p = 0,429 | HR = 1,354; p = 0,621 |
|  |  |  |  |  |  |
| G245 vs other mutations | ***Head and Neck*** | HR = 0,231; p = 0,145 | HR = 0,300; p = 0,233 | HR = 0,047; p = 0,329 | HR = 0,00; p = 0,970 |
|  |  |  |  |  |  |
|  | ***Oral*** | N/A | N/A | N/A | N/A |
|  |  |  |  |  |  |
|  | ***Oropharynx*** | N/A | N/A | N/A | N/A |
|  |  |  |  |  |  |
|  | ***Hypopharynx*** | N/A | N/A | N/A | N/A |
|  |  |  |  |  |  |
|  | ***Larinx*** | HR =0,583; p =0,596 | HR =1,367; p =0,767 | N/A | N/A |
|  |  |  |  |  |  |
|  | ***Oesophagus*** | HR = 0,386; p = 0,195 | N/A | N/A | N/A |
|  |  |  |  |  |  |
|  | ***Lung*** | HR = 1,235; p = 0,678 | HR = 1,383; p = 0,531 | HR = 0,728; p = 0,662 | HR = 0,705; p = 0,636 |
|  |  |  |  |  |  |
| R248 vs other mutations | ***Head and Neck*** | HR = 1,070; p = 0,873 | HR = 1,156; p = 0,730 | HR = 0,620; p = 0,421 | HR = 1,021; p = 0,977 |
|  |  |  |  |  |  |
|  | ***Oral*** | HR = 1,150; p = 0,785 | HR = 1,096; p = 0,859 | HR = 1,628; p = 0,631 | HR = 1,893; p = 0,535 |
|  |  |  |  |  |  |
|  | ***Oropharynx*** | HR = 0,502; p = 0,391 | HR = 0,924; p = 0,935 | HR = 0,407; p = 0,422 | HR = 0,105; p = 0,323 |
|  |  |  |  |  |  |
|  | ***Hypopharynx*** | N/A | N/A | N/A | N/A |
|  |  |  |  |  |  |
|  | ***Larinx*** | N/A | N/A | N/A | N/A |
|  |  |  |  |  |  |
|  | ***Oesophagus*** | HR = 0,653; p = 0,557 | HR = 0,270; p = 0,253 | HR = 0,649; p = 0,681 | HR = 0,330; p = 0,316 |
|  |  |  |  |  |  |
|  | ***Lung*** | HR = 0,596; p = 0,310 | HR = 0,676; p = 0,443 | **HR = 0,152; p = 0,011** | **HR = 0,109 p = 0,005** |
|  |  |  |  |  |  |
| R282 vs other mutations | ***Head and Neck*** | HR = 0,269; p = 0,191 | HR = 0,218; p = 0,131 | HR = 0,649; p = 0,669 | HR = 0,501; p = 0,502 |
|  |  |  |  |  |  |
|  | ***Oral*** | HR = 2,390; p = 0,785 | HR = 2,857; p = 0,301 | HR = 1,019; p = 0,985 | HR = 1,164; p = 0,885 |
|  |  |  |  |  |  |
|  | ***Oropharynx*** | N/A | N/A | N/A | N/A |
|  |  |  |  |  |  |
|  | ***Hypopharynx*** | N/A | N/A | N/A | N/A |
|  |  |  |  |  |  |
|  | ***Larinx*** | HR =0,047; p =0,515 | N/A | N/A | N/A |
|  |  |  |  |  |  |
|  | ***Oesophagus*** | HR = 0,553; p = 0,324 | **HR = 0,089; p = 0,049** | N/A | N/A |
|  |  |  |  |  |  |
|  | ***Lung*** | HR = 0,871; p = 0,813 | HR = 0,972; p = 0,961 | HR = 0,942; p = 0,953 | HR =1,010; p = 0,993 |
|  |  |  |  |  |  |
| R175 vs other mutations | ***Head and Neck*** | **HR = 0,228; p = 0,004** | **HR = 0,146; p = 0,008** | HR = 20,346; p = 0,758 | HR = 11318,19; p = 0,980 |
|  |  |  |  |  |  |
|  | ***Oral*** | HR = 0,381; p = 0,180 | HR = 5,814; p = 0,089 | N/A | N/A |
|  |  |  |  |  |  |
|  | ***Oropharynx*** | N/A | N/A | N/A | N/A |
|  |  |  |  |  |  |
|  | ***Hypopharynx*** | N/A | N/A | N/A | N/A |
|  |  |  |  |  |  |
|  | ***Larinx*** | **HR = 0,100; p = 0,033** | **HR = 0,072; p = 0,022** | N/A | N/A |
|  |  |  |  |  |  |
|  | ***Oesophagus*** | HR = 1,530; p = 0,558 | N/A | HR = 1,277; p = 0,751 | HR = 1,381; p = 0,711 |
|  |  |  |  |  |  |
|  | ***Lung*** | HR = 0,608; p = 0,329 | HR = 0,469; p = 0,147 | N/A | N/A |
|  |  |  |  |  |  |
| H179 vs other mutations | ***Head and Neck*** | HR = 3,720; p = 0,191 | HR = 2,061; p = 0,473 | HR = 0,390; p = 0,070 | HR = 0,303; p = 0,118 |
|  |  |  |  |  |  |
|  | ***Oral*** | N/A | HR = 0,072; p = 0,973 | HR = 0,500; p = 0,250 | HR = 0,729; p = 0,762 |
|  |  |  |  |  |  |
|  | ***Oropharynx*** | N/A | N/A | N/A | N/A |
|  |  |  |  |  |  |
|  | ***Hypopharynx*** | N/A | N/A | N/A | N/A |
|  |  |  |  |  |  |
|  | ***Larinx*** | HR =0,048; p =0,602 | N/A | N/A | N/A |
|  |  |  |  |  |  |
|  | ***Oesophagus*** | N/A | N/A | N/A | N/A |
|  |  |  |  |  |  |
|  | ***Lung*** | HR =1,681; p =0,374 | HR =2,399; p =0,142 | HR =0,722; p =0,654 | HR =1,088; p =0,791 |
|  |  |  |  |  |  |
| H193 vs other mutations | ***Head and Neck*** | **HR = 0,420; p = 0,058** | **HR = 0.302,; p = 0,015** | HR = 1,050; p = 0,962 | HR = 0,949; p = 0,961 |
|  |  |  |  |  |  |
|  | ***Oral*** | HR = 0,824; p = 0,787 | HR = 1,689; p = 0,483 | HR = 0,793; p = 0,819 | HR = 0,454; p = 0,471 |
|  |  |  |  |  |  |
|  | ***Oropharynx*** | **HR = 0,048; p = 0,014** | **HR = 0,002; p = 0,006** | HR = 23,350; p = 0,643 | N/A |
|  |  |  |  |  |  |
|  | ***Hypopharynx*** | N/A | N/A | N/A | N/A |
|  |  |  |  |  |  |
|  | ***Larinx*** | N/A | N/A | N/A | N/A |
|  |  |  |  |  |  |
|  | ***Oesophagus*** | N/A | N/A | N/A | N/A |
|  |  |  |  |  |  |
|  | ***Lung*** | HR = 2,059; p = 0,472 | HR = 1,892; p = 0,526 | N/A | N/A |
|  |  |  |  |  |  |
| R196 vs other mutations | ***Head and Neck*** | HR = 0,638; p = 0,442 | HR = 0,461; p = 0,195 | HR = 1,205; p = 0,796 | HR = 1,058; p = 0,858 |
|  |  |  |  |  |  |
|  | ***Oral*** | HR = 1,503; p = 0,489 | HR = 1,984; p = 0,255 | HR = 1,132; p = 0,865 | HR = 1,432; p = 0,634 |
|  |  |  |  |  |  |
|  | ***Oropharynx*** | N/A | N/A | N/A | N/A |
|  |  |  |  |  |  |
|  | ***Hypopharynx*** | N/A | N/A | N/A | N/A |
|  |  |  |  |  |  |
|  | ***Larinx*** | N/A | N/A | N/A | N/A |
|  |  |  |  |  |  |
|  | ***Oesophagus*** | N/A | N/A | N/A | N/A |
|  |  |  |  |  |  |
|  | ***Lung*** | N/A | N/A | N/A | N/A |
|  |  |  |  |  |  |
| R213 vs other mutations | ***Head and Neck*** | **HR = 0,371; p = 0,030** | HR = 0,438; p = 0,108 | HR = 0,993; p = 0,994 | HR = 1,041; p = 0,969 |
|  |  |  |  |  |  |
|  | ***Oral*** | **HR = 0,399; p = 0,075** | HR = 2,096; p = 0,153 | N/A | N/A |
|  |  |  |  |  |  |
|  | ***Oropharynx*** | N/A | N/A | N/A | N/A |
|  |  |  |  |  |  |
|  | ***Hypopharynx*** | N/A | N/A | N/A | N/A |
|  |  |  |  |  |  |
|  | ***Larinx*** | N/A | N/A | N/A | N/A |
|  |  |  |  |  |  |
|  | ***Oesophagus*** | HR = 0,447; p = 0,183 | HR = 0,419; p = 0,439 | N/A | N/A |
|  |  |  |  |  |  |
|  | ***Lung*** | **HR = 0,234; p = 0,014** | **HR = 0,316; p = 0,053** | N/A | N/A |
|  |  |  |  |  |  |
| R273 vs other mutations | ***Head and Neck*** | HR = 1,387; p = 0,403 | HR = 0,707; p = 0,305 | HR = 0,723; p = 0,455 | HR = 0,696; p = 0,407 |
|  |  |  |  |  |  |
|  | ***Oral*** | HR = 1,422; p = 0,448 | HR = 0,720; p = 0,483 | HR = 0,631; p = 0,343 | HR = 0,566; p = 0,244 |
|  |  |  |  |  |  |
|  | ***Oropharynx*** | N/A | N/A | N/A | N/A |
|  |  |  |  |  |  |
|  | ***Hypopharynx*** | N/A | N/A | N/A | N/A |
|  |  |  |  |  |  |
|  | ***Larinx*** | HR = 0,988; p = 0,987 | HR = 1,280; p = 0,750 | N/A | N/A |
|  |  |  |  |  |  |
|  | ***Oesophagus*** | HR =1,034; p = 0,948 | HR =1,663; p = 0,647 | N/A | N/A |
|  |  |  |  |  |  |
|  | ***Lung*** | HR = 0,808; p = 0,610 | HR = 1,121; p = 0,803 | HR = 1,081; p = 0,939 | HR = 1,247; p = 0,829 |
|  |  |  |  |  |  |
| Wild Type vs Missense | ***Head and Neck*** | **HR = 1,601; p = 0,016** | **HR = 1,688; p = 0,011** | HR = 1,192; p = 0,545 | HR = 0,778; p = 0,439 |
|  |  |  |  |  |  |
|  | ***Oral*** | HR = 1,009; p = 0,969 | HR = 1,157; p = 0,964 | HR = 0,921; p = 0,815 | HR = 0,953; p = 0,897 |
|  |  |  |  |  |  |
|  | ***Oropharynx*** | **HR = 7,417; p = 0,002** | **HR = 13,484; p = 0,001** | **HR = 11,353; p = 0,007** | **HR = 51,970; p = 0,033** |
|  |  |  |  |  |  |
|  | ***Hypopharynx*** | HR = 3,464; p = 0,381 | N/A | N/A | N/A |
|  |  |  |  |  |  |
|  | ***Larinx*** | **HR = 6,943; p = 0,013** | **HR = 11,845; p = 0,025** | HR = 1,071; p = 0,936 | HR = 0,731; p = 0,802 |
|  |  |  |  |  |  |
|  | ***Oesophagus*** | HR = 1,199; p = 0,647 | HR = 4,083; p = 0,261 | HR = 1,029; p = 0,970 | HR = 0,860; p = 0,868 |
|  |  |  |  |  |  |
|  | ***Lung*** | **HR = 0,659; p = 0,037** | **HR = 0,647; p = 0,031** | HR = 0,850; p = 0,688 | HR = 0,833; p = 0,652 |
|  |  |  |  |  |  |
| Wild Type vs Frameshift | ***Head and Neck*** | HR = 1,323; p = 0,303 | HR = 1,346; p = 0,314 | HR = 1,427; p = 0,356 | HR = 1,455; p = 0,375 |
|  |  |  |  |  |  |
|  | ***Oral*** | HR = 1,039; p = 0,915 | HR = 0,791; p = 0,534 | HR = 1,088; p = 0,866 | HR = 1,117; p = 0,834 |
|  |  |  |  |  |  |
|  | ***Oropharynx*** | **HR = 8,221; p = 0,006** | HR = 5,872; p = 0,103 | **HR = 32,637; p = 0,005** | **HR = 20,814; p = 0,050** |
|  |  |  |  |  |  |
|  | ***Hypopharynx*** | HR = 1,732; p = 0,698 | N/A | HR = 1,225; p = 0,887 | N/A |
|  |  |  |  |  |  |
|  | ***Larinx*** | HR = 1,495; p = 0,556 | HR = 1,183; p = 0,875 | HR = 0,400; p = 0,455 | HR = 0,979; p = 0,989 |
|  |  |  |  |  |  |
|  | ***Oesophagus*** | HR = 2,208; p = 0,104 | HR = 3,749; p = 0,416 | N/A | N/A |
|  |  |  |  |  |  |
|  | ***Lung*** | HR = 0,748; p = 0,300 | HR = 0,4684; p = 0,190 | HR = 0,788; p = 0,660 | HR = 0,646; p = 0,448 |
|  |  |  |  |  |  |
| Wild Type vs Inframe | ***Head and Neck*** | HR = 1,683; p = 0,322 | HR = 2,073; p = 0,189 | HR = 0,744; p = 0,775 | HR = 0,799; p = 0,836 |
|  |  |  |  |  |  |
|  | ***Oral*** | HR = 1,403; p = 0,580 | HR = 1,921; p = 0,326 | HR = 0,044; p = 0,520 | HR = 0,000; p = 0,986 |
|  |  |  |  |  |  |
|  | ***Oropharynx*** | HR = 0,046; p = 0,760 | HR = 0,001; p = 0,994 | HR = 0,042; p = 0,762 | N/A |
|  |  |  |  |  |  |
|  | ***Hypopharynx*** | N/A | N/A | N/A | N/A |
|  |  |  |  |  |  |
|  | ***Larinx*** | N/A | N/A | HR = 0,400; p = 0,455 | N/A |
|  |  |  |  |  |  |
|  | ***Oesophagus*** | HR = 0,043; p = 0,577 | N/A | HR = 1,137; p = 0,917 | N/A |
|  |  |  |  |  |  |
|  | ***Lung*** | HR = 0,434; p = 0,176 | HR = 0,544; p = 0,337 | HR = 1,096; p = 0,931 | HR = 1,267; p = 0,831 |
|  |  |  |  |  |  |
| Wild Type vs Splice | ***Head and Neck*** | HR = 1,208; p = 0,580 | HR = 0,935; p = 0,866 | HR = 1,107; p = 0,839 | HR = 0,914; p = 0,871 |
|  |  |  |  |  |  |
|  | ***Oral*** | HR = 1,024; p = 0,959 | HR = 1,105; p = 0,830 | HR = 0,641; p = 0,558 | HR = 0,790; p = 0,769 |
|  |  |  |  |  |  |
|  | ***Oropharynx*** | **HR = 8,784; p = 0,052** | HR = 1,577; p = 0,836 | N/A | N/A |
|  |  |  |  |  |  |
|  | ***Hypopharynx*** | N/A | N/A | N/A | N/A |
|  |  |  |  |  |  |
|  | ***Larinx*** | HR = 1,199; p = 0,837 | HR = 0,023; p = 0,398 | HR = 2,253; p = 0,484 | N/A |
|  |  |  |  |  |  |
|  | ***Oesophagus*** | HR = 1,553; p = 0,487 | N/A | HR = 0,532; p = 0,530 | HR = 0,283; p = 0,687 |
|  |  |  |  |  |  |
|  | ***Lung*** | HR = 0,530; p = 0,069 | **HR = 0,486; p = 0,041** | HR = 1,931; p = 0,176 | HR = 1,634; p = 0,364 |
|  |  |  |  |  |  |
| Wild Type vs Stop | ***Head and Neck*** | **HR = 2,098; p = 0,003** | **HR = 2,016; p = 0,006** | HR = 0,847; p = 0,710 | HR = 0,840; p = 0,707 |
|  |  |  |  |  |  |
|  | ***Oral*** | HR = 1,587; p = 0,118 | HR = 1,505; p = 0,168 | HR = 0,710; p = 0,521 | HR = 0,625; p = 0,384 |
|  |  |  |  |  |  |
|  | ***Oropharynx*** | HR = 3,060; p = 0,310 | HR = 2,169; p = 0,558 | N/A | N/A |
|  |  |  |  |  |  |
|  | ***Hypopharynx*** | HR =0,026; p = 0,681 | HR =0,000; p = 0,866 | HR =1,225; p = 0,887 | HR =0,000; p = 0,539 |
|  |  |  |  |  |  |
|  | ***Larinx*** | **HR = 4,145; p = 0,05** | HR = 2,680; p = 0,405 | HR = 1,366; p = 0,825 | HR = 1,030; p = 0,991 |
|  |  |  |  |  |  |
|  | ***Oesophagus*** | HR = 0,639; p = 0,387 | HR = 0,006; p = 0,210 | HR = 0,763; p = 0,771 | N/A |
|  |  |  |  |  |  |
|  | ***Lung*** | HR =0,757; p = 0,319 | HR =0,759; p = 0,337 | HR =1,411; p = 0,491 | HR = 1,573; p = 0,407 |
|  |  |  |  |  |  |
| Missense vs Inframe | ***Head and Neck*** | HR = 0,987; p = 0,980 | HR = 0,860; p = 0,780 | HR = 0,507; p = 0,504 | HR = 1,074; p = 0,946 |
|  |  |  |  |  |  |
|  | ***Oral*** | HR = 1,338; p = 0,627 | HR = 0,877; p = 0,839 | HR = 0,046; p = 0,507 | HR = 0,000; p = 0,987 |
|  |  |  |  |  |  |
|  | ***Oropharynx*** | HR = 0,030; p = 0,388 | HR = 0,001; p = 0,989 | N/A | N/A |
|  |  |  |  |  |  |
|  | ***Hypopharynx*** | N/A | N/A | N/A | N/A |
|  |  |  |  |  |  |
|  | ***Larinx*** | N/A | N/A | N/A | N/A |
|  |  |  |  |  |  |
|  | ***Oesophagus*** | HR = 0,048; p = 0,528 | N/A | HR = 1,186; p = 0,870 | HR = 0,885; p = 0,908 |
|  |  |  |  |  |  |
|  | ***Lung*** | HR = 0,542; p = 0,301 | HR = 0,572; p = 0,352 | HR = 1,026; p = 0,980 | HR = 1,181; p = 0,873 |
|  |  |  |  |  |  |
| Missense vs Frameshift | ***Head and Neck*** | HR = 0,804; p = 0,392 | HR = 0,912; p = 0,731 | HR = 1,081; p = 0,824 | HR = 1,185 p = 0,876 |
|  |  |  |  |  |  |
|  | ***Oral*** | HR = 0,917; p = 0,800 | HR = 0,877; p = 0,839 | HR = 1,065; p = 0,892 | HR = 1,052 p = 0,919 |
|  |  |  |  |  |  |
|  | ***Oropharynx*** | HR = 0,937; p = 0,927 | HR = 1,911; p = 0,537 | HR = 4,213; p = 0,154 | N/A |
|  |  |  |  |  |  |
|  | ***Hypopharynx*** | HR = 0,707; p = 0,809 | N/A | N/A | N/A |
|  |  |  |  |  |  |
|  | ***Larinx*** | HR = 0,404; p = 0,104 | **HR = 0,236; p = 0,031** | HR = 0,459; p = 0,478 | HR = 0,196; p = 0,183 |
|  |  |  |  |  |  |
|  | ***Oesophagus*** | HR = 1,933; p = 0,559 | **HR = 5,759; p = 0,037** | HR = 0,038; p = 0,337 | N/A |
|  |  |  |  |  |  |
|  | ***Lung*** | HR = 1,153; p = 0,582 | HR = 1,039; p = 0,877 | HR = 1,048; p = 0,919 | HR = 0,987; p = 0,977 |
|  |  |  |  |  |  |
| Missense vs Splice | ***Head and Neck*** | HR = 0,827; p = 0,559 | HR = 0,721; p = 0,368 | HR = 0,922; p = 0,865 | HR = 0,817; p = 0,690 |
|  |  |  |  |  |  |
|  | ***Oral*** | HR = 1,234; p = 0,610 | HR = 1,292; p = 0,553 | HR = 0,750; p = 0,697 | HR = 0,592; p = 0,503 |
|  |  |  |  |  |  |
|  | ***Oropharynx*** | HR = 1,283; p = 0,821 | HR = 3,334; p = 0,685 | N/A | N/A |
|  |  |  |  |  |  |
|  | ***Hypopharynx*** | N/A | N/A | N/A | N/A |
|  |  |  |  |  |  |
|  | ***Larinx*** | HR = 0,278; p = 0,087 | HR = 0,168; p = 0,092 | HR = 1,827; p = 0,418 | HR = 1,117; p = 0,913 |
|  |  |  |  |  |  |
|  | ***Oesophagus*** | HR = 1,519; p = 0,394 | HR = 1,592; p = 0,525 | HR = 0,622; p = 0,534 | HR = 0,295; p = 0,250 |
|  |  |  |  |  |  |
|  | ***Lung*** | HR = 0,794; p = 0,469 | HR = 0,750; p = 0,367 | **HR = 2,519; p = 0,018** | **HR = 2,355; p = 0,034** |
|  |  |  |  |  |  |
| Missense vs Stop | ***Head and Neck*** | HR = 1,263; p = 0,298 | HR = 1,159; p = 0,519 | HR = 0,650; p = 0,302 | HR = 0,622; p = 0,265 |
|  |  |  |  |  |  |
|  | ***Oral*** | HR = 1,528; p = 0,121 | HR = 1,387; p = 0,240 | HR = 0,650; p = 0,469 | HR = 0,675; p = 0,437 |
|  |  |  |  |  |  |
|  | ***Oropharynx*** | HR = 0,033; p = 0,418 | HR = 0,001; p = 0,963 | HR = 0,024; p = 0,418 | HR = 0,006; p = 0,265 |
|  |  |  |  |  |  |
|  | ***Hypopharynx*** | HR = 0,015; p = 0,610 | N/A | N/A | N/A |
|  |  |  |  |  |  |
|  | ***Larinx*** | HR = 0,822; p = 0,699 | HR = 0,673; p = 0,539 | HR = 0,842; p = 0,876 | HR = 0,020; p = 0,147 |
|  |  |  |  |  |  |
|  | ***Oesophagus*** | HR = 0,514; p = 0,113 | HR = 0,237; p = 0,078 | HR = 0,716; p = 0,244 | HR = 0,793; p = 0,745 |
|  |  |  |  |  |  |
|  | ***Lung*** | HR = 1,073; p = 0,770 | HR = 1,058; p = 0,822 | HR = 1,604; p = 0,669 | HR = 1,586; p = 0,287 |
|  |  |  |  |  |  |
| Frameshift vs Inframe | ***Head and Neck*** | HR = 1,248; p = 0,688 | HR = 0,860; p = 0,780 | HR = 0,456; p = 0,455 | HR = 0,475; p = 0,535 |
|  |  |  |  |  |  |
|  | ***Oral*** | HR = 1,193; p = 0,794 | HR = 2,584; p = 0,477 | HR = 0,039; p = 0,511 | N/A |
|  |  |  |  |  |  |
|  | ***Oropharynx*** | HR = 0,018; p = 0,403 | HR = 0,002; p = 0,365 | N/A | N/A |
|  |  |  |  |  |  |
|  | ***Hypopharynx*** | N/A | N/A | N/A | N/A |
|  |  |  |  |  |  |
|  | ***Larinx*** | N/A | HR = 8,842; p = 0,186 | N/A | N/A |
|  |  |  |  |  |  |
|  | ***Oesophagus*** | HR = 0,037; p = 0,393 | N/A | N/A | N/A |
|  |  |  |  |  |  |
|  | ***Lung*** | HR = 0,544; p = 0,362 | HR = 0,389; p = 0,191 | HR = 1,551; p = 0,686 | HR = 0,820; p = 0,816 |
|  |  |  |  |  |  |
| Frameshift vs Splice | ***Head and Neck*** | HR = 0,979; p = 0,957 | HR = 0,793; p = 0,587 | HR = 0,820; p = 0,714 | HR = 0,940; p = 0,913 |
|  |  |  |  |  |  |
|  | ***Oral*** | HR = 1,340; p = 0,566 | HR = 1,539; p = 0,422 | HR = 0,662; p = 0,614 | HR = 0,775; p = 0,788 |
|  |  |  |  |  |  |
|  | ***Oropharynx*** | HR = 2,828; p = 0,469 | N/A | N/A | N/A |
|  |  |  |  |  |  |
|  | ***Hypopharynx*** | N/A | N/A | N/A | N/A |
|  |  |  |  |  |  |
|  | ***Larinx*** | HR = 0,597; p = 0,537 | HR = 3,894; p = 0,240 | HR = 0,177; p = 0,291 | HR = 2,555; p = 0,449 |
|  |  |  |  |  |  |
|  | ***Oesophagus*** | HR = 0,626; p = 0,444 | N/A | N/A | N/A |
|  |  |  |  |  |  |
|  | ***Lung*** | HR = 0,637; p = 0,243 | HR = 0,699; p = 0,367 | HR = 2,239; p = 0,126 | HR = 2,547; p = 0,080 |
|  |  |  |  |  |  |
| Frameshift vs Stop | ***Head and Neck*** | HR = 1,572; p = 0,123 | HR = 1,378; p = 0,307 | HR = 0,569; p = 0,246 | HR = 0,382; p = 0,070 |
|  |  |  |  |  |  |
|  | ***Oral*** | HR = 1,667; p = 0,175 | HR = 1,534; p = 0,287 | HR = 0,628; p = 0,448 | HR = 0,375; p = 0,173 |
|  |  |  |  |  |  |
|  | ***Oropharynx*** | HR = 0,021; p = 0,435 | HR = 0,001; p = 0,586 | HR = 0,010; p = 0,439 | HR = 0,017; p = 0,536 |
|  |  |  |  |  |  |
|  | ***Hypopharynx*** | HR = 0,026; p = 0,681 | N/A | HR = 1,414; p = 0,809 | N/A |
|  |  |  |  |  |  |
|  | ***Larinx*** | HR = 2,229; p = 0,216 | HR = 2,545; p = 0,237 | HR = 1,633; p = 0,730 | HR = 0,00; p = 0,708 |
|  |  |  |  |  |  |
|  | ***Oesophagus*** | **HR = 0,255; p = 0,010** | N/A | N/A | N/A |
|  |  |  |  |  |  |
|  | ***Lung*** | HR = 1,061; p = 0,849 | HR = 1,250; p = 0,488 | HR = 1,732; p = 0,309 | HR = 1,595; p = 0,409 |
|  |  |  |  |  |  |
| Inframe vs Splice | ***Head and Neck*** | HR = 0,812; p = 0,726 | HR = 0,672; p = 0,574 | HR = 1,897; p = 0,559 | HR = 0,335; p = 0,449 |
|  |  |  |  |  |  |
|  | ***Oral*** | HR = 0,893; p = 0,875 | HR = 0,642; p = 0,644 | HR = 33,002; p = 0,607 | HR = 2,473; p = 0,996 |
|  |  |  |  |  |  |
|  | ***Oropharynx*** | N/A | N/A | N/A | N/A |
|  |  |  |  |  |  |
|  | ***Hypopharynx*** | N/A | N/A | N/A | N/A |
|  |  |  |  |  |  |
|  | ***Larinx*** | N/A | N/A | N/A | N/A |
|  |  |  |  |  |  |
|  | ***Oesophagus*** | HR = 26,667; p = 0,571 | N/A | HR = 0,452; p = 0,521 | N/A |
|  |  |  |  |  |  |
|  | ***Lung*** | N/A | N/A | N/A | N/A |
|  |  |  |  |  |  |
| Inframe vs Stop | ***Head and Neck*** | HR = 1,104; p = 0,854 | HR = 0,956; p = 0,939 | HR = 1,325; p = 0,793 | HR = 0,598; p = 0,670 |
|  |  |  |  |  |  |
|  | ***Oral*** | HR = 0,989; p = 0,985 | HR = 1,056; p = 0,939 | HR = 24,776; p = 0,593 | N/A |
|  |  |  |  |  |  |
|  | ***Oropharynx*** | N/A | N/A | N/A | N/A |
|  |  |  |  |  |  |
|  | ***Hypopharynx*** | N/A | N/A | N/A | N/A |
|  |  |  |  |  |  |
|  | ***Larinx*** | N/A | N/A | N/A | N/A |
|  |  |  |  |  |  |
|  | ***Oesophagus*** | HR = 22,507; p = 0,657 | N/A | HR = 1,149; p = 0,909 | N/A |
|  |  |  |  |  |  |
|  | ***Lung*** | N/A | N/A | N/A | N/A |
|  |  |  |  |  |  |
| Splice vs Stop | ***Head and Neck*** | HR = 1,694; p = 0,156 | HR = 1,896; p = 0,121 | HR = 0,721; p = 0,577 | HR = 0,609; p = 0,436 |
|  |  |  |  |  |  |
|  | ***Oral*** | HR = 1,357; p = 0,514 | HR = 1,208; p = 0,695 | HR = 0,988; p = 0,988 | HR = 0,534; p = 0,506 |
|  |  |  |  |  |  |
|  | ***Oropharynx*** | N/A | N/A | N/A | N/A |
|  |  |  |  |  |  |
|  | ***Hypopharynx*** | N/A | N/A | N/A | N/A |
|  |  |  |  |  |  |
|  | ***Larinx*** | HR = 3,065; p = 0,171 | HR = 5,711; p = 0,174 | HR = 0,444; p = 0,484 | HR = 0,00; p = 0,927 |
|  |  |  |  |  |  |
|  | ***Oesophagus*** | HR = 2,112; p = 0,244 | N/A | HR = 0,424; p = 0,484 | HR = 0,674; p = 0,819 |
|  |  |  |  |  |  |
|  | ***Lung*** | HR = 1,445; p = 0,326 | HR = 1,549; p = 0,257 | HR = 0,699; p = 0,462 | HR = 0,674; p = 0,481 |
|  |  |  |  |  |  |
| V157 vs other mutations | ***Head and Neck*** | N/A | N/A | N/A | N/A |
|  |  |  |  |  |  |
|  | ***Oral*** | N/A | N/A | N/A | N/A |
|  |  |  |  |  |  |
|  | ***Oropharynx*** | N/A | N/A | N/A | N/A |
|  |  |  |  |  |  |
|  | ***Hypopharynx*** | N/A | N/A | N/A | N/A |
|  |  |  |  |  |  |
|  | ***Larinx*** | N/A | N/A | N/A | N/A |
|  |  |  |  |  |  |
|  | ***Oesophagus*** | N/A | N/A | N/A | N/A |
|  |  |  |  |  |  |
|  | ***Lung*** | HR = 0,824; p = 0,644 | HR = 0,837; p = 0,671 | HR = 0,226; p = 0,006 | HR = 0,212 p = 0,004 |
|  |  |  |  |  |  |
| R158 vs other mutations | ***Head and Neck*** | N/A | N/A | N/A | N/A |
|  |  |  |  |  |  |
|  | ***Oral*** | N/A | N/A | N/A | N/A |
|  |  |  |  |  |  |
|  | ***Oropharynx*** | N/A | N/A | N/A | N/A |
|  |  |  |  |  |  |
|  | ***Hypopharynx*** | N/A | N/A | N/A | N/A |
|  |  |  |  |  |  |
|  | ***Larinx*** | N/A | N/A | N/A | N/A |
|  |  |  |  |  |  |
|  | ***Oesophagus*** | N/A | N/A | N/A | N/A |
|  |  |  |  |  |  |
|  | ***Lung*** | HR = 0,739; p = 0,508 | HR = 0,715; p = 0,468 | HR = 0,913; p = 0,900 | HR = 0,963; p = 0,958 |
|  |  |  |  |  |  |
| P278 vs other mutations | ***Head and Neck*** | N/A | N/A | N/A | N/A |
|  |  |  |  |  |  |
|  | ***Oral*** | N/A | N/A | N/A | N/A |
|  |  |  |  |  |  |
|  | ***Oropharynx*** | N/A | N/A | N/A | N/A |
|  |  |  |  |  |  |
|  | ***Hypopharynx*** | N/A | N/A | N/A | N/A |
|  |  |  |  |  |  |
|  | ***Larinx*** | N/A | N/A | N/A | N/A |
|  |  |  |  |  |  |
|  | ***Oesophagus*** | N/A | N/A | N/A | N/A |
|  |  |  |  |  |  |
|  | ***Lung*** | HR = 1,795; p = 0,317 | HR = 1,324; p = 0,634 | HR = 1,163; p = 0,836 | HR = 1,072; p = 0,925 |
|  |  |  |  |  |  |

Supplemental material Table 3:

1. Gene ontology and network analysis for wild-type TP53 in OSCC;
2. Gene ontology and network analysis for mutated TP53 in OSCC;
3. Gene ontology and network analysis for wild-type TP53 in OP;
4. Gene ontology and network analysis for mutated TP53 in OP;
5. Gene ontology and network analysis for wild-type TP53 in L;
6. Gene ontology and network analysis for mutated TP53 in L;
7. Gene ontology and network analysis for HPV negative TP53 in OP;
8. Gene ontology and network analysis for HPV positive TP53 in OP;
9. Gene ontology and network analysis for wild-type TP53 in HP;
10. Gene ontology and network analysis for mutated TP53 in HP.

“Not reported” means the reported gene was not included in any Gene ontology result.

A

| **Wild-type TP53 in OSCC** | | | |
| --- | --- | --- | --- |
| **Gene** | **Protein** | **Frequency and alteration type** | **Cell function** |
| CDKN2A | P16^INK4A^ | **47.1%**  40% Homozygousdeletion  1.4% mRNA upregulation  5.7% mutation | Cell aging; regulation of establishment of protein localization to mitochondrion; positive regulation of cellular protein localization. |
| DROSHA | Ribonuclease III | **25.7%**  22.9% mRNA upregulation  2.9% mRNA downregulation | Not reported |
| TP63 | Tumor protein 63 | **20.0%**  8.6% Amplification  12.9% mRNA upregulation  2.9% mutation | Cell aging; intrinsic apoptotic signaling patway in response to DNA damage; regulation of establishment of protein localization to mitochondrion; positive regulation of cellular protein localization. |
| PMS2 | Mismatch repair endonuclease PMS2 | **18.6%**  1.4% Amplification  15.7% mRNA upregulation  4.3% mutation | Cellular response to DNA damage stimulus. |
| CDK9 | Cyclin-dependent kinase 9 | **17.1%**  17.1% Amplification  1.4% mRNA upregulation | Cellular response to DNA damage stimulus. |
| DDB2 | DNA  damage-binding protein 2 | **17.1%**  4.3% Amplifiation  11.4% mRNA upregulation  1.4% mutation | Cellular response to DNA damage stimulus. |
| EPHA2 | Ephrin type-A receptor 2 | **17.1%**  1.4% Homozygous deletion  8.6% mRNA upregulation  8.6% mutation | Intrinsic apoptotic signaling patway in response to DNA damage; positive regulation of cellular protein localization. |

B

| **Mutated TP53 in OSCC** | | | |
| --- | --- | --- | --- |
| **Gene** | **Protein** | **Frequency and alteration type** | **Cell function** |
| CDKN2A | P16^INK4A^ | **63.1%**  34.7% Homozygous deletion  29.5% mutation | Regulation of protein export from nucleus; regulation of signal transduction by p53 class mediator; regulation of cell cycle G1/S phase transition; negative regulation of cell cycle process; |
| TP63 | Tumor protein 63 | **35.2%**  17.6% Amplification  20.5% mRNA upregulation  0.6% mRNA downregulation  3.4% mutation | Negative regulation of cellular senescence; Negative regulation of cell aging; positive regulation of mitochondrial outer membrane permeabilization involved in apoptotic signaling pathway; regulation of signal transduction by p53 class mediator; regulation of cell cycle G1/S phase transition; |
| NDRG1 | Protein NDRG1 | **25.6%**  9.7% Amplification  19.9% mRNA upregulation | Cellular response to DNA damage stimulus |
| GSK3B | Glycogen synthase kinase-3 beta | **25.6%**  3.4% Amplification  22.2% mRNA upregulation  0.6% mRNA downregulation | Positive regulation of mitochondrial outer membrane permeabilization involved in apoptotic signaling pathway; regulation of protein export from nucleus; negative regulation of apoptotic process |
| SNAI2 | Snail family transcriptional repressor 2 | **23.8%**  9.7% Amplification  17.6% mRNA upregulation | Regulation of signal transduction by p53 class mediator; negative regulation of apoptotic process; |
| BCL6 | B-cell lymphoma 6 protein | **23.3%**  16.5% Amplification  10.2% mRNA upregulation | Negative regulation of cell aging; negative regulation of cell cycle process; cellular response to DNA damage stimulus; negative regulation of apoptotic process; |
| CCNK | Cycline K | **22.7%**  2.3% Amplification  0.6% Homozygous deletion  18.2% mRNA upregulation  3.4% mRNA downregulation  0.6% mutation | Negative regulation of cell cycle process; cellular response to DNA damage stimulus |
| DROSHA | Ribonuclease III | **22.7%**  5.1% Amplification  18.8% mRNA upregulation  1.7% mRNA downregulation  0.6% mutation | Not reported |
| PRKDC | DNA-dependent protein kinase catalytic subunit | **22.7%**  7.4% Amplification  13.6% mRNA upregulation  5.7% mutation | Negative regulation of cell aging; regulation of cell cycle G1/S phase transition; negative regulation of cell cycle process; cellular response to DNA damage stimulus; negative regulation of apoptotic process |
| RRM2B | Ribonucleoside-diphosphate reductase subunit M2 B | **18.8%**  7.4% Amplification  14.8% mRNA upregulation | Regulation of signal transduction by p53 class mediator; cellular response to DNA damage stimulus; negative regulation of apoptotic process |
| HIRA | Protein HIRA | **18.1%**  4.5% Amplification  0.6% Homozygous deletion  12.5% mRNA upregulation  3.4% mRNA downregulation  1.1% mutation | Not reported |

C

| **Wild-type TP53 in Oropharynx** | | | |
| --- | --- | --- | --- |
| **Gene** | **Protein** | **Frequency and alteration type** | **Cell function** |
| PCNA | Proliferating cell nuclear antigen | **47.7%**  2.3% Homozygous deletion  45.5% mRNA upregulation | DNA damage response, signal transduction by p53 class mediator; negative regulation of cell cycle process; mismatch repair; rhythmic process; DNA damage response, signal transduction by p53 class mediator resulting in cell cycle arrest; signal transduction involved in mitotic G1 DNA damage checkpoint; negative regulation of cell cycle G1/S phase transition; G1 DNA damage checkpoint; signal transduction involved in DNA damage checkpoint; negative regulation of G1/S transition of mitotic cell cycle; positive regulation of DNA repair; postreplication repair; cellular response to hydrogen peroxide |
| BCL6 | B-cell lymphoma 6 protein | **45.5%**  27.3% Amplification  25% mRNA upregulation  2.3% mutation | B cell differentiation; negative regulation of immunoglobulin production; regulation of chromatin; negative regulation of cellular senescence; negative regulation of apoptotic process; negative regulation of cell cycle process; regulation of B cell apoptotic process; positive regulation of histone deacetylation; negative regulation of cell-matrix adhesion; regulation of regulatory T cell differentiation; regulation of isotype switching; regulation of T-helper cell differentiation |
| FAS | Tumor necrosis factor receptor superfamily member 6 | **36.4%**  6.8% Homozygouse deletion  27.3% mRNA upregulation  2.3% mutation | negative regulation of apoptotic process; necroptotic process; negative regulation of extrinsic apoptotic signaling pathway; activation of cysteine-type endopeptidase activity involved in apoptotic process; fatty acid biosynthetic process; cellular response to starvation |
| GSK3B | Glycogen synthase kinase-3 beta | **34.1%**  6.8% Amplification  29.5% mRNA upregulation  2.3% mRNA downregulation | ER overload response; ER-nucleus signaling pathway; regulation of mitochondrial outer membrane permeabilization involved in apoptotic signaling pathway; positive regulation of protein export from nucleus; negative regulation of apoptotic process; negative regulation of protein acetylation; rhythmic process; extrinsic apoptotic signaling pathway in absence of ligand; peptidyl-threonine phosphorylation; peptidyl-serine phosphorylation; protein autophosphorylation |
| TP63 | Tumor protein 63 | **34.1%**  25% Amplification  13.6% mRNA upregulation  2.3% mRNA downregulation  2.3% mutation | cell aging; aging; negative regulation of cellular senescence; positive regulation of protein insertion into mitochondrial membrane involved in apoptotic signaling pathway; regulation of mitochondrial outer membrane permeabilization involved in apoptotic signaling pathway; negative regulation of intracellular estrogen receptor signaling pathway; intrinsic apoptotic signaling pathway in response to DNA damage by p53 class mediator; intrinsic apoptotic signaling pathway by p53 class mediator; regulation of fibroblast apoptotic process; morphogenesis of embryonic epithelium |
| TSC2 | TSC Complex subunit 2 | **31.8%**  31.8% mRNA upregulation | negative regulation of mitophagy; negative regulation of phosphatidylinositol 3-kinase signaling; protein kinase B signaling; morphogenesis of embryonic epithelium |
| BRCA1 | Breast cancer type 1 susceptbility protein | **31.8%**  29.5% mRNA upregulation  2.3% mutation | positive regulation of histone H3-K9 methylation ; regulation of chromatin organization; negative regulation of intracellular estrogen receptor signaling pathway; DNA damage response, signal transduction by p53 class mediator; negative regulation of apoptotic process; negative regulation of cell cycle process; positive regulation of histone H3-K4 methylation; negative regulation of protein acetylation; signal transduction involved in DNA damage checkpoint; negative regulation of G1/S transition of mitotic cell cycle; regulation of cell cycle G2/M phase transition; G2 DNA damage checkpoint; positive regulation of DNA repair; postreplication repair; negative regulation of reactive oxygen species metabolic process; negative regulation of extrinsic apoptotic signaling pathway; negative regulation of G0 to G1 transition; fatty acid biosynthetic process |
| BCL2 | B-cell lymphoma 2 | **29.5%**  29.5% mRNA upregulation | B cell lineage commitment; B cell differentiation; lymphoid progenitor cell differentiation; cell aging; aging; positive regulation of protein insertion into mitochondrial membrane involved in apoptotic signaling pathway; regulation of mitochondrial outer membrane permeabilization involved in apoptotic signaling pathway; T cell lineage commitment; negative regulation of signal transduction by p53 class mediator; negative regulation of intrinsic apoptotic signaling pathway by p53 class mediator; negative regulation of apoptotic process; negative regulation of cell cycle process; response to UV-B; regulation of myeloid cell apoptotic process; release of cytochrome c from mitochondria; intrinsic apoptotic signaling pathway in response to endoplasmic reticulum stress; negative regulation of cell cycle G1/S phase transition; negative regulation of G1/S transition of mitotic cell cycle; response to gamma radiation; extrinsic apoptotic signaling pathway in absence of ligand; response to iron ion; peptidyl-threonine phosphorylation; response to ischemia; negative regulation of reactive oxygen species metabolic process; negative regulation of extrinsic apoptotic signaling pathway in absence of ligand; negative regulation of extrinsic apoptotic signaling pathway; peptidyl-serine phosphorylation; regulation of viral genome replication; cellular response to starvation |
| MDM2 | E3 ubiquitin protein ligase MDM2 | **29.5%**  29.5% mRNA upregulation | cellular response to actinomycin D; cellular response to UV-C; amyloid fibril formation; positive regulation of protein export from nucleus; DNA damage response, signal transduction by p53 class mediator; negative regulation of DNA damage response, signal transduction by p53 class mediator; negative regulation of signal transduction by p53 class mediator; regulation of DNA damage response, signal transduction by p53 class mediator; negative regulation of intrinsic apoptotic signaling pathway by p53 class mediator; negative regulation of apoptotic process; negative regulation of cell cycle arrest; negative regulation of cell cycle process; DNA damage response, signal transduction by p53 class mediator resulting in cell cycle arrest; signal transduction involved in mitotic G1 DNA damage checkpoint; negative regulation of cell cycle G1/S phase transition; G1 DNA damage checkpoint; signal transduction involved in DNA damage checkpoint; negative regulation of G1/S transition of mitotic cell cycle; response to gamma radiation; response to iron ion; cellular response to hydrogen peroxide; positive regulation of smooth muscle cell proliferation; protein sumoylation |
| LMNB1 | Lamin-B1 | **29.5%**  29.5% mRNA upregulation | Not reported |
| PTEN | Phosphatidylinositol 3,4,5-trisphosphate  3-phosphatase and dual-specificity protein phosphatase PTEN | **27.3%**  15.9% Homozygous deletion  18.2% mRNA downregulation  9.1% mutation | Aging; negative regulation of phosphatidylinositol 3-kinase signaling; negative regulation of apoptotic process; negative regulation of cell cycle process; regulation of B cell apoptotic process; regulation of myeloid cell apoptotic process; circadian behavior; rhythmic process; negative regulation of cell cycle G1/S phase transition; negative regulation of G1/S transition of mitotic cell cycle; regulation of ubiquitin protein ligase activity; negative regulation of cell-matrix adhesion; negative regulation of cyclin-dependent protein serine/threonine kinase activity; protein kinase B signaling; transcription initiation from RNA polymerase II promoter |
| DGCR8 | Microprocessor complex subunit DGCR8 | **27.3%**  25% mRNA upregulation  2.3% mutation | primary miRNA processing; production of miRNAs involved in gene silencing by miRNA; miRNA metabolic process |
| TP73 | Tumor protein P73 | **27.3%**  2.3% Homozygous deletion  25% mRNA upregulation | positive regulation of protein insertion into mitochondrial membrane involved in apoptotic signaling pathway; regulation of mitochondrial outer membrane permeabilization involved in apoptotic signaling pathway; intrinsic apoptotic signaling pathway in response to DNA damage by p53 class mediator; intrinsic apoptotic signaling pathway by p53 class mediator; mismatch repair; regulation of gliogenesis |
| TRIM28 | Transcription intermediary  factor 1-beta | **27.3%**  27.3% mRNA upregulation | regulation of chromatin; positive regulation of DNA repair; positive regulation of protein localization to nucleus; protein sumoylation; regulation of viral genome replication; transcription initiation from RNA polymerase II promoter; protein autophosphorylation; epithelial to mesenchymal transition |
| BCL2L14 | Apoptosis faciliator BCL-2-like protein 14 | **25%**  4.5% Amplification  20.5% mRNA upregulation | Not reported |
| DYRK1A | Dual specificity tyrosin phosphorylation regulated forkhead box A1 | **25%**  22.7% mRNA upregulation  2.3% mutation | negative regulation of DNA damage response, signal transduction by p53 class mediator; negative regulation of signal transduction by p53 class mediator; regulation of DNA damage response, signal transduction by p53 class mediator; rhythmic process; peptidyl-threonine phosphorylation; peptidyl-serine phosphorylation; protein autophosphorylation |
| CCNK | Cyclin K | **25%**  4.5% Homozygous deletion  4.5% mRNA upregulation  20.5% mRNA downregulation | negative regulation of cell cycle arrest; regulation of viral genome replication |
| AGO4 | Protein argonaute 4 | **25%**  15.9% mRNA upregulation  9.1% mutation | production of miRNAs involved in gene silencing by miRNA; pre-miRNA processing; miRNA metabolic process; negative regulation of apoptotic process |
| MSH2 | Muts Homolog II | **22.7%**  22.7% mRNA upregulation | B cell differentiation; aging; regulation of helicase activity; intrinsic apoptotic signaling pathway in response to DNA damage by p53 class mediator; intrinsic apoptotic signaling pathway by p53 class mediator; negative regulation of apoptotic process; response to UV-B; determination of adult lifespan; mismatch repair; somatic recombination of immunoglobulin gene segments; response to X-ray; regulation of isotype switching; postreplication repair |
| PRKAB1 | 5’-AMP-activated protein kinase subunit beta-1 | **22.7%**  22.7% mRNA upregulation | fatty acid biosynthetic process |
| PRKDC | DNA-dependent protein kinase catalytic subunit | **22.7%**  22.7% mRNA upregulation | negative regulation of immunoglobulin production B cell lineage commitment; B cell differentiation; lymphoid progenitor cell differentiation; negative regulation of cellular senescence; T cell lineage commitment; negative regulation of apoptotic process; negative regulation of cell cycle process; rhythmic process; signal transduction involved in mitotic G1 DNA damage checkpoint; negative regulation of cell cycle G1/S phase transition; G1 DNA damage checkpoint; signal transduction involved in DNA damage checkpoint; negative regulation of G1/S transition of mitotic cell cycle; somatic recombination of immunoglobulin gene segments; response to gamma radiation; positive regulation of DNA repair; peptidyl-serine phosphorylation; regulation of fibroblast proliferation |
| CDK1 | Cycline-dependent kinase 1 | **20.5%**  20.5% mRNA upregulation | [cell aging](http://amigo.geneontology.org/amigo/term/GO:0007569); aging; DNA damage response, signal transduction by p53 class mediator; negative regulation of apoptotic process; negative regulation of cell cycle process; rhythmic process; DNA damage response, signal transduction by p53 class mediator resulting in cell cycle arrest; signal transduction involved in mitotic G1 DNA damage checkpoint; negative regulation of cell cycle G1/S phase transition; G1 DNA damage checkpoint; signal transduction involved in DNA damage checkpoint; negative regulation of G1/S transition of mitotic cell cycle; regulation of cell cycle G2/M phase transition; G2 DNA damage checkpoint; peptidyl-threonine phosphorylation; cellular response to hydrogen peroxide; peptidyl-serine phosphorylation; positive regulation of protein localization to nucleus; transcription initiation from RNA polymerase II promoter; regulation of gliogenesis |
| MDM4 | Protein MDM4 | **20.5%**  2.3% Amplification  20.5% mRNA upregulation | DNA damage response, signal transduction by p53 class mediator; negative regulation of apoptotic process; negative regulation of cell cycle arrest; negative regulation of cell cycle process; DNA damage response, signal transduction by p53 class mediator resulting in cell cycle arrest; signal transduction involved in mitotic G1 DNA damage checkpoint; negative regulation of cell cycle G1/S phase transition; G1 DNA damage checkpoint; signal transduction involved in DNA damage checkpoint; negative regulation of G1/S transition of mitotic cell cycle |
| MAPKAPK2 | MAP kinase-activated protein kinase 2 | **20.5%**  2.3% Amplification  18.2% mRNA upregulation  2.3% mRNA downregulation | [peptidyl-serine phosphorylation](http://amigo.geneontology.org/amigo/term/GO:0018105); protein autophosphorylation |
| DROSHA | Drosha  Ribonuclease III | **20.5%**  2.3% Amplification  20.5% mRNA upregulation | primary miRNA processing; production of miRNAs involved in gene silencing by miRNA; [pre-miRNA processing](http://amigo.geneontology.org/amigo/term/GO:0031054), [miRNA metabolic process](http://amigo.geneontology.org/amigo/term/GO:0010586); regulation of regulatory T cell differentiation |
| RRM2B | Ribonucleoside-disphosphate reductase subunit M2 B | **20.5%**  2.3% Amplification  18.2% mRNA upregulation | negative regulation of signal transduction by p53 class mediator; negative regulation of intrinsic ; apoptotic signaling pathway by p53 class mediator; negative regulation of apoptotic process |
| CDKN2A | P16^INK4A^ | **18.2%**  2.3% Homozygouse deletion 13.6% mRNA upregulation  2.3% mutation | amyloid fibril formation; regulation of apoptotic DNA fragmentation; regulation of execution phase of apoptosis; replicative senescence; cell aging; aging; regulation of DNA damage response, signal transduction by p53 class mediator; negative regulation of cell cycle process; regulation of myeloid cell apoptotic process; negative regulation of cell cycle G1/S phase transition; negative regulation of G1/S transition of mitotic cell cycle; regulation of ubiquitin protein ligase activity; negative regulation of cell-matrix adhesion; regulation of cell cycle G2/M phase transition; negative regulation of cyclin-dependent protein serine/threonine kinase activity; positive regulation of protein localization to nucleus; protein sumoylation; activation of cysteine-type endopeptidase activity involved in apoptotic process; chromatin assembly |
| E2F2 | Transcription factor E2F2 | **18.2%**  18.2% mRNA upregulation | intrinsic apoptotic signaling pathway by p53 class mediator; transcription initiation from RNA polymerase II promoter |
| CSNK2A1 | Casein kinase II subunit alfa | **18.2%**  2.3% Homozygous deletion  6.8% mRNA upregulation  9.1% mRNA downregulation  2.3% mutation | negative regulation of apoptotic process; rhythmic process; peptidyl-threonine phosphorylation; peptidyl-serine phosphorylation |
| CX3CL1 | Fractalkine | **18.2%**  2.3% Homozygous deletion  15.9% mRNA upregulation | Aging; negative regulation of apoptotic process; positive regulation of transforming growth factor beta production; negative regulation of cell-matrix adhesion; response to ischemia; negative regulation of extrinsic apoptotic signaling pathway in absence of ligand; negative regulation of extrinsic apoptotic signaling pathway; positive regulation of smooth muscle cell proliferation |
| APAF1 | Apoptotic  protease-activating factor 1 | **18.2%**  18.2% mRNA upregulation | regulation of apoptotic DNA fragmentation; regulation of execution phase of apoptosis; aging; negative regulation of cell cycle process; intrinsic apoptotic signaling pathway in response to endoplasmic reticulum stress; negative regulation of G0 to G1 transition; activation of cysteine-type endopeptidase activity involved in apoptotic process; morphogenesis of embryonic epithelium |
| MYB | Proliferation marker protein Ki-67 | **18.2%**  15.9% mRNA upregulation  2.3% mutation | positive regulation of histone H3-K9 methylation; regulation of chromatin; positive regulation of histone H3-K4 methylation; positive regulation of transforming growth factor beta production; regulation of T-helper cell differentiation; cellular response to hydrogen peroxide; regulation of fibroblast proliferation; positive regulation of smooth muscle cell proliferation; regulation of gliogenesis |
| CABIN1 | Calcineurin-binding protein cabin-1 | **17.5%**  20.5% mRNA upregulation  4.5% mutation | chromatin assembly |

| **Mutated TP53 in Oropharynx**  D | | | |
| --- | --- | --- | --- |
| **Gene** | **Protein** | **Frequency and alteration type** | **Cell function** |
| CDKN2A | P16^INK4A^ | **78.9%**  57.9% Homozygous deletion  21.1% mutation | replicative senescence; cell aging; negative regulation of developmental process; regulation of protein export from nucleus; negative regulation of cell cycle process; negative regulation of cell-matrix adhesion; positive regulation of apoptotic process; negative regulation of B cell activation; regulation of signal transduction by p53 class mediator; apoptotic mitochondrial changes; cell cycle arrest; G1/S transition of mitotic cell cycle; chromatin assembly; positive regulation of protein modification by small protein conjugation or removal; negative regulation of cell growth |
| TP63 | Tumor protein 63 | **52.6%**  42.1% Amplification  21.1% mRNA upregulation | cell aging; negative regulation of cellular senescence; negative regulation of developmental process; morphogenesis of an epithelial fold; positive regulation of protein insertion into mitochondrial membrane involved in apoptotic signaling pathway; positive regulation of mitochondrion organization; positive regulation of apoptotic process; regulation of mitochondrial outer membrane permeabilization involved in apoptotic signaling pathway; regulation of signal transduction by p53 class mediator; regulation of intracellular estrogen receptor signaling pathway; negative regulation of epithelial cell differentiation; stem cell differentiation; positive regulation of epithelial cell proliferation |
| NDRG1 | Protein NDRG1 | **42.1%**  10.5% Amplification  36.8% mRNA upregulation | cellular response to hypoxia; DNA damage response, signal transduction by p53 class mediator |
| BCL6 | B-cell lymphoma 6 protein | **36.38%**  26.3% Amplification  15.8% mRNA upregulation | negative regulation of cellular senescence; negative regulation of developmental process; positive regulation of histone deacetylation; regulation of histone modification; positive regulation of protein deacetylation; negative regulation of cell cycle process; negative regulation of cell-matrix adhesion; positive regulation of apoptotic process; negative regulation of lymphocyte apoptotic process;  regulation of regulatory T cell differentiation; negative regulation of B cell activation; negative regulation of cell growth |
| DROSHA | Drosha ribonuclease III | **36.8%**  15.8% Amplification  26.3% mRNA upregulation  5.3% mutation | regulation of regulatory T cell differentiation |
| PRKAB2 | Protein kinase AMP-activated non-catalytic subunit beta 2 | **26.3%**  10.5% Amplification  15.8% mRNA upregulation | regulation of signal transduction by p53 class mediator; regulation of autophagy; cell cycle arrest; regulation of macroautophagy |
| SKP2 | S-phase kinase associated protein 2 | **26.3%**  15.8% Amplification  15.8% mRNA upregulation | regulation of intracellular estrogen receptor signaling pathway; G1/S transition of mitotic cell cycle; positive regulation of protein modification by small protein conjugation or removal; protein deubiquitination |
| CCNK | Cyclin K | **26.3%**  15.8% mRNA upregulation  10.5% mRNA downregulation | negative regulation of cell cycle arrest; negative regulation of cell cycle process; transcription, DNA-templated; positive regulation of DNA-templated transcription, elongation; ncRNA transcription |
| STEAP3 | STEAP3 metalloreductase | **26.3%**  26.3% mRNA upregulation | iron ion homeostasis |
| HIF1A | Hypoxia inducible factor 1 subunit alpha | **21.1%**  5.3% Amplification  10.5% mRNA upregulation  15.8% mutation | positive regulation of transcription from RNA polymerase II promoter in response to hypoxia; cellular response to hypoxia; regulation of thymocyte apoptotic process; negative regulation of developmental process; morphogenesis of an epithelial fold; positive regulation of glycolytic process; positive regulation of cellular catabolic process; regulation of carbohydrate metabolic process; positive regulation of mitochondrion organization; mRNA transcription; transcription, DNA-templated; negative regulation of lymphocyte apoptotic process; regulation of cellular respiration; positive regulation of pri-miRNA transcription by RNA polymerase II; regulation of autophagy of mitochondrion; regulation of autophagy; iron ion homeostasis; positive regulation of autophagy; stem cell differentiation; protein deubiquitination; regulation of macroautophagy; cellular response to interleukin-1; positive regulation of epithelial cell proliferation |
| PPP2CB | Protein phosphatase 2 catalytic subunit beta | **21.1%**  5.3% Homozygous deletion  10.5% Amplification  5.3% mRNA upregulation  5.3% mRNA downregulation | apoptotic mitochondrial changes; positive regulation of binding |
| YY1 | YY1 transcription factor | **21.1%**  21.1% mRNA upregulation | response to UV-C; response to UV; negative regulation of developmental process; positive regulation of pri-miRNA transcription by RNA polymerase II; negative regulation of cell growth; protein deubiquitination; cellular response to interleukin-1; double-strand break repair; [regulation of cell growth involved in cardiac muscle cell development](http://amigo.geneontology.org/amigo/term/GO:0061050) |
| SERPINE1 | Serpin family E member 1 | **21.1%**  15.8% Amplification  10.5% mRNA upregulation | replicative senescence; cell aging; negative regulation of developmental process; negative regulation of smooth muscle cell migration; regulation of smooth muscle cell migration; negative regulation of cell-matrix adhesion; negative regulation of epithelial cell differentiation; circadian rhythm |
| MYC | MYC proto-oncogene | **21.1 %**  21.1% Ampification | cellular response to hypoxia; response to UV; negative regulation of developmental process; regulation of smooth muscle cell migration; positive regulation of glycolytic process; positive regulation of cellular catabolic process; regulation of carbohydrate metabolic process; positive regulation of apoptotic process; transcription, DNA-templated; beta-catenin-TCF complex assembly; regulation of cellular respiration; liver regeneration; iron ion homeostasis; cell cycle arrest; G1/S transition of mitotic cell cycle; positive regulation of binding; response to ionizing radiation; protein deubiquitination; cellular response to interleukin-1; positive regulation of epithelial cell proliferation |
| PRMT5 | Protein arginine methyltransferase 5 | **21.1%**  21.1 mRNA upregulation | negative regulation of developmental process; regulation of DNA methylation; regulation of DNA methylation; transcription, DNA-templated; liver regeneration; regulation of signal transduction by p53 class mediator; circadian rhythm |
| CDK9 | Cyclin dependent kinase 9 | **21.1%**  10.5% Amplification  21.1% mRNA upregulation | regulation of histone modification; negative regulation of cell cycle arrest; negative regulation of cell cycle process; transcription, DNA-templated; positive regulation of DNA-templated transcription, elongation; ncRNA transcription; positive regulation of binding; positive regulation of protein modification by small protein conjugation or removal |
| GSK3B | GSK-3 beta, GSK3beta isoform, serine/threonine-protein kinase GSK3B | **21.1%**  5.3% Amplification  15.8% mRNA upregulation | ER overload response; negative regulation of developmental process; positive regulation of protein export from nucleus; regulation of protein export from nucleus; positive regulation of cellular catabolic process; regulation of carbohydrate metabolic process; positive regulation of mitochondrion organization; positive regulation of apoptotic process; regulation of mitochondrial outer membrane permeabilization involved in apoptotic signaling pathway; regulation of autophagy; negative regulation of epithelial cell differentiation; circadian rhythm; regulation of glucose metabolic process; positive regulation of autophagy; positive regulation of binding |
| KAT5 | Lysine acetyltransferase 5 | **21.1%**  5.3% Amplification  21.1% mRNA upregulation | DNA damage response, signal transduction by p53 class mediator resulting in transcription of p21 class mediator; DNA damage response, signal transduction by p53 class mediator; positive regulation of cellular catabolic process; beta-catenin-TCF complex assembly; regulation of signal transduction by p53 class mediator; regulation of autophagy; positive regulation of autophagy; response to ionizing radiation; double-strand break repair |
| HIRA | Histone cell cycle regulator | **21.1%**  5.3% Amplification  15.8% mRNA upregulation | transcription, DNA-templated; chromatin assembly |
| XRRC5 | X-ray repair cross complementing 5 | **21.1%**  10.5% mRNA upregulation  10.5% mRNA downregulation  4.2% mutation | Not reported |
| IGFBP3 | Insulin like growth factor binding protein 3 | **21.1%**  21.1% mRNA upregulation | negative regulation of smooth muscle cell migration; regulation of smooth muscle cell migration; regulation of carbohydrate metabolic process; positive regulation of apoptotic process; regulation of glucose metabolic process |
| TTC5 | Tetratricopeptide repeat domain 5 | **21.1%**  21.1% mRNA upregulation | regulation of signal transduction by p53 class mediator |

E

| **Wild-type TP53 in Larynx** | | | |
| --- | --- | --- | --- |
| **Gene** | **Protein** | **Frequency and alteration type** | **Cell function** |
| CDKN2A | P16^INK4A^ | **41.6%**  41.6% Homozygous deletion | regulation of signal transduction by p53 class mediator; DNA conformation change; positive regulation of apoptotic process; |
| CCNK | Cycline K | **33.3%**  8.3% mRNA upregulation  25.0% mRNA downregulation | Not reported |
| BCL6 | B-cell lymphoma 6 protein | **33.3%**  25.0% Amplification  16.7% mRNA upregulation | positive regulation of apoptotic process; |
| SH2D1A | SH2 domain-containing protein 1A | **33.3%**  16.7% mRNA upregulation  16.7% mutation | positive regulation of innate immune response |
| TP63 | Tumor protein 63 | **33.3%**  25.0% Amplification  16.7% mRNA upregulation | regulation of signal transduction by p53 class mediator; positive regulation of apoptotic process |
| CSNK2A1 | Casein kinase II subunit alfa | **33.3%**  8.3% mRNA upregulation  16.7% mRNA downregulation  8.3% mutation | regulation of signal transduction by p53 class mediator; peptidyl-serine phosphorylation; rhythmic process |
| MAPK13 | Mitogen-activated protein kinase 13 | **25%**  16.7% mRNA upregulation  8.3% mutation | stress-activated MAPK cascade; peptidyl-serine phosphorylation; |
| FDXR | NADPH: adrenodoxin oxidoreductase, mitochondrial | **25%**  25.0% mRNA upregulation | Not reported |
| GSK3B | Glycogen synthase kinase-3 beta | **25%**  16.7% Amplification  8.3% mRNA upregulation | peptidyl-serine phosphorylation; rhythmic process; positive regulation of apoptotic process; |
| UBB | Polyubiquitin-B | **25%**  8.3% mRNA upregulation  16.7% mRNA downregulation | stress-activated MAPK cascade; regulation of signal transduction by p53 class mediator; activation of innate immune response; positive regulation of innate immune response; positive regulation of apoptotic process; |
| NEDD8 | NEDD8 | **25%**  8.3% Amplification  16.7% mRNA upregulation | Not reported |
| TRAF6 | TNF receptor-associated  factor 6 | **25%**  8.3% mRNA upregulation  16.7% mRNA downregulation | stress-activated MAPK cascade; activation of innate immune response; positive regulation of innate immune response; positive regulation of apoptotic process; |
| CREBBP | CREB-binding protein | **25%**  8.3% mRNA upregulation  16.7% mRNA downregulation | activation of innate immune response; positive regulation of innate immune response; rhythmic process |
| ASF1A | Histone chaperone ASF1A | **25%**  16.7%% mRNA upregulation  8.3% mRNA downregulation | DNA conformation change |
| MAPKAPK2 | MAP kinase-activated protein kinase 2 | **25%**  25.0% mRNA upregulation | stress-activated MAPK cascade; peptidyl-serine phosphorylation; activation of innate immune response; positive regulation of innate immune response; |
| DYRK1A | Dual specificity tyrosine-phosphorylation-regulated kinase 1A | **25%**  8.3% Amplification  8.3% mRNA upregulation  16.7% mRNA downregulation | regulation of signal transduction by p53 class mediator; peptidyl-serine phosphorylation; rhythmic process |
| XRCC5 | X-ray repair cross-complementing protein 5 | **25%**  8.3% Homozygous deletion  8.3% mRNA upregulation  16.7% mRNA downregulation | activation of innate immune response; positive regulation of innate immune response; DNA conformation change; |
| NDRG1 | Protein NDRG1 | **25%**  25.0% mRNA upregulation | Not reported |

F

| **Mutated TP53 in Larynx** | | | |
| --- | --- | --- | --- |
| **Gene** | **Protein** | **Frequency and alteration type** | **Cell function** |
| CDKN2A | P16^INK4A^ | **56.4%**  33.3% Homozygous deletion  1.3% mRNA upregulation  23.1% mutation | cell aging; regulation of signal transduction by p53 class mediator; regulation of cysteine-type endopeptidase activity involved in apoptotic process; regulation of DNA metabolic process; regulation of hemopoiesis; regulation of growth; regulation of cell cycle process |
| TP63 | Tumor protein 63 | **52.5%**  35.9% Amplification  26.9% mRNA upregulation  1.3% mutation | negative regulation of cellular senescence; positive regulation of mitochondrial outer membrane permeabilization involved in apoptotic signaling pathway; cell aging; regulation of signal transduction by p53 class mediator; regulation of cysteine-type endopeptidase activity involved in apoptotic process; regulation of cell cycle process |
| BCL6 | B-cell lymphoma 6 protein | **47.4%**  32.1% Amplification  25.6% mRNA upregulation  1.3% mutation | negative regulation of cellular senescence; regulation of DNA metabolic process; regulation of hemopoiesis; regulation of growth; regulation of cell cycle process |
| NDRG1 | Protein NDRG1 | **32%**  17.9% Amplification  23.1% mRNA upregulation | cellular response to hypoxia |
| DROSHA | Ribonuclease III | **30.7%**  5.1% Amplification  29.5% mRNA upregulation  1.3% mutation | regulation of hemopoiesis |
| PRKDC | DNA-dependent protein kinase catalytic subunit | **28.2%**  3.8% Amplification  21.8% mRNA upregulation  7.7% mutation | negative regulation of cellular senescence; response to gamma radiation; rhythmic process; regulation of growth; regulation of cell cycle process |
| GSK3B | Glycogen synthase kinase-3 beta | **24.3%**  6.4% Amplification  19.2% mRNA upregulation  2.6% mutation | positive regulation of mitochondrial outer membrane permeabilization involved in apoptotic signaling pathway; rhythmic process; regulation of growth; |
| MYC | MYC proto-oncogene protein | **23%**  23.0% Amplification | response to gamma radiation; cellular response to UV; cellular response to hypoxia; regulation of cysteine-type endopeptidase activity involved in apoptotic process; regulation of DNA metabolic process; regulation of hemopoiesis; |
| COP1 | E3 ubiquitin protein ligase COP1 | **21.7%**  1.3% Amplification  20.5% mRNA upregulation  2.6% mutation | cellular response to UV; cellular response to hypoxia; regulation of cysteine-type endopeptidase activity involved in apoptotic process; |
| PMS2 | Mismatch repair endonuclease PMS2 | **20.5%**  2.6% Amplification  17.9% mRNA upregulation  1.3% mutation | Not reported |
| CDK9 | Cyclin-dependent kinase 9 | **20.5%**  3.8% Amplification  15.4% mRNA upregulation  2.6% mRNA downregulation  2.6% mutation | regulation of DNA metabolic process; regulation of cell cycle process |
| CSNK2A1 | Casein kinase II subunit alfa | **20.5%**  17.9% mRNA upregulation  2.6% mutation | regulation of signal transduction by p53 class mediator; regulation of cysteine-type endopeptidase activity involved in apoptotic process; rhythmic process; regulation of growth; regulation of cell cycle process |
| TNRC6B | Trinucleotide repeat-containing gene 6B protein | **20.5%**  1.3% Amplification  16.7% mRNA upregulation  2.6% mutation | regulation of hemopoiesis; |
| SKP2 | S-phase kinase-associated protein 2 | **19.2%**  3.8% Amplification  17.9% mRNA upregulation | Not reported |
| CREBBP | CREB-binding protein | **19.2%**  1.3% Homozygous deletion  11.5% mRNA upregulation  6.4% mutation | cellular response to UV; cellular response to hypoxia; rhythmic process; regulation of hemopoiesis; |
| WRN | Werner syndrome ATP-dependent elicase | **17.9%**  1.3% Amplification  14.1% mRNA upregulation  1.3% mRNA downregulation  3.8% mutation | response to gamma radiation; cell aging; regulation of signal transduction by p53 class mediator; regulation of DNA metabolic process; regulation of growth; |

G

| **TP53 in HPV negative Oropharynx** | | | |
| --- | --- | --- | --- |
| **Gene** | **Protein** | **Frequency and alteration type** | **Cell function** |
| CDKN2A | P16^INK4A^ | **83.3%**  50.0% Homozygous deletion  33.3% mutation | replicative senescence; positive regulation of cell aging; negative regulation of cell-matrix adhesion; negative regulation of cell cycle process; negative regulation of B cell activation; negative regulation of proteolysis; regulation of DNA damage response, signal transduction by p53 class mediator; regulation of execution phase of apoptosis; cell cycle arrest; positive regulation of protein modification by small protein conjugation or removal; regulation of cysteine-type endopeptidase activity involved in apoptotic process; regulation of cell cycle G1/S phase transition; protein stabilization; negative regulation of cell growth; negative regulation of cellular catabolic process |
| TP63 | Tumor protein 63 | **66.7%**  50% Amplification  33.3.0% mRNA upregulation | stem cell differentiation; negative regulation of cellular senescence; positive regulation of mitochondrial outer membrane permeabilization involved in apoptotic signaling pathway ; intrinsic apoptotic signaling pathway in response to DNA damage by p53 class mediator; regulation of intracellular estrogen receptor signaling pathway; negative regulation of epithelial cell differentiation; regulation of cysteine-type endopeptidase activity involved in apoptotic process; regulation of cell cycle G1/S phase transition |
| PRKAB2 | 5’-AMP-activated protein kinase subunit beta-2 | **50%**  33..3% Amplification  16.7% mRNA upregulation | Lipophagy; carnitine shuttle; fatty acid transmembrane transport; cell cycle arrest; regulation of macroautophagy; regulation of autophagy |
| BCL6 | B-cell lymphoma 6 protein | **50%**  33.3% Amplification  16.7% mRNA upregulation | negative regulation of cell-matrix adhesion; negative regulation of cellular senescence; positive regulation of histone deacetylation; regulation of protein deacetylation; negative regulation of cell cycle process; regulation of regulatory T cell differentiation; negative regulation of B cell activation; negative regulation of DNA replication; regulation of Notch signaling pathway; negative regulation of cell growth; regulation of inflammatory response |
| NDRG1 | Protein NDRG1 | **50%**  16.7% Amplification  33.3% mRNA upregulation | cellular response to hypoxia |
| SERPINE1 | Serpin family E member 1 | **33.3%**  33.3% Amplification  16.7% mRNA upregulation | replicative senescence; negative regulation of cell-matrix adhesion; negative regulation of protein processing; negative regulation of proteolysis; negative regulation of extrinsic apoptotic signaling pathway via death domain receptors; circadian rhythm; regulation of inflammatory response; negative regulation of hydrolase activity |
| XRCC5 | X-ray repair cross-complementing protein 5 | **33.3%**  16.7% mRNA upregulation  16.7% mRNA downregulation | hematopoietic stem cell differentiation; stem cell differentiation; response to salt stress; response to ionizing radiation; cellular response to gamma radiation; regulation of DNA biosynthetic process |
| CREBBP | CREB-binding protein | **33.3%**  33.3% mRNA upregulation | regulation of Notch signaling pathway; circadian rhythm; cellular response to hypoxia; transcription initiation from RNA polymerase II promoter |
| SKP2 | S-phase kinase-associated protein 2 | **33.3%**  33.3% mRNA upregulation | regulation of intracellular estrogen receptor signaling pathway; positive regulation of protein modification by small protein conjugation or removal |
| GSK3B | Glycogen synthase kinase-3 beta | **33.3%**  16.7% Amplification  16.7% mRNA upregulation | ER overload response; ER-nucleus signaling pathway; positive regulation of protein export from nucleus; positive regulation of mitochondrial outer membrane permeabilization involved in apoptotic signaling pathway; negative regulation of epithelial cell differentiation; regulation of circadian rhythm; regulation of proteasomal ubiquitin-dependent protein catabolic process; circadian rhythm; regulation of autophagy; positive regulation of binding; negative regulation of hydrolase activity |
| DROSHA | Ribonuclease III | **33.3%**  33.3% mRNA upregulation | regulation of regulatory T cell differentiation; regulation of inflammatory response |
| BDNF | brain derived neurotrophic factor | **33.3%**33.3% mRNA upregulation | positive regulation of binding; positive regulation of peptidyl-tyrosine phosphorylation |
| PMS2 | Mismatch repair endonuclease PMS2 | **33.3%**  33.3% mRNA upregulation | Not reported |
| HGF | Hepatocyte growth factor | **33.3%**  33.3% Amplification | regulation of neuron projection regeneration; negative regulation of proteolysis; negative regulation of extrinsic apoptotic signaling pathway via death domain receptors; regulation of DNA biosynthetic process; regulation of cysteine-type endopeptidase activity involved in apoptotic process; regulation of autophagy; positive regulation of peptidyl-tyrosine phosphorylation; negative regulation of cellular catabolic process; regulation of inflammatory response; negative regulation of hydrolase activity |
| USP7 | Ubiquitin carboxyl-terminal  hydrolase 7 | **33.3%**  16.7% Amplification  33.3% mRNA upregulation | negative regulation of proteolysis; regulation of circadian rhythm; regulation of proteasomal ubiquitin-dependent protein catabolic process; protein stabilization; negative regulation of cellular catabolic process |
| CDK9 | Cyclin-dependent kinase 9 | **33.3%**  16.7% Amplification  33.3% mRNA upregulation | negative regulation of cell cycle arrest; negative regulation of cell cycle process; positive regulation of DNA-templated transcription, elongation; ncRNA transcription; positive regulation of protein modification by small protein conjugation or removal; positive regulation of binding; transcription initiation from RNA polymerase II promoter |
| CCNK | Cycline K | **33.3%**  33.3% mRNA downregulation | negative regulation of cell cycle arrest; negative regulation of cell cycle process; positive regulation of DNA-templated transcription, elongation; ncRNA transcription |
| PRKAA2 | 5’-AMP-activated protein kinase catalytic subunit alfa-2 | **33.3%**  16.7% Amplification  16.7% mRNA upregulation | Lipophagy; carnitine shuttle; fatty acid transmembrane transport; regulation of protein deacetylation; cellular response to glucose starvation; regulation of circadian rhythm; cell cycle arrest; regulation of macroautophagy; regulation of autophagy |
| MAP4K4 | Mitogen-activated protein kinase kinase kinase  kinase 4 | **33.3%**  33.3% mRNA upregulation | negative regulation of cell-matrix adhesion; regulation of neuron projection regeneration |
| COP1 | E3 ubiquitin protein ligase COP1 | **33.3%**  16.7% Amplification  33.3% mRNA upregulation | cellular response to UV-C; response to ionizing radiation; negative regulation of protein processing; negative regulation of proteolysis; regulation of proteasomal ubiquitin-dependent protein catabolic process; cellular response to hypoxia; regulation of cysteine-type endopeptidase activity involved in apoptotic process; negative regulation of hydrolase activity |

H

| **TP53 in HPV positive Oropharynx** | | | |
| --- | --- | --- | --- |
| **Gene** | **Protein** | **Frequency and alteration type** | **Cell function** |
| PCNA | Proliferating cell nuclear antigen | **50%**  3.7% Homozygous deletion 48.1% mRNA upregulation | [response to UV](http://amigo.geneontology.org/amigo/term/GO:0009411); [regulation of deoxyribonuclease activity](http://amigo.geneontology.org/amigo/term/GO:0032070); [negative regulation of cell cycle process](http://amigo.geneontology.org/amigo/term/GO:0010948); [DNA damage response, signal transduction by p53 class mediator resulting in cell cycle arrest](http://amigo.geneontology.org/amigo/term/GO:0006977); [mismatch repair](http://amigo.geneontology.org/amigo/term/GO:0006298); [regulation of transcription involved in G1/S transition of mitotic cell cycle](http://amigo.geneontology.org/amigo/term/GO:0000083); [liver regeneration](http://amigo.geneontology.org/amigo/term/GO:0097421); [positive regulation of DNA repair](http://amigo.geneontology.org/amigo/term/GO:0045739); [postreplication repair](http://amigo.geneontology.org/amigo/term/GO:0006301); [cellular response to hydrogen peroxide](http://amigo.geneontology.org/amigo/term/GO:0070301); [nucleotide-excision repair](http://amigo.geneontology.org/amigo/term/GO:0006289) |
| FAS | Tumor necrosis factor receptor superfamily member 6 | **37.0%**  7.4% Homozygous deletion 25.9% mRNA upregulation  3.7% mutation | [positive regulation of apoptotic process](http://amigo.geneontology.org/amigo/term/GO:0043065); [negative regulation of apoptotic signaling pathway](http://amigo.geneontology.org/amigo/term/GO:2001234); [necroptotic process](http://amigo.geneontology.org/amigo/term/GO:0070266); [regulation of extrinsic apoptotic signaling pathway via death domain receptors](http://amigo.geneontology.org/amigo/term/GO:1902041); [regulation of extrinsic apoptotic signaling pathway](http://amigo.geneontology.org/amigo/term/GO:2001236); [regulation of cysteine-type endopeptidase activity involved in apoptotic process](http://amigo.geneontology.org/amigo/term/GO:0043281); [fatty acid biosynthetic process](http://amigo.geneontology.org/amigo/term/GO:0006633) |
| BCL6 | B-cell lymphoma 6 protein | **37.0%**  18.5% Amplification  25.9% mRNA upregulation | [negative regulation of B cell apoptotic process](http://amigo.geneontology.org/amigo/term/GO:0002903); [regulation of leukocyte apoptotic process](http://amigo.geneontology.org/amigo/term/GO:2000106); [positive regulation of apoptotic process](http://amigo.geneontology.org/amigo/term/GO:0043065); [negative regulation of cell cycle process](http://amigo.geneontology.org/amigo/term/GO:0010948); [positive regulation of histone deacetylation](http://amigo.geneontology.org/amigo/term/GO:0031065); [negative regulation of cell-matrix adhesion](http://amigo.geneontology.org/amigo/term/GO:0001953); [regulation of cell-matrix adhesion](http://amigo.geneontology.org/amigo/term/GO:0001952); [regulation of regulatory T cell differentiation](http://amigo.geneontology.org/amigo/term/GO:0045589); [regulation of isotype switching](http://amigo.geneontology.org/amigo/term/GO:0045191); [negative regulation of cell growth](http://amigo.geneontology.org/amigo/term/GO:0030308) |
| DGCR8 | Microprocessor complex subunit DGCR8 | **37.0%**  37.0% mRNA upregulation | [primary miRNA processing](http://amigo.geneontology.org/amigo/term/GO:0031053); [production of miRNAs involved in gene silencing by miRNA](http://amigo.geneontology.org/amigo/term/GO:0035196); [miRNA metabolic process](http://amigo.geneontology.org/amigo/term/GO:0010586) |
| BRCA1 | Breast cancer type 1 susceptbility protein | **33.3%**  29.6% mRNA upregulation  3.7% mutation | [positive regulation of histone H3-K9 methylation](http://amigo.geneontology.org/amigo/term/GO:0051574); [negative regulation of intracellular estrogen receptor signaling pathway](http://amigo.geneontology.org/amigo/term/GO:0033147); [regulation of intracellular estrogen receptor signaling pathway](http://amigo.geneontology.org/amigo/term/GO:0033146);  [DNA damage response, signal transduction by p53 class mediator resulting in transcription of p21 class mediator](http://amigo.geneontology.org/amigo/term/GO:0006978); [intrinsic apoptotic signaling pathway in response to DNA damage](http://amigo.geneontology.org/amigo/term/GO:0008630); [negative regulation of cell cycle process](http://amigo.geneontology.org/amigo/term/GO:0010948); [positive regulation of histone H3-K4 methylation](http://amigo.geneontology.org/amigo/term/GO:0051571); [negative regulation of protein acetylation](http://amigo.geneontology.org/amigo/term/GO:1901984); [negative regulation of apoptotic signaling pathway](http://amigo.geneontology.org/amigo/term/GO:2001234); [regulation of protein ubiquitination](http://amigo.geneontology.org/amigo/term/GO:0031396); [regulation of DNA methylation](http://amigo.geneontology.org/amigo/term/GO:0044030); [negative regulation of G2/M transition of mitotic cell cycle](http://amigo.geneontology.org/amigo/term/GO:0010972); [G2 DNA damage checkpoint](http://amigo.geneontology.org/amigo/term/GO:0031572); [positive regulation of DNA repair](http://amigo.geneontology.org/amigo/term/GO:0045739); [postreplication repair](http://amigo.geneontology.org/amigo/term/GO:0006301); [negative regulation of reactive oxygen species metabolic process](http://amigo.geneontology.org/amigo/term/GO:2000378); [regulation of extrinsic apoptotic signaling pathway via death domain receptors](http://amigo.geneontology.org/amigo/term/GO:1902041); [regulation of extrinsic apoptotic signaling pathway](http://amigo.geneontology.org/amigo/term/GO:2001236); [protein autoubiquitination](http://amigo.geneontology.org/amigo/term/GO:0051865); [fatty acid biosynthetic process](http://amigo.geneontology.org/amigo/term/GO:0006633) |
| CCNK | Cycline K | **33.3%**  3.7% Homozygous deletion 11.1% mRNA upregulation  22.2% mRNA downregulation | [negative regulation of cell cycle arrest](http://amigo.geneontology.org/amigo/term/GO:0071157); [negative regulation of cell cycle process](http://amigo.geneontology.org/amigo/term/GO:0010948); [regulation of viral genome replication](http://amigo.geneontology.org/amigo/term/GO:0045069) |
| GSK3B | Glycogen synthase kinase-3 beta | **33.3%**  7.4% Amplification  29.6% mRNA upregulation  3.7% mRNA downregulation | [ER overload response](http://amigo.geneontology.org/amigo/term/GO:0006983); [positive regulation of apoptotic process](http://amigo.geneontology.org/amigo/term/GO:0043065); [positive regulation of protein insertion into mitochondrial membrane involved in apoptotic signaling pathway](http://amigo.geneontology.org/amigo/term/GO:1900740);  [regulation of nucleocytoplasmic transport](http://amigo.geneontology.org/amigo/term/GO:0046822); [peptidyl-threonine phosphorylation](http://amigo.geneontology.org/amigo/term/GO:0018107); [negative regulation of protein acetylation](http://amigo.geneontology.org/amigo/term/GO:1901984); [regulation of cell-matrix adhesion](http://amigo.geneontology.org/amigo/term/GO:0001952); [protein autophosphorylation](http://amigo.geneontology.org/amigo/term/GO:0046777); [protein sumoylation](http://amigo.geneontology.org/amigo/term/GO:0016925) |
| PTEN | Phosphatidylinositol 3,4,5-trisphosphate  3-phosphatase and dual-specificity protein phosphatase PTEN | **29.6%**  14.8% Homozygous deletion  22.2% mRNA downregulation  11.1% mutation | [regulation of leukocyte apoptotic process](http://amigo.geneontology.org/amigo/term/GO:2000106); [negative regulation of phosphatidylinositol 3-kinase signaling](http://amigo.geneontology.org/amigo/term/GO:0014067); [positive regulation of apoptotic process](http://amigo.geneontology.org/amigo/term/GO:0043065); [negative regulation of cell cycle process](http://amigo.geneontology.org/amigo/term/GO:0010948); [negative regulation of cardiac muscle cell proliferation](http://amigo.geneontology.org/amigo/term/GO:0060044); [regulation of cardiac muscle tissue growth](http://amigo.geneontology.org/amigo/term/GO:0055021); [negative regulation of phagocytosis](http://amigo.geneontology.org/amigo/term/GO:0050765); [regulation of ubiquitin protein ligase activity](http://amigo.geneontology.org/amigo/term/GO:1904666); [regulation of protein ubiquitination](http://amigo.geneontology.org/amigo/term/GO:0031396); [negative regulation of cell-matrix adhesion](http://amigo.geneontology.org/amigo/term/GO:0001953); [regulation of cell-matrix adhesion](http://amigo.geneontology.org/amigo/term/GO:0001952); [negative regulation of epithelial to mesenchymal transition](http://amigo.geneontology.org/amigo/term/GO:0010719); [negative regulation of cyclin-dependent protein serine/threonine kinase activity](http://amigo.geneontology.org/amigo/term/GO:0045736); [positive regulation of ubiquitin-protein transferase activity](http://amigo.geneontology.org/amigo/term/GO:0051443); [regulation of extrinsic apoptotic signaling pathway via death domain receptors](http://amigo.geneontology.org/amigo/term/GO:1902041); [regulation of extrinsic apoptotic signaling pathway](http://amigo.geneontology.org/amigo/term/GO:2001236); [protein sumoylation](http://amigo.geneontology.org/amigo/term/GO:0016925) |
| CDK1 | Cycline-dependent kinase 1 | **29.6%**  29.6% mRNA upregulation | [Golgi disassembly](http://amigo.geneontology.org/amigo/term/GO:0090166); [histone phosphorylation](http://amigo.geneontology.org/amigo/term/GO:0016572); [mitotic nuclear envelope disassembly](http://amigo.geneontology.org/amigo/term/GO:0007077); [cell aging](http://amigo.geneontology.org/amigo/term/GO:0007569); [protein localization to kinetochore](http://amigo.geneontology.org/amigo/term/GO:0034501); [protein localization to chromosome, centromeric region](http://amigo.geneontology.org/amigo/term/GO:0071459); [mitotic prophase](http://amigo.geneontology.org/amigo/term/GO:0000088); [negative regulation of cell cycle process](http://amigo.geneontology.org/amigo/term/GO:0010948); [regulation of cardiac muscle tissue growth](http://amigo.geneontology.org/amigo/term/GO:0055021); [DNA damage response, signal transduction by p53 class mediator resulting in cell cycle arrest](http://amigo.geneontology.org/amigo/term/GO:0006977); [peptidyl-threonine phosphorylation](http://amigo.geneontology.org/amigo/term/GO:0018107); [negative regulation of G2/M transition of mitotic cell cycle](http://amigo.geneontology.org/amigo/term/GO:0010972); [G2 DNA damage checkpoint](http://amigo.geneontology.org/amigo/term/GO:0031572); [cellular response to hydrogen peroxide](http://amigo.geneontology.org/amigo/term/GO:0070301) |
| TP63 | Tumor protein 63 | **29.6%**  18.5% Amplification  11.1% mRNA upregulation  3.7% mRNA downregulation | [prostate glandular acinus development](http://amigo.geneontology.org/amigo/term/GO:0060525); [cell aging](http://amigo.geneontology.org/amigo/term/GO:0007569); [epithelial cell differentiation involved in prostate gland development](http://amigo.geneontology.org/amigo/term/GO:0060742); [positive regulation of apoptotic process](http://amigo.geneontology.org/amigo/term/GO:0043065); [positive regulation of protein insertion into mitochondrial membrane involved in apoptotic signaling pathway](http://amigo.geneontology.org/amigo/term/GO:1900740); [negative regulation of intracellular estrogen receptor signaling pathway](http://amigo.geneontology.org/amigo/term/GO:0033147); [regulation of intracellular estrogen receptor signaling pathway](http://amigo.geneontology.org/amigo/term/GO:0033146); [intrinsic apoptotic signaling pathway in response to DNA damage](http://amigo.geneontology.org/amigo/term/GO:0008630); [neuron apoptotic process](http://amigo.geneontology.org/amigo/term/GO:0051402); [prostate gland epithelium morphogenesis](http://amigo.geneontology.org/amigo/term/GO:0060740); [hair follicle morphogenesis](http://amigo.geneontology.org/amigo/term/GO:0031069); [chromatin remodeling](http://amigo.geneontology.org/amigo/term/GO:0006338); [regulation of cysteine-type endopeptidase activity involved in apoptotic process](http://amigo.geneontology.org/amigo/term/GO:0043281) |
| CDKN2A | P16^INK4A^ | **25.9%**  3.7% Homozygous deletion 22.2% mRNA upregulation | [amyloid fibril formation](http://amigo.geneontology.org/amigo/term/GO:1990000); [regulation of leukocyte apoptotic process](http://amigo.geneontology.org/amigo/term/GO:2000106); [replicative senescence](http://amigo.geneontology.org/amigo/term/GO:0090399); [cell aging](http://amigo.geneontology.org/amigo/term/GO:0007569); [positive regulation of apoptotic process](http://amigo.geneontology.org/amigo/term/GO:0043065);  [regulation of nucleocytoplasmic transport](http://amigo.geneontology.org/amigo/term/GO:0046822); [negative regulation of cell cycle process](http://amigo.geneontology.org/amigo/term/GO:0010948); [regulation of ubiquitin protein ligase activity](http://amigo.geneontology.org/amigo/term/GO:1904666); [regulation of protein ubiquitination](http://amigo.geneontology.org/amigo/term/GO:0031396); [negative regulation of cell-matrix adhesion](http://amigo.geneontology.org/amigo/term/GO:0001953); [regulation of cell-matrix adhesion](http://amigo.geneontology.org/amigo/term/GO:0001952); [negative regulation of cyclin-dependent protein serine/threonine kinase activity](http://amigo.geneontology.org/amigo/term/GO:0045736); [cell cycle arrest](http://amigo.geneontology.org/amigo/term/GO:0007050); [chromatin remodeling](http://amigo.geneontology.org/amigo/term/GO:0006338); [negative regulation of cell growth](http://amigo.geneontology.org/amigo/term/GO:0030308); [regulation of cysteine-type endopeptidase activity involved in apoptotic process](http://amigo.geneontology.org/amigo/term/GO:0043281) |
| TRIM28 | Transcription intermediary  factor 1-beta | **25.9%**  25.9% mRNA upregulation | [regulation of nucleocytoplasmic transport](http://amigo.geneontology.org/amigo/term/GO:0046822); [positive regulation of DNA repair](http://amigo.geneontology.org/amigo/term/GO:0045739); [positive regulation of DNA binding](http://amigo.geneontology.org/amigo/term/GO:0043388); [protein autophosphorylation](http://amigo.geneontology.org/amigo/term/GO:0046777); [regulation of viral genome replication](http://amigo.geneontology.org/amigo/term/GO:0045069) |
| DROSHA | Ribonuclease III | **25.9%**  25.9% mRNA upregulation | [primary miRNA processing](http://amigo.geneontology.org/amigo/term/GO:0031053); [production of miRNAs involved in gene silencing by miRNA](http://amigo.geneontology.org/amigo/term/GO:0035196); [pre-miRNA processing](http://amigo.geneontology.org/amigo/term/GO:0031054); [miRNA metabolic process](http://amigo.geneontology.org/amigo/term/GO:0010586); [regulation of regulatory T cell differentiation](http://amigo.geneontology.org/amigo/term/GO:0045589) |
| MDM2 | E3 ubiquitin protein ligase MDM2 | **25.9%**  25.9% mRNA upregulation | cellular response to actinomycin D; [cellular response to UV-C](http://amigo.geneontology.org/amigo/term/GO:0071494); [response to UV](http://amigo.geneontology.org/amigo/term/GO:0009411); [amyloid fibril formation](http://amigo.geneontology.org/amigo/term/GO:1990000);  [regulation of nucleocytoplasmic transport](http://amigo.geneontology.org/amigo/term/GO:0046822);  [negative regulation of DNA damage response, signal transduction by p53 class mediator](http://amigo.geneontology.org/amigo/term/GO:0043518); [negative regulation of cell cycle arrest](http://amigo.geneontology.org/amigo/term/GO:0071157); [negative regulation of cell cycle process](http://amigo.geneontology.org/amigo/term/GO:0010948); [DNA damage response, signal transduction by p53 class mediator resulting in cell cycle arrest](http://amigo.geneontology.org/amigo/term/GO:0006977); [negative regulation of intrinsic apoptotic signaling pathway by p53 class mediator](http://amigo.geneontology.org/amigo/term/GO:1902254); [negative regulation of apoptotic signaling pathway](http://amigo.geneontology.org/amigo/term/GO:2001234); [response to iron ion](http://amigo.geneontology.org/amigo/term/GO:0010039); [cellular response to hydrogen peroxide](http://amigo.geneontology.org/amigo/term/GO:0070301); [protein autoubiquitination](http://amigo.geneontology.org/amigo/term/GO:0051865); [regulation of cysteine-type endopeptidase activity involved in apoptotic process](http://amigo.geneontology.org/amigo/term/GO:0043281) |
| TP73 | Tumor protein P73 | **25.9%**  3.7% Homozygous deletion  22.2% mRNA upregulation | [positive regulation of apoptotic process](http://amigo.geneontology.org/amigo/term/GO:0043065); [positive regulation of protein insertion into mitochondrial membrane involved in apoptotic signaling pathway](http://amigo.geneontology.org/amigo/term/GO:1900740); [intrinsic apoptotic signaling pathway in response to DNA damage](http://amigo.geneontology.org/amigo/term/GO:0008630); [negative regulation of cardiac muscle cell proliferation](http://amigo.geneontology.org/amigo/term/GO:0060044); [regulation of cardiac muscle tissue growth](http://amigo.geneontology.org/amigo/term/GO:0055021); [mismatch repair](http://amigo.geneontology.org/amigo/term/GO:0006298); [positive regulation of oligodendrocyte differentiation](http://amigo.geneontology.org/amigo/term/GO:0048714); [cell cycle arrest](http://amigo.geneontology.org/amigo/term/GO:0007050) |
| LMNB1 | Lamin-B1 | **25.9%**  25.9% mRNA upregulation | Not reported |
| BCL2L14 | Apoptosis faciliator BCL-2-like protein 14 | **25.9%**  25.9% mRNA upregulation | [positive regulation of apoptotic process](http://amigo.geneontology.org/amigo/term/GO:0043065); [regulation of extrinsic apoptotic signaling pathway](http://amigo.geneontology.org/amigo/term/GO:2001236) |
| PRMT5 | Protein arginine N-methyltransferase 5 | **22.2%**  11.1% mRNA upregulation  7.4% mRNA downregulation  3.7% mutation | [histone H4-R3 methylation](http://amigo.geneontology.org/amigo/term/GO:0043985); [peptidyl-arginine omega-N-methylation](http://amigo.geneontology.org/amigo/term/GO:0035247); [positive regulation of oligodendrocyte differentiation](http://amigo.geneontology.org/amigo/term/GO:0048714); [regulation of DNA methylation](http://amigo.geneontology.org/amigo/term/GO:0044030); [liver regeneration](http://amigo.geneontology.org/amigo/term/GO:0097421) |
| MDM4 | Protein MDM4 | **22.2%**  22.2% mRNA upregulation | [negative regulation of cell cycle arrest](http://amigo.geneontology.org/amigo/term/GO:0071157); [negative regulation of cell cycle process](http://amigo.geneontology.org/amigo/term/GO:0010948); [DNA damage response, signal transduction by p53 class mediator resulting in cell cycle arrest](http://amigo.geneontology.org/amigo/term/GO:0006977) |
| BCL2 | Apoptosis regulator BCL-2 | **22.2%**  22.2% mRNA upregulation | [response to UV](http://amigo.geneontology.org/amigo/term/GO:0009411); [cell aging](http://amigo.geneontology.org/amigo/term/GO:0007569); [positive regulation of apoptotic process](http://amigo.geneontology.org/amigo/term/GO:0043065); [positive regulation of protein insertion into mitochondrial membrane involved in apoptotic signaling pathway](http://amigo.geneontology.org/amigo/term/GO:1900740); [intrinsic apoptotic signaling pathway in response to DNA damage](http://amigo.geneontology.org/amigo/term/GO:0008630); [negative regulation of cell cycle process](http://amigo.geneontology.org/amigo/term/GO:0010948); [response to UV-B](http://amigo.geneontology.org/amigo/term/GO:0010224); [neuron apoptotic process](http://amigo.geneontology.org/amigo/term/GO:0051402); [negative regulation of CD4-positive, alpha-beta T cell differentiation](http://amigo.geneontology.org/amigo/term/GO:0043371); [peptidyl-threonine phosphorylation](http://amigo.geneontology.org/amigo/term/GO:0018107); [release of cytochrome c from mitochondria](http://amigo.geneontology.org/amigo/term/GO:0001836); [negative regulation of intrinsic apoptotic signaling pathway by p53 class mediator](http://amigo.geneontology.org/amigo/term/GO:1902254); [negative regulation of apoptotic signaling pathway](http://amigo.geneontology.org/amigo/term/GO:2001234); [hair follicle morphogenesis](http://amigo.geneontology.org/amigo/term/GO:0031069); [regulation of cell-matrix adhesion](http://amigo.geneontology.org/amigo/term/GO:0001952); [response to iron ion](http://amigo.geneontology.org/amigo/term/GO:0010039); [negative regulation of reactive oxygen species metabolic process](http://amigo.geneontology.org/amigo/term/GO:2000378); [regulation of extrinsic apoptotic signaling pathway](http://amigo.geneontology.org/amigo/term/GO:2001236); [negative regulation of cell growth](http://amigo.geneontology.org/amigo/term/GO:0030308); [regulation of viral genome replication](http://amigo.geneontology.org/amigo/term/GO:0045069) |
| AURKB | Aurora kinase B | **22.2%**  22.2% mRNA upregulation | [response to UV](http://amigo.geneontology.org/amigo/term/GO:0009411); [histone-serine phosphorylation](http://amigo.geneontology.org/amigo/term/GO:0035404); [histone phosphorylation](http://amigo.geneontology.org/amigo/term/GO:0016572); [negative regulation of B cell apoptotic process](http://amigo.geneontology.org/amigo/term/GO:0002903); [regulation of leukocyte apoptotic process](http://amigo.geneontology.org/amigo/term/GO:2000106); [protein localization to kinetochore](http://amigo.geneontology.org/amigo/term/GO:0034501); [protein localization to chromosome, centromeric region](http://amigo.geneontology.org/amigo/term/GO:0071459); [negative regulation of cell cycle process](http://amigo.geneontology.org/amigo/term/GO:0010948); [attachment of spindle microtubules to kinetochore](http://amigo.geneontology.org/amigo/term/GO:0008608); [negative regulation of G2/M transition of mitotic cell cycle](http://amigo.geneontology.org/amigo/term/GO:0010972); [regulation of chromosome separation](http://amigo.geneontology.org/amigo/term/GO:1905818); [protein autophosphorylation](http://amigo.geneontology.org/amigo/term/GO:0046777) |
| HMGB1 | High mobility group protein B1 | **22.2%**  22.2% mRNA upregulation | [regulation of deoxyribonuclease activity](http://amigo.geneontology.org/amigo/term/GO:0032070); [positive regulation of apoptotic process](http://amigo.geneontology.org/amigo/term/GO:0043065); [regulation of RNA polymerase II transcriptional preinitiation complex assembly](http://amigo.geneontology.org/amigo/term/GO:0045898); [negative regulation of CD4-positive, alpha-beta T cell differentiation](http://amigo.geneontology.org/amigo/term/GO:0043371); [negative regulation of phagocytosis](http://amigo.geneontology.org/amigo/term/GO:0050765); [positive chemotaxis](http://amigo.geneontology.org/amigo/term/GO:0050918); [positive regulation of DNA repair](http://amigo.geneontology.org/amigo/term/GO:0045739); [positive regulation of DNA binding](http://amigo.geneontology.org/amigo/term/GO:0043388); [chromatin remodeling](http://amigo.geneontology.org/amigo/term/GO:0006338); [lymphocyte activation involved in immune response](http://amigo.geneontology.org/amigo/term/GO:0002285); [regulation of cysteine-type endopeptidase activity involved in apoptotic process](http://amigo.geneontology.org/amigo/term/GO:0043281) |
| DDB2 | DNA  damage-binding protein 2 | **22.2%**  22.2% mRNA upregulation | [response to UV](http://amigo.geneontology.org/amigo/term/GO:0009411); [protein autoubiquitination](http://amigo.geneontology.org/amigo/term/GO:0051865); [nucleotide-excision repair](http://amigo.geneontology.org/amigo/term/GO:0006289) |
| AGO4 | Protein argonaute 4 | **22.2%**  14.8% mRNA upregulation  7.4% mutation | [production of miRNAs involved in gene silencing by miRNA](http://amigo.geneontology.org/amigo/term/GO:0035196); [pre-miRNA processing](http://amigo.geneontology.org/amigo/term/GO:0031054); [miRNA metabolic process](http://amigo.geneontology.org/amigo/term/GO:0010586) |
| YY1 | Transcriptional repressor protein YY1 | **22.2%**  3.7% Homozygous deletion  7.4% mRNA upregulation  11.1% mRNA downregulation | [response to UV](http://amigo.geneontology.org/amigo/term/GO:0009411); [regulation of cardiac muscle tissue growth](http://amigo.geneontology.org/amigo/term/GO:0055021); [negative regulation of cell growth](http://amigo.geneontology.org/amigo/term/GO:0030308) |
| TSC2 | Tuberin | **22.2%**  22.2% mRNA upregulation | [negative regulation of mitophagy](http://amigo.geneontology.org/amigo/term/GO:1901525); [negative regulation of phosphatidylinositol 3-kinase signaling](http://amigo.geneontology.org/amigo/term/GO:0014067); [positive chemotaxis](http://amigo.geneontology.org/amigo/term/GO:0050918) |
| MYB | Proliferation marker protein Ki-67 | **22.2%**  18.5% mRNA upregulation  3.7% mutation | [positive regulation of histone H3-K9 methylation](http://amigo.geneontology.org/amigo/term/GO:0051574); [positive regulation of apoptotic process](http://amigo.geneontology.org/amigo/term/GO:0043065); [positive regulation of histone H3-K4 methylation](http://amigo.geneontology.org/amigo/term/GO:0051571); [positive regulation of transforming growth factor beta production](http://amigo.geneontology.org/amigo/term/GO:0071636); [cellular response to hydrogen peroxide](http://amigo.geneontology.org/amigo/term/GO:0070301); [chromatin remodeling](http://amigo.geneontology.org/amigo/term/GO:0006338) |
| CSNK1G2 | Casein kinase I isoform gamma-2 | **18.5%**  18.5% mRNA upregulation | [peptidyl-threonine phosphorylation](http://amigo.geneontology.org/amigo/term/GO:0018107); [protein autophosphorylation](http://amigo.geneontology.org/amigo/term/GO:0046777) |
| MAPKAPK2 | MAP kinase-activated protein kinase 2 | **18.5%**  14.8% mRNA upregulation  3.7% mRNA downregulation | [protein autophosphorylation](http://amigo.geneontology.org/amigo/term/GO:0046777) |
| PIN1 | Peptidyl-prolyl cis-trans isomerase NIMA-interacting 1 | **18.5%**  18.5% mRNA upregulation | [positive regulation of apoptotic process](http://amigo.geneontology.org/amigo/term/GO:0043065); [regulation of cell growth involved in cardiac muscle cell development](http://amigo.geneontology.org/amigo/term/GO:0061050); [regulation of cardiac muscle tissue growth](http://amigo.geneontology.org/amigo/term/GO:0055021); [regulation of protein ubiquitination](http://amigo.geneontology.org/amigo/term/GO:0031396); [positive regulation of ubiquitin-protein transferase activity](http://amigo.geneontology.org/amigo/term/GO:0051443) |
| CX3CL1 | Fractalkine | **18.5%**  3.7% Homozygous deletion  14.8% mRNA upregulation | [synapse pruning](http://amigo.geneontology.org/amigo/term/GO:0098883); [positive regulation of transforming growth factor beta production](http://amigo.geneontology.org/amigo/term/GO:0071636); [negative regulation of apoptotic signaling pathway](http://amigo.geneontology.org/amigo/term/GO:2001234); [regulation of cell-matrix adhesion](http://amigo.geneontology.org/amigo/term/GO:0001952); [positive chemotaxis](http://amigo.geneontology.org/amigo/term/GO:0050918); [regulation of extrinsic apoptotic signaling pathway](http://amigo.geneontology.org/amigo/term/GO:2001236) |
| PRMT1 | Protein arginine N-metyltranspherase 1 | **18.5%**  18.5% mRNA upregulation | [histone H4-R3 methylation](http://amigo.geneontology.org/amigo/term/GO:0043985); [peptidyl-arginine omega-N-methylation](http://amigo.geneontology.org/amigo/term/GO:0035247); [negative regulation of cell cycle process](http://amigo.geneontology.org/amigo/term/GO:0010948); [DNA damage response, signal transduction by p53 class mediator resulting in cell cycle arrest](http://amigo.geneontology.org/amigo/term/GO:0006977) |
| CABIN1 | Calcineurin-binding protein cabin-1 | **18.5%**  18.5% mRNA upregulation | Not reported |
| RB1 | Retinoblastoma-associated protein | **18.5%**  3.7% Homozygous deletion  7.4% mRNA downregulation  14.8% mutation | [protein localization to chromosome, centromeric region](http://amigo.geneontology.org/amigo/term/GO:0071459); [mitotic prophase](http://amigo.geneontology.org/amigo/term/GO:0000088); [negative regulation of cell cycle process](http://amigo.geneontology.org/amigo/term/GO:0010948): [neuron apoptotic process](http://amigo.geneontology.org/amigo/term/GO:0051402); [attachment of spindle microtubules to kinetochore](http://amigo.geneontology.org/amigo/term/GO:0008608); [negative regulation of apoptotic signaling pathway](http://amigo.geneontology.org/amigo/term/GO:2001234); [regulation of transcription involved in G1/S transition of mitotic cell cycle](http://amigo.geneontology.org/amigo/term/GO:0000083); [positive regulation of DNA binding](http://amigo.geneontology.org/amigo/term/GO:0043388); [regulation of chromosome separation](http://amigo.geneontology.org/amigo/term/GO:1905818); [cell cycle arrest](http://amigo.geneontology.org/amigo/term/GO:0007050); [chromatin remodeling](http://amigo.geneontology.org/amigo/term/GO:0006338) |
| DYRK1A | Dual specificity tyrosine-phosphorylation-regulated kinase 1A | **18.5%**  18.5% mRNA upregulation | [negative regulation of DNA damage response, signal transduction by p53 class mediator](http://amigo.geneontology.org/amigo/term/GO:0043518); [peptidyl-threonine phosphorylation](http://amigo.geneontology.org/amigo/term/GO:0018107); [protein autophosphorylation](http://amigo.geneontology.org/amigo/term/GO:0046777) |
| CDK5 | Cyclin-dependent-like kinase 5 | **18.5%**  7.4% Homozygous deletion  11.1% mRNA upregulation | [synapse pruning](http://amigo.geneontology.org/amigo/term/GO:0098883); [histone phosphorylation](http://amigo.geneontology.org/amigo/term/GO:0016572); [positive regulation of apoptotic process](http://amigo.geneontology.org/amigo/term/GO:0043065);  [regulation of nucleocytoplasmic transport](http://amigo.geneontology.org/amigo/term/GO:0046822); [neuron apoptotic process](http://amigo.geneontology.org/amigo/term/GO:0051402); [peptidyl-threonine phosphorylation](http://amigo.geneontology.org/amigo/term/GO:0018107); [regulation of protein ubiquitination](http://amigo.geneontology.org/amigo/term/GO:0031396); [protein autophosphorylation](http://amigo.geneontology.org/amigo/term/GO:0046777); [protein sumoylation](http://amigo.geneontology.org/amigo/term/GO:0016925); [negative regulation of cell growth](http://amigo.geneontology.org/amigo/term/GO:0030308) |
| CSNK2A1 | Casein kinase II subunit alfa | **18.5%**  3.7% Homozygous deletion  14.8% mRNA downregulation  3.7% mutation | [peptidyl-threonine phosphorylation](http://amigo.geneontology.org/amigo/term/GO:0018107); [negative regulation of apoptotic signaling pathway](http://amigo.geneontology.org/amigo/term/GO:2001234); [regulation of chromosome separation](http://amigo.geneontology.org/amigo/term/GO:1905818); [regulation of cysteine-type endopeptidase activity involved in apoptotic process](http://amigo.geneontology.org/amigo/term/GO:0043281) |
| PRKAB1 | 5’-AMP-activated protein kinase subunit beta-1 | **18.5%**  18.5% mRNA upregulation | [cell cycle arrest](http://amigo.geneontology.org/amigo/term/GO:0007050); [fatty acid biosynthetic process](http://amigo.geneontology.org/amigo/term/GO:0006633) |
| VRK1 | Serine/threonine-protain kinase VRK1 | **18.5%**  3.7% Homozygous deletion  14.8% mRNA upregulation | [Golgi disassembly](http://amigo.geneontology.org/amigo/term/GO:0090166); [histone-serine phosphorylation](http://amigo.geneontology.org/amigo/term/GO:0035404); [histone phosphorylation](http://amigo.geneontology.org/amigo/term/GO:0016572); [mitotic nuclear envelope disassembly](http://amigo.geneontology.org/amigo/term/GO:0007077); [peptidyl-threonine phosphorylation](http://amigo.geneontology.org/amigo/term/GO:0018107); [protein autophosphorylation](http://amigo.geneontology.org/amigo/term/GO:0046777) |
| MSH2 | DNA missmatch repair protein MSH2 | **18.5%**  18.5% mRNA upregulation | [response to UV](http://amigo.geneontology.org/amigo/term/GO:0009411); [regulation of helicase activity](http://amigo.geneontology.org/amigo/term/GO:0051095); [intrinsic apoptotic signaling pathway in response to DNA damage](http://amigo.geneontology.org/amigo/term/GO:0008630); [response to UV-B](http://amigo.geneontology.org/amigo/term/GO:0010224); [mismatch repair](http://amigo.geneontology.org/amigo/term/GO:0006298); [regulation of isotype switching](http://amigo.geneontology.org/amigo/term/GO:0045191); [postreplication repair](http://amigo.geneontology.org/amigo/term/GO:0006301); [cell cycle arrest](http://amigo.geneontology.org/amigo/term/GO:0007050); [lymphocyte activation involved in immune response](http://amigo.geneontology.org/amigo/term/GO:0002285) |
| CCNG1 | Cyclin-G1 | **18.5%**  18.5% mRNA upregulation | Not reported |
| FOXA1 | Forkhead box A1 | **18.5%**  3.7% Homozygous deletion  14.8% mRNA upregulation | [prostate glandular acinus development](http://amigo.geneontology.org/amigo/term/GO:0060525); [epithelial cell differentiation involved in prostate gland development](http://amigo.geneontology.org/amigo/term/GO:0060742); [positive regulation of apoptotic process](http://amigo.geneontology.org/amigo/term/GO:0043065); [regulation of intracellular estrogen receptor signaling pathway](http://amigo.geneontology.org/amigo/term/GO:0033146); [prostate gland epithelium morphogenesis](http://amigo.geneontology.org/amigo/term/GO:0060740); [negative regulation of epithelial to mesenchymal transition](http://amigo.geneontology.org/amigo/term/GO:0010719); [chromatin remodeling](http://amigo.geneontology.org/amigo/term/GO:0006338) |

| **Wild-type TP53 in HP**  I | | | |
| --- | --- | --- | --- |
| **Gene** | **Protein** | **Frequency and alteration type** | **Cell function** |
| CDKN2A | P16^INK4A^ | **75.0%**  50.0% Homozygous deletion  25.0% mRNA upregulation | apoptotic mitochondrial changes; regulation of apoptotic DNA fragmentation; replicative senescence; regulation of DNA damage response, signal transduction by p53 class mediator; regulation of signal transduction by p53 class mediator; regulation of cell cycle G1/S phase transition; negative regulation of mitotic cell cycle; regulation of protein export from nucleus; regulation of mitochondrial membrane potential; activation of cysteine-type endopeptidase activity involved in apoptotic process; negative regulation of cell-substrate adhesion; organelle disassembly; regulation of cell-matrix adhesion; G1/S transition of mitotic cell cycle; Ras protein signal transduction; negative regulation of proteolysis |
| PLK3 | Polo like kinase 3 | **75.0%**  50.0% mRNA upregulation  25.0% mutation | positive regulation of chaperone-mediated autophagy; negative regulation of apoptotic process; regulation of signal transduction by p53 class mediator; DNA damage response, signal transduction by p53 class mediator resulting in cell cycle arrest; regulation of cell cycle G1/S phase transition; G1 DNA damage checkpoint; negative regulation of mitotic cell cycle; organelle disassembly; regulation of cytokinesis; G1/S transition of mitotic cell cycle |
| BCL2L1 | BCL2 like 1 | **75.0%**  50.0% Amplification  75.0% mRNA upregulation | apoptotic mitochondrial changes; extrinsic apoptotic signaling pathway; release of cytochrome c from mitochondria; mitochondrion morphogenesis; neuron apoptotic process; cellular response to gamma radiation; response to gamma radiation; extrinsic apoptotic signaling pathway in absence of ligand; negative regulation of extrinsic apoptotic signaling pathway in absence of ligand; negative regulation of apoptotic process; negative regulation of mitotic cell cycle; positive regulation of intrinsic apoptotic signaling pathway; intrinsic apoptotic signaling pathway in response to DNA damage; regulation of mitochondrial membrane potential; ovarian follicle development; negative regulation of autophagy; regulation of response to endoplasmic reticulum stress; regulation of cytokinesis; negative regulation of neuron apoptotic process; male gonad development; cellular response to organonitrogen compound |
| CX3CL1 | Fractalkine | **50.0%**  50.0% mRNA upregulation | positive regulation of calcium-independent cell-cell adhesion; regulation of neuroblast proliferation; regulation of stem cell proliferation; negative regulation of extrinsic apoptotic signaling pathway in absence of ligand; negative regulation of apoptotic process; positive regulation of release of sequestered calcium ion into cytosol; response to ischemia; negative regulation of cell-substrate adhesion; negative regulation of neuron apoptotic process; regulation of cell-matrix adhesion; negative regulation of neurogenesis |
| BAX | BCL2 associated X | **50.0%**  50.0% mRNA upregulation | release of matrix enzymes from mitochondria; apoptotic mitochondrial changes; B cell receptor apoptotic signaling pathway; extrinsic apoptotic signaling pathway; regulation of apoptotic DNA fragmentation; release of cytochrome c from mitochondria; positive regulation of execution phase of apoptosis; mitochondrion morphogenesis; neuron apoptotic process; regulation of mitochondrial membrane permeability involved in apoptotic process; response to gamma radiation; positive regulation of release of cytochrome c from mitochondria; extrinsic apoptotic signaling pathway in absence of ligand; intrinsic apoptotic signaling pathway in response to endoplasmic reticulum stress; negative regulation of apoptotic process; positive regulation of mitochondrial outer membrane permeabilization involved in apoptotic signaling pathway; DNA damage response, signal transduction by p53 class mediator resulting in cell cycle arrest; regulation of cell cycle G1/S phase transition; G1 DNA damage checkpoint; negative regulation of mitotic cell cycle; positive regulation of intrinsic apoptotic signaling pathway; positive regulation of release of sequestered calcium ion into cytosol; intrinsic apoptotic signaling pathway in response to DNA damage; regulation of mitochondrial membrane potential; ovarian follicle development; activation of cysteine-type endopeptidase activity involved in apoptotic process; regulation of response to endoplasmic reticulum stress; negative regulation of neuron apoptotic process; male gonad development |
| NGFR | Nerve growth factor receptor | **50.0%**  50.0% mRNA upregulation | neuron apoptotic process; positive regulation of pri-miRNA transcription by RNA polymerase II; negative regulation of apoptotic process; activation of cysteine-type endopeptidase activity involved in apoptotic process; Ras protein signal transduction; negative regulation of neurogenesis; negative regulation of proteolysis; cellular response to organonitrogen compound |

J

| **Mutated TP53 in HP** | | | |
| --- | --- | --- | --- |
| **Gene** | **Protein** | **Frequency and alteration type** | **Cell function** |
| CDKN2A | P16^INK4A^ | **80.0%**  60.0% Homozygous deletion  20.0% mutation | Autophagy; replicative senescence; regulation of DNA damage response, signal transduction by p53 class mediator; regulation of signal transduction by p53 class mediator; negative regulation of protein phosphorylation; protein destabilization; apoptotic mitochondrial changes; activation of cysteine-type endopeptidase activity involved in apoptotic process; chromatin assembly; positive regulation of response to DNA damage stimulus; negative regulation of G1/S transition of mitotic cell cycle; regulation of nucleocytoplasmic transport; cell cycle arrest; negative regulation of catabolic process |
| DDIT4 | DNA damage inducible transcript 4 | **60.0%**  60.0% mRNA upregulation | negative regulation of ATP metabolic process; regulation of ATP metabolic process; negative regulation of peptidyl-serine phosphorylation; negative regulation of protein phosphorylation; intrinsic apoptotic signaling pathway in response to DNA damage by p53 class mediator; regulation of glycolytic process; negative regulation of small molecule metabolic process; negative regulation of catabolic process |
| PMS2 | Mismatch repair endonuclease PMS2 | **60.0%**  20.0% Amplification  40.0% mRNA upregulation  20.0% mutation | DNA repair |
| PRKAB2 | Protein kinase AMP-activated non-catalytic subunit beta 2 | **60.0%**  20.0% Homozygous deletion  20.0% Amplification  20.0% mRNA upregulation | Lipophagy; autophagy; regulation of signal transduction by p53 class mediator; regulation of macroautophagy; cell cycle arrest; fatty acid biosynthetic process |
| SNAI2 | Snail family transcriptional repressor 2 | **60.0%**  40.0% Amplification  40.0% mRNA upregulation | regulation of branching involved in salivary gland morphogenesis; stem cell differentiation; negative regulation of stem cell proliferation; regulation of DNA damage response, signal transduction by p53 class mediator; regulation of signal transduction by p53 class mediator; positive regulation of histone acetylation; positive regulation of chromosome organization; regulation of apoptotic signaling pathway; negative regulation of signal transduction by p53 class mediator; epithelial to mesenchymal transition; negative regulation of extrinsic apoptotic signaling pathway; negative regulation of intrinsic apoptotic signaling pathway; negative regulation of small molecule metabolic process |
| HGF | Hepatocyte growth factor | **40.0%**  40.0% mRNA upregulation | regulation of branching involved in salivary gland morphogenesis; negative regulation of peptidyl-serine phosphorylation; negative regulation of protein phosphorylation; regulation of apoptotic signaling pathway; epithelial to mesenchymal transition; negative regulation of extrinsic apoptotic signaling pathway; regulation of DNA biosynthetic process; negative regulation of catabolic process |
| HIF1A | Hypoxia inducible factor 1 subunit alpha | **40.0%**  20.0% Amplification  20.0% mRNA upregulation | positive regulation of transcription from RNA polymerase II promoter in response to hypoxia; stem cell differentiation; positive regulation of autophagy of mitochondrion; regulation of ATP metabolic process; mRNA transcription; regulation of apoptotic signaling pathway; positive regulation of pri-miRNA transcription by RNA polymerase II; regulation of pri-miRNA transcription by RNA polymerase II; epithelial to mesenchymal transition; regulation of glycolytic process; negative regulation of intrinsic apoptotic signaling pathway; regulation of macroautophagy; protein deubiquitination |
| HIRA | Histone cell cycle regulator | **40.0%**  40.0% mRNA downregulation | regulation of chromatin silencing; chromatin assembly |
| HIST1H1D | Histone H1.3 | **40.0%**  40.0% mRNA upregulation | regulation of chromatin silencing; negative regulation of DNA metabolic process; chromatin assembly |
| HTT | Huntingtin | **40.0%**  40.0% mRNA downregulation | positive regulation of autophagy of mitochondrion; negative regulation of neural precursor cell proliferation; regulation of apoptotic signaling pathway; vasoconstriction; protein destabilization; negative regulation of organ growth; negative regulation of extrinsic apoptotic signaling pathway; regulation of macroautophagy; rhythmic process |
| CSNK2A1 | Casein kinase II subunit alfa | **40.0%**  20.0% mRNA upregulation  20.0% mRNA downregulation | Autophagy; regulation of signal transduction by p53 class mediator; regulation of apoptotic signaling pathway; negative regulation of catabolic process; rhythmic process |
| PPP2CB | Protein phosphatase 2 catalytic subunit beta | **40.0%**  20.0% Homozygous deletion  20.0% mRNA downregulation | peptidyl-threonine dephosphorylation; apoptotic mitochondrial changes |
| DUSP5 | Dual specificity phosphatase 5 | **40.0%**  40.0% mRNA upregulation | peptidyl-threonine dephosphorylation; negative regulation of protein phosphorylation |
| PRKAG1 | Protein kinase AMP-activated non-catalytic subunit gamma 1 | **40.0%**  20.0% Homozygous deletion  20.0% mRNA upregulation  20.0% mRNA downregulation | Lipophagy; autophagy; regulation of signal transduction by p53 class mediator; regulation of ATP metabolic process; regulation of glycolytic process; regulation of macroautophagy; cell cycle arrest; fatty acid biosynthetic process |
| PRKDC | Protein kinase, DNA-activated, catalytic subunit | **40.0%**  20.0% Amplification  20.0% mRNA upregulation | B cell lineage commitment; T cell lineage commitment; negative regulation of protein phosphorylation; protein destabilization; positive regulation of response to DNA damage stimulus; negative regulation of G1/S transition of mitotic cell cycle; regulation of type I interferon production; double-strand break repair; DNA repair; rhythmic process |
| RRM2B | Ribonucleotide reductase regulatory TP53 inducible subunit M2B | **40.0%**  20.0% Amplification  40.0% mRNA upregulation | regulation of signal transduction by p53 class mediator; regulation of intrinsic apoptotic signaling pathway by p53 class mediator; regulation of apoptotic signaling pathway; negative regulation of signal transduction by p53 class mediator; negative regulation of intrinsic apoptotic signaling pathway; DNA repair |
| EDN2 | Endothelin 2 | **40.0%**  40.0% mRNA upregulation | Vasoconstriction; fatty acid biosynthetic process |
| TADA2B | Transcriptional adaptor 2B | **40.0%**  40.0% mRNA downregulation | positive regulation of histone acetylation; positive regulation of chromosome organization; protein deubiquitination |
| TNFRSF10B | TNF receptor superfamily member 10b | **40.0%**  20.0% Homozygous deletion  20.0% mRNA upregulation | regulation of apoptotic signaling pathway; intrinsic apoptotic signaling pathway in response to endoplasmic reticulum stress; negative regulation of extrinsic apoptotic signaling pathway; activation of cysteine-type endopeptidase activity involved in apoptotic process |
| TP53RK | TP53 regulating kinase | **40.0%**  40.0% mRNA upregulation | regulation of signal transduction by p53 class mediator |
| TRIM28 | Tripartite motif containing 28 | **40.0%**  40.0% mRNA upregulation | regulation of chromatin silencing; positive regulation of chromosome organization; negative regulation of DNA metabolic process; positive regulation of response to DNA damage stimulus; regulation of nucleocytoplasmic transport; DNA repair |
| XRCC5 | X-ray repair cross complementing 5 | **40.0%**  20.0% mRNA downregulation  20.0% mutation | hematopoietic stem cell differentiation; stem cell differentiation; response to salt stress; response to X-ray; cellular response to gamma radiation; positive regulation of chromosome organization; negative regulation of DNA metabolic process; regulation of DNA biosynthetic process; regulation of type I interferon production; double-strand break repair; DNA repair |
| YY1 | YY1 transcription factor | **40.0%**  20.0% mRNA upregulation  20.0% mRNA downregulation | response to UV-C; regulation of pri-miRNA transcription by RNA polymerase II; negative regulation of organ growth; regulation of type I interferon production; double-strand break repair; DNA repair; protein deubiquitination |
| CASP1 | Caspase 1 | **40.0%**  40.0% Amplification  20.0% mRNA upregulation | activation of cysteine-type endopeptidase activity involved in apoptotic process |

Supplemental material Table 4: Bonferroni post-hoc adjusted p-values for Chi-square test comparisons between smoking history and secondary structure.

Supplemental material Table 5:

1. Bonferroni post-hoc for multiple comparisons for the multivariate survival analysis in Figure 5A;
2. Bonferroni post-hoc for multiple comparisons for the multivariate survival analysis in Figure 5B;

| **Table 5A** | **Contrast** | **Std. Err.** | **Bonferroni** | | **Bonferroni** | |
| --- | --- | --- | --- | --- | --- | --- |
|  |  |  |  |  |  |  |
|  |  |  | **z** | **P> I z I** | **[95% Conf. Interval]** | |
|  |  |  |  |  |  |  |
| **Stage** |  | | | | | |
|  |  |  |  |  |  |  |
| Stage 2 vs Stage 1 | 0.1646011 | 0.4184351 | 0.39 | 1 | -0.9393384 | 1.268541 |
| Stage 3 vs Stage 1 | 0.2148694 | 0.4164668 | 0.52 | 1 | -0.8838773 | 1.313616 |
| Stage 4 vs Stage 1 | 0.2496859 | 0.3951198 | 0.63 | 1 | -0.7927417 | 1.292114 |
| Stage 3 vs Stage 2 | 0.0502683 | 0.2460963 | 0.20 | 1 | -0.5989971 | 0.6995337 |
| Stage 4 vs Stage 2 | 0.0850848 | 0.2080701 | 0.41 | 1 | -0.4638576 | 0.6340273 |
| Stage 4 vs Stage 3 | 0.0348166 | 0.2006723 | 0.17 | 1 | -0.4946085 | 0.5642417 |
| **Grading** |  | | | | | |
|  |  |  |  |  |  |  |
|  |  |  |  |  |  |  |
| G2 vs G1 | 0.6052222 | 0.2713379 | 2.23 | 0.077 | -0.0443553 | 1.2548 |
| G3 vs G1 | 0.5337328 | 0.2893503 | 1.84 | 0.195 | -0.1589659 | 1.226432 |
| G3 vs G2 | -0.714893 | 0.1868386 | -0.38 | 1 | -0.5187771 | 0.3757984 |
| **Poeta's algorithm** |  | | | | | |
|  |  |  |  |  |  |  |
|  |  |  |  |  |  |  |
| Non-disruptive vs WT | 0.3554197 | 0.2223825 | 1.60 | 0.330 | -0.1769594 | 0.8877988 |
| Disruptive vs WT | 0.4640012 | 0.2024946 | 2.29 | 0.066 | -0.0207667 | 0.9487692 |
| Disruptive vs non-disruptive | 0.1085815 | 0.1858309 | 0.58 | 1 | -0.3362939 | 0.553457 |
| **Gender** |  | | | | | |
|  |  |  |  |  |  |  |
|  |  |  |  |  |  |  |
| Female vs Male | 0.2220514 | 0.1745023 | 1.27 | 0.203 | -0.1199668 | 0.5640695 |

| **Table 5B** | **Contrast** | **Std. Err.** | **Bonferroni** | | **Bonferroni** | |
| --- | --- | --- | --- | --- | --- | --- |
|  |  |  |  |  |  |  |
|  |  |  | **z** | **P> I z I** | **[95% Conf. Interval]** | |
|  |  |  |  |  |  |  |
| **Stage** |  | | | | | |
|  |  |  |  |  |  |  |
| Stage 2 vs Stage 1 | 0.2174642 | 0.41876 | 0.52 | 1 | -0.8873324 | 1.322261 |
| Stage 3 vs Stage 1 | 0.3181026 | 0.4175302 | 0.76 | 1 | -0.7834494 | 1.419655 |
| Stage 4 vs Stage 1 | 0.2761439 | 0.3952636 | 0.70 | 1 | -0.7666633 | 1.318951 |
| Stage 3 vs Stage 2 | 0.1006384 | 0.2448559 | 0.41 | 1 | -0.5453544 | 0.7466313 |
| Stage 4 vs Stage 2 | 0.0586797 | 0.2079253 | 0.28 | 1 | -0.4898807 | 0.6072401 |
| Stage 4 vs Stage 3 | -0.0419587 | 0.2007994 | -0.21 | 1 | -0.5717192 | 0.4878018 |
| **Grading** |  | | | | | |
|  |  |  |  |  |  |  |
|  |  |  |  |  |  |  |
| G2 vs G1 | 0.58679 | 0.2702638 | 2.17 | 0.090 | -0.0602161 | 1.233796 |
| G3 vs G1 | 0.5436906 | 0.2892218 | 1.88 | 0.180 | -0.1487007 | 1.236082 |
| G3 vs G2 | -0.0430994 | 0.1871593 | -0.23 | 1 | -0.4911551 | 0.4049563 |
| **Our algorithm** |  | | | | | |
|  |  |  |  |  |  |  |
|  |  |  |  |  |  |  |
| Low-risk vs WT | -0.0627979 | 0.2718416 | -0.23 | 1 | -0.7135812 | 0.5879854 |
| High-risk vs WT | 0.5602486 | 0.194406 | 2.88 | 0.012 | 0.0948447 | 1.025653 |
| High-risk vs Low-risk | 0.6230465 | 0.2358845 | 2.64 | 0.025 | 0.0583437 | 1.187749 |
| **Gender** |  | | | | | |
|  |  |  |  |  |  |  |
|  |  |  |  |  |  |  |
| Female vs Male | 0.1935592 | 0.1746491 | 1.11 | 0.268 | -0.1487467 | 0.5358651 |

Supplemental material Figure 1: Multivariate Overall survival in larynx for TP53 mutations in DBD versus N-term.

Supplemental material Figure 2: Multivariate Overall survival in HNSCC for TP53 mutations in unknown secondary structure versus wild-type.

Supplemental material Figure 3: Univariate analysis for overall survival according to R213 MUT.

Supplemental material Figure 4: Two-way ANOVA showing the differential mRNA expression according to the mutational TP53 status and anatomical subsite.

Supplemental material Figure 5: Spearman’s rank correlation results from linear variables.

Supplemental material Figure 6: Gene network analysis for WT OSCC from cBioPortal tool. Network legend: Blue line: Controls state change of; Green line: Controls expression of; Brown line: In complex with.

Supplemental material Figure 7: Gene network analysis for MUT OSCC from cBioPortal tool. Network legend: Blue line: Controls state change of; Green line: Controls expression of; Brown line: In complex with.

Supplemental material Figure 8: Gene network analysis for WT OP from cBioPortal tool. Network legend: Blue line: Controls state change of; Green line: Controls expression of; Brown line: In complex with.

Supplemental material Figure 9: Gene network analysis for MUT OP from cBioPortal tool. Network legend: Blue line: Controls state change of; Green line: Controls expression of; Brown line: In complex with.

Supplemental material Figure 10: Gene network analysis for WT L from cBioPortal tool. Network legend: Blue line: Controls state change of; Green line: Controls expression of; Brown line: In complex with.

Supplemental material Figure 11: Gene network analysis for MUT L from cBioPortal tool. Network legend: Blue line: Controls state change of; Green line: Controls expression of; Brown line: In complex with.

Supplemental material Figure 12: Gene network analysis for HPV negative OP from cBioPortal tool. Network legend: Blue line: Controls state change of; Green line: Controls expression of; Brown line: In complex with.

Supplemental material Figure 13: Gene network analysis for HPV positive OP from cBioPortal tool. Network legend: Blue line: Controls state change of; Green line: Controls expression of; Brown line: In complex with.

Supplemental material Figure 14: Gene network analysis for WT HP from cBioPortal tool. Network legend: Blue line: Controls state change of; Green line: Controls expression of; Brown line: In complex with.

Supplemental material Figure 15: Gene network analysis for MUT HP from cBioPortal tool. Network legend: Blue line: Controls state change of; Green line: Controls expression of; Brown line: In complex with.
