# Supplementary material for: Computational analysis of TP53 mutational landscape unveils key prognostic signatures and distinct pathobiological pathways in head and neck squamous cell cancer
Source: Br J Cancer. 2020 Jul 20;123(8):1302–14. doi: 10.1038/s41416-020-0984-6 (PMC7553957; doi:10.1038/s41416-020-0984-6)
Supplement: Supplementary file 2 — Suppl. Figures [file 41416_2020_984_MOESM2_ESM.pdf]

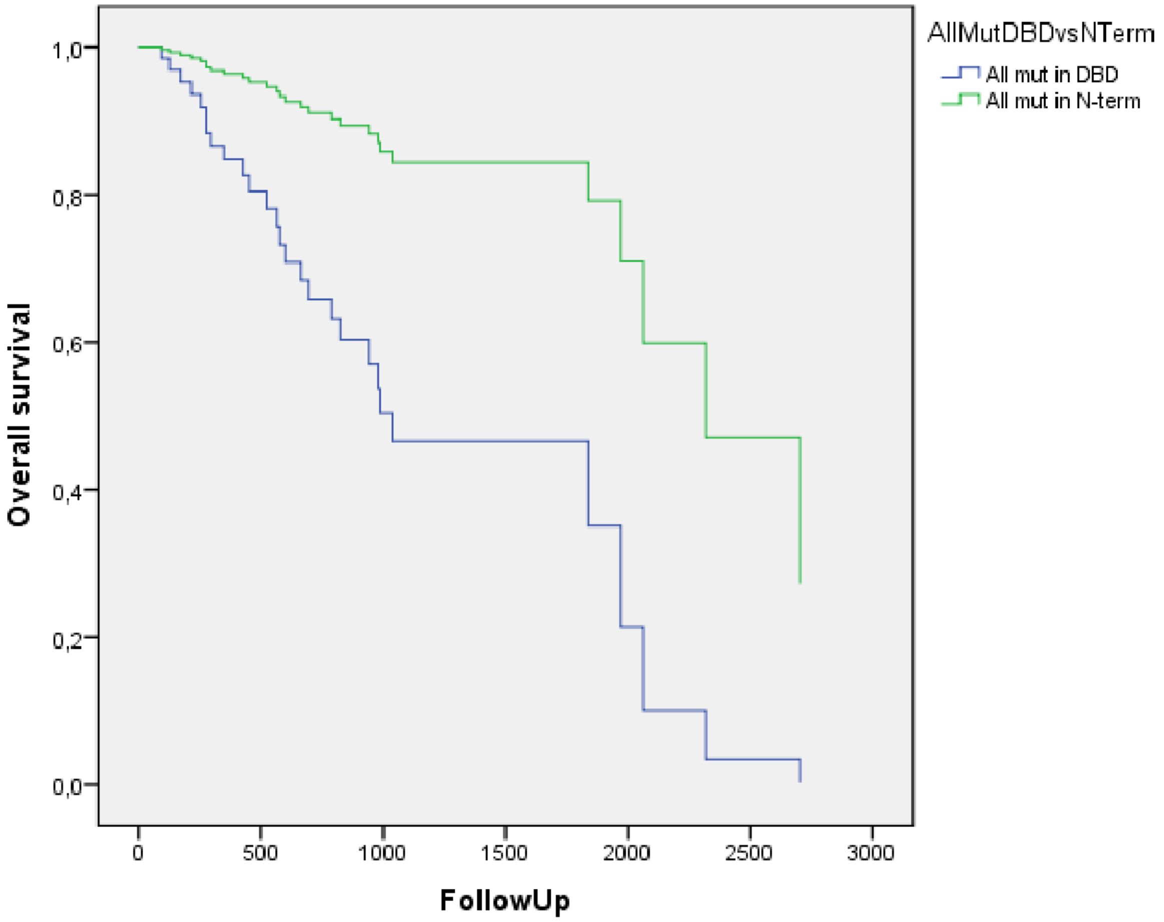

Clinic-pathological covariates	Sig.	Hazard Ratio	95% C.I.	
			Lower limit	Upper limit
Age	0,807	0,993	0,941	1,048
Stage	0,622	1,155	0,652	2,046
Grading	0,741	0,868	0,375	2,007
Gender	0,001	4,035	1,756	9,272
DBD vs N-Term	0,050	0,223	0,050	0,998

Supplemental material Figure 1: Multivariate Overall survival in larynx for TP53 mutations in DBD versus N-term.

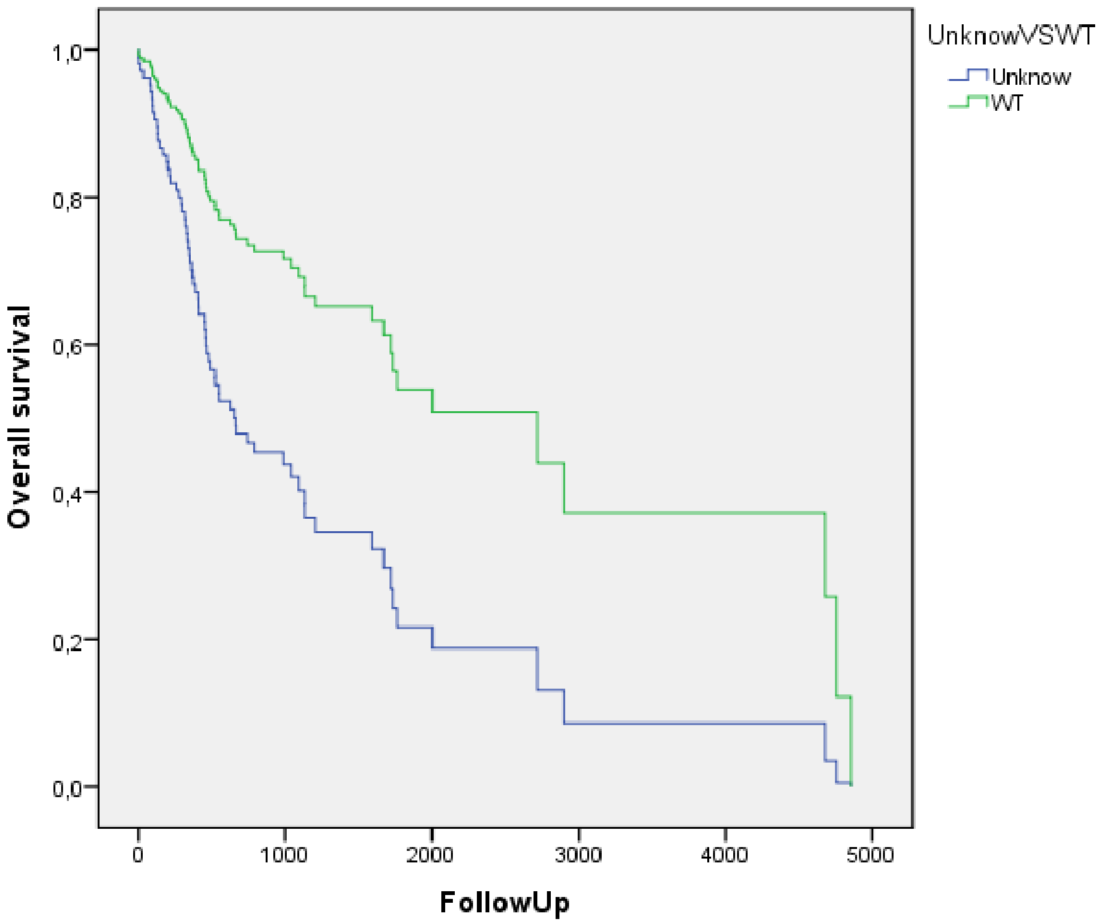

Clinic-pathological covariates	Sig.	Hazard Ratio	95% C.I.	
			Lower limit	Upper limit
Age	0,086	1,021	0,997	1,046
Stage	0,659	1,061	0,815	1,383
Grading	0,563	1,120	0,764	1,641
Gender	0,500	0,832	0,487	1,421
Unknown vs WT	0,000	2,476	1,525	4,019

Supplemental material Figure 2: Multivariate Overall survival in HNSCC for TP53 mutations in unknown secondary structure versus wild-type.

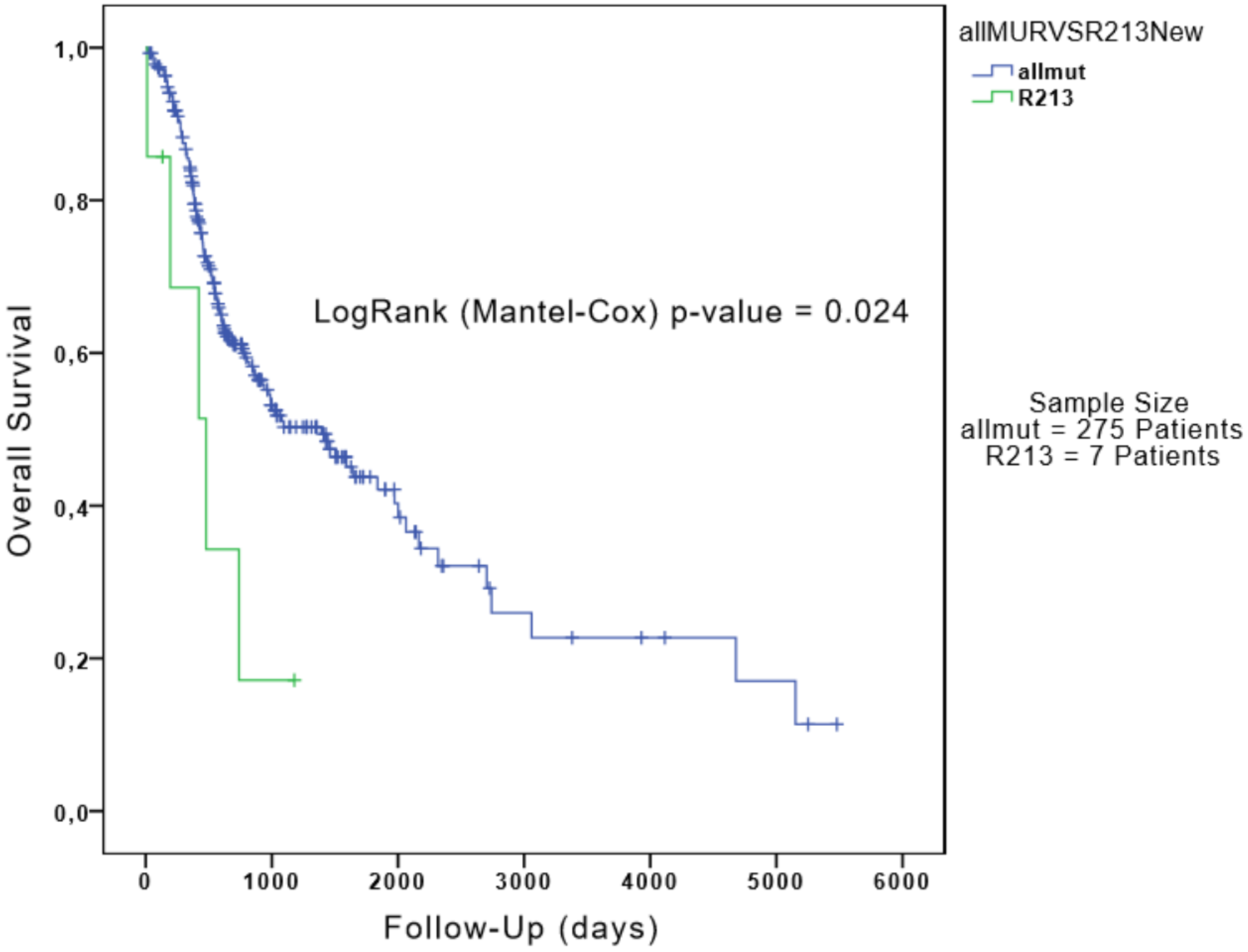

Supplemental material Figure 3: Univariate analysis for overall survival according to R213 MUT.

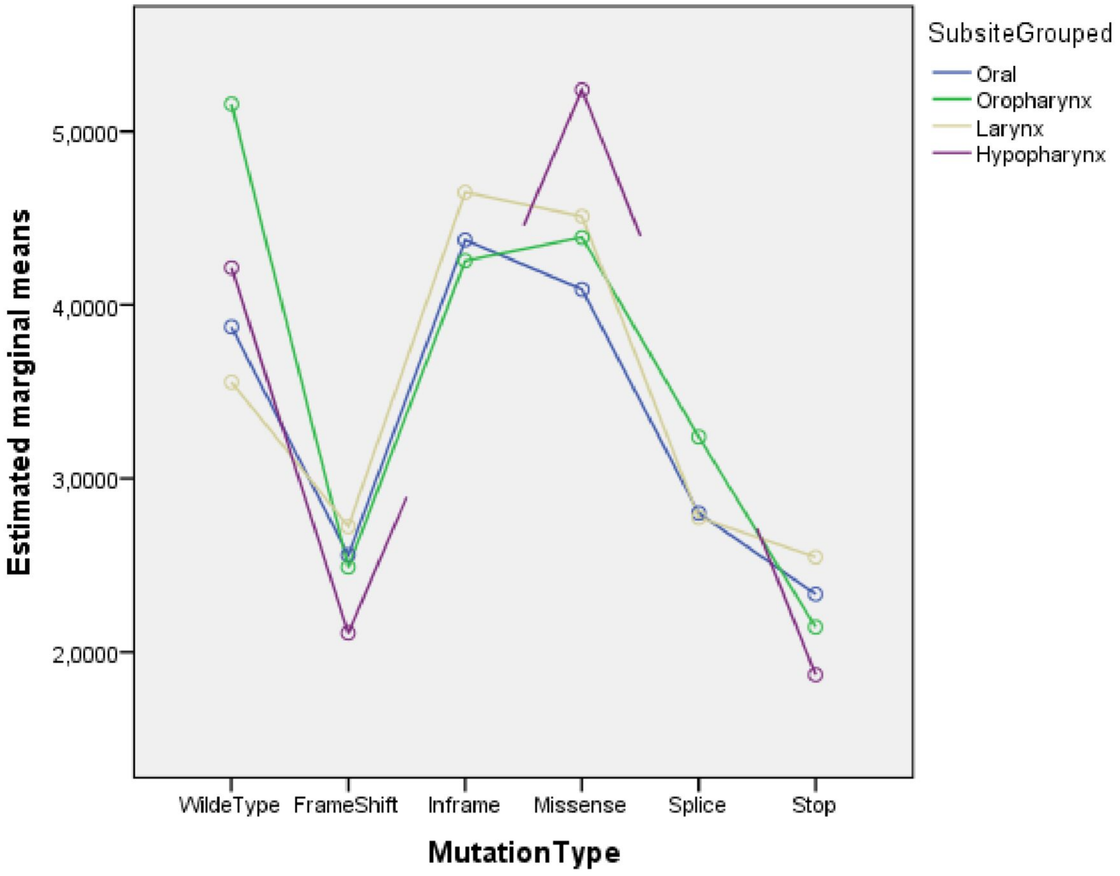

Supplemental material Figure 4: Two-way ANOVA showing the differential mRNA expression according to the mutational TP53 status and anatomical subsite.

Variable	TP53 mRNA expression	TP53 Z-score protein expression	DNA-VAF	N° of packs of cigarettes	Age	T-Dimensional	Stage	Grading
**TP53 mRNA expression**	ρ = 1	**0,382**	-0,056	-0,041	-0,038	**-0,120**	0,046	**0,203**
	p-value = 1	**<0,001**	0,362	0,449	0,446	**0,017**	0,362	**<0,001**
**TP53 Z-score protein expression**		ρ = 1	0,078	0,074	0,103	0,020	0,17	0,00
		p-value = 1	0,394	0,403	0,177	0,796	0,826	0,998
**DNA-VAF**			ρ = 1	**0,197**	0,002	0,047	0,021	**0,131**
			p-value = 1	**0,004**	0,980	0,458	0,738	**0,036**
**N° of packs of cigarettes**				ρ = 1	**0,125**	0,059	0,070	-0,008
				p-value = 1	**0,022**	0,293	0,206	0,893
**Age**					ρ = 1	-0,022	-0,062	0,059
					p-value = 1	0,671	0,219	0,244
**T-Dimensional**						ρ = 1	**0,104**	0,083
						p-value = 1	**0,041**	0,109
**Stage**							ρ = 1	0,080
							p-value = 1	0,117
**Grading**								ρ = 1
								p-value = 1

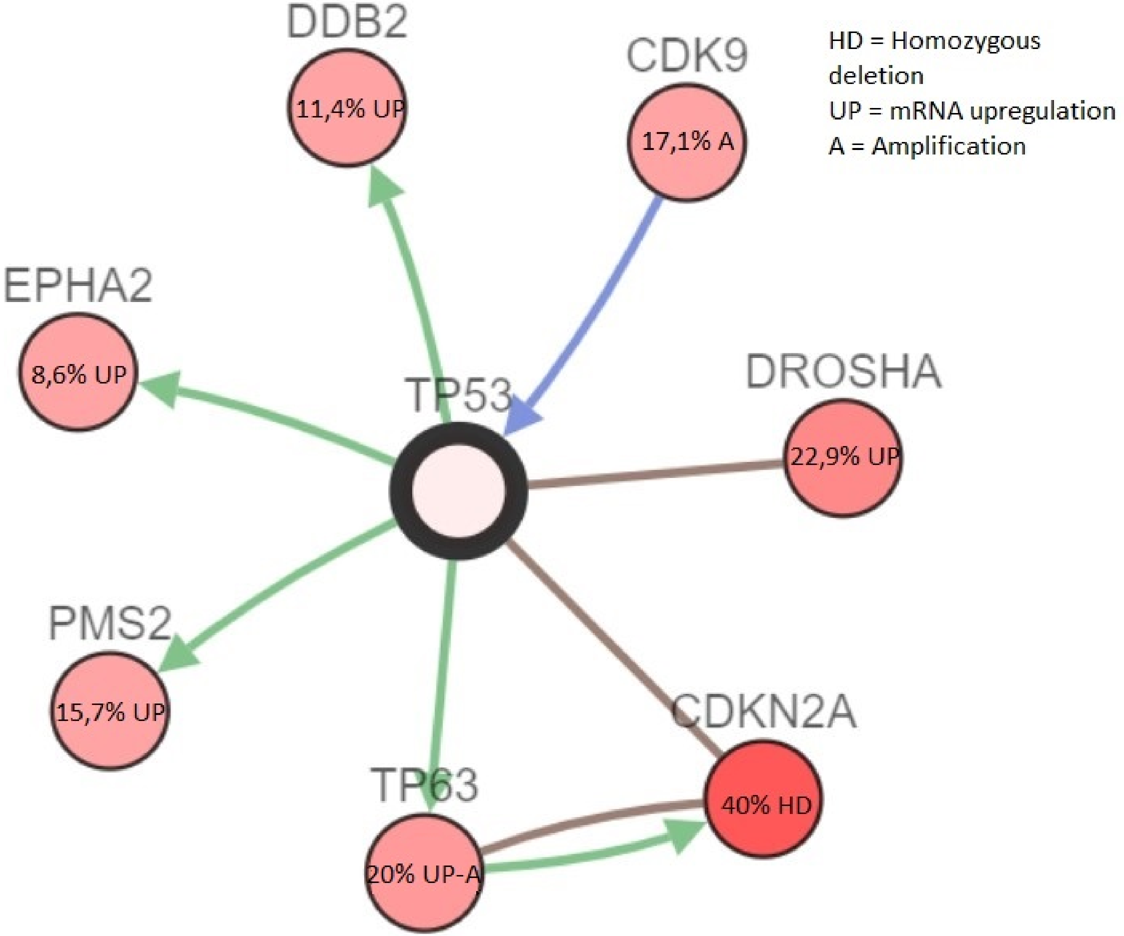

DDB2
11,4% UP
CDK9
17,1% A
HD = Homozygous deletion
UP = mRNA upregulation
A = Amplification
EPHA2
8,6% UP
TP53
DROSHA
22,9% UP
PMS2
15,7% UP
CDKN2A
TP63
40% HD
20% UP-A

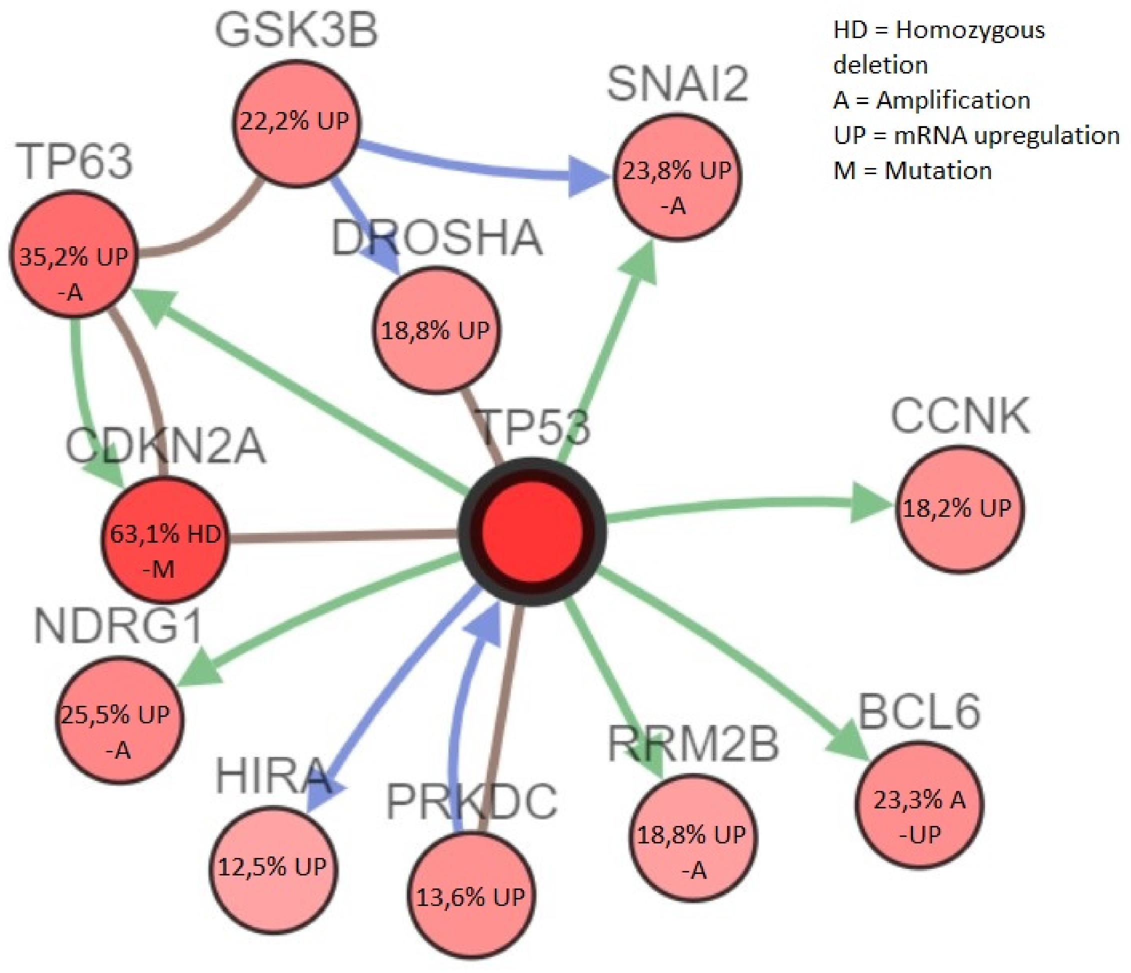
HD = Homozygous deletion
A = Amplification
UP = mRNA upregulation
M = Mutation
GSK3B
22,2% UP
SNAI2
23,8% UP -A
TP63
35,2% UP -A
DROSHA
18,8% UP
TP53
CCNK
18,2% UP
CDKN2A
63,1% HD -M
NDRG1
25,5% UP -A
HIRA
12,5% UP
PRKDC
13,6% UP
RRM2B
18,8% UP -A
BCL6
23,3% A -UP

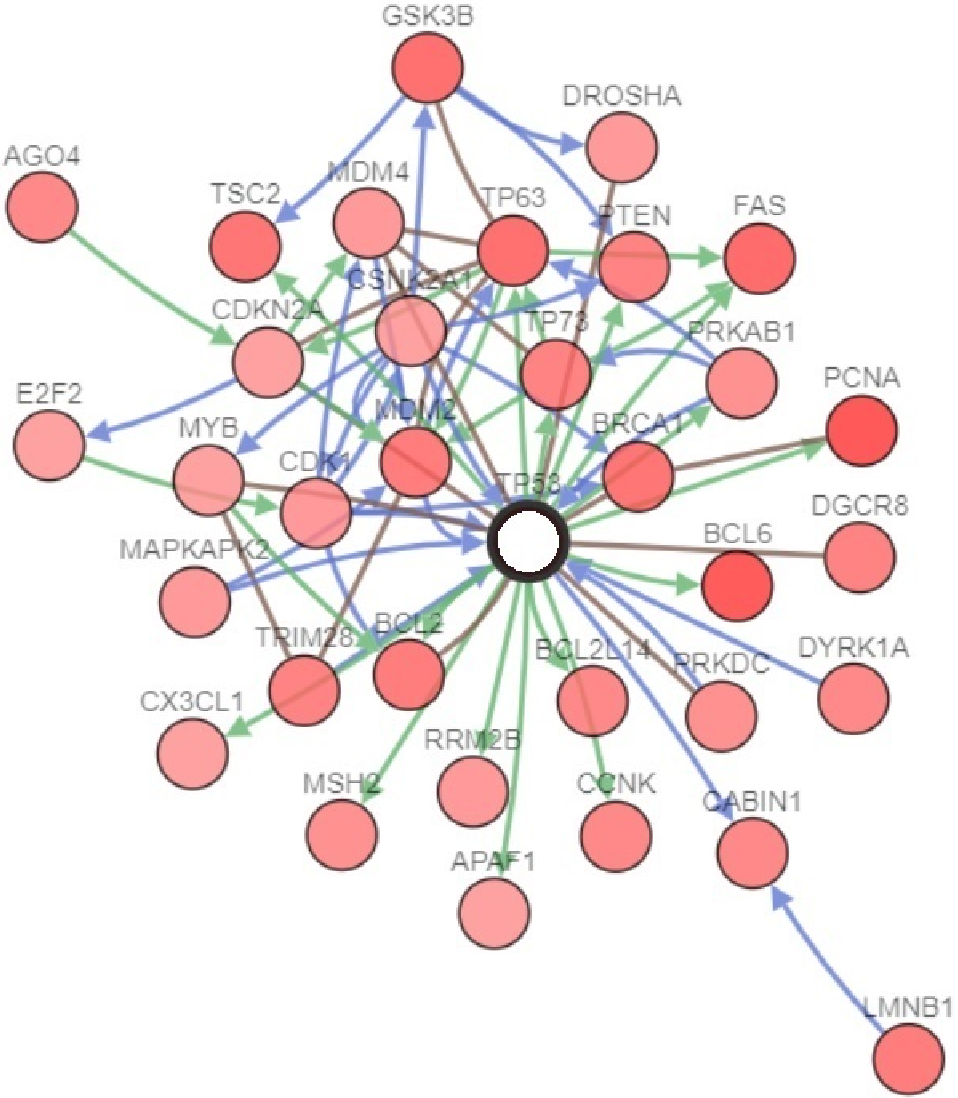

GSK3B
DROSHA
AGO4
TSC2
MDM4
TP63
PTEN
FAS
CSNK2A1
CDKN2A
TP73
PRKAB1
PCNA
E2F2
MYB
MDM2
BRCA1
CDK1
TP53
DGCR8
BCL6
MAPKAPK2
TRIM28
BCL2
BCL2L14
PRKDC
DYRK1A
CX3CL1
RRM2B
MSH2
CCNK
CABIN1
APAF1
LMNB1

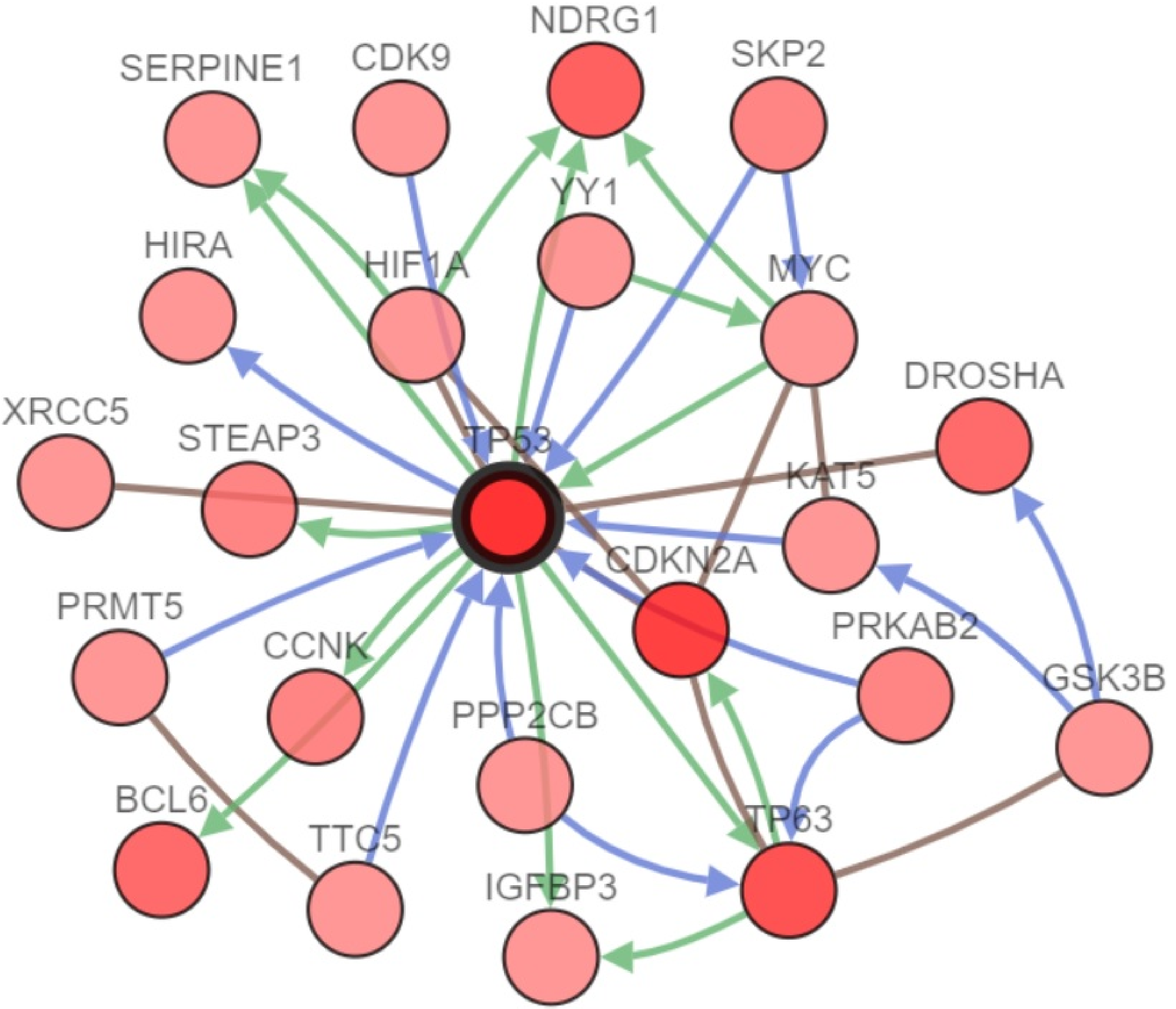
NDRG1
SERPINE1
CDK9
SKP2
YY1
HIRA
HIF1A
MYC
DROSHA
XRCC5
STEAP3
TP53
KAT5
CDKN2A
PRMT5
CCNK
PRKAB2
GSK3B
PPP2CB
BCL6
TTC5
TP63
IGFBP3

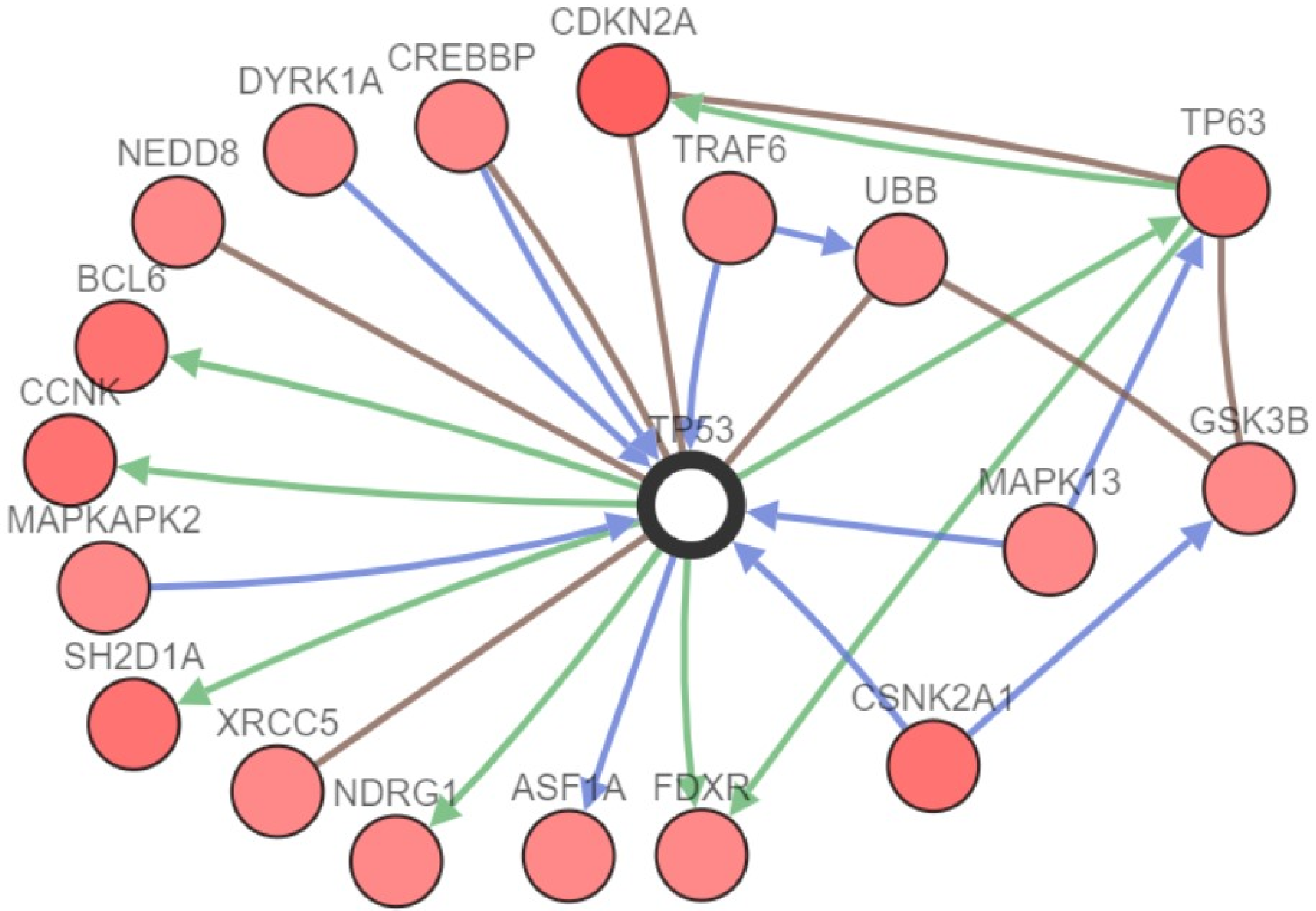
CDKN2A
CREBBP
DYRK1A
NEDD8
TRAF6
TP63
UBB
BCL6
CCNK
TP53
GSK3B
MAPK13
MAPKAPK2
SH2D1A
CSNK2A1
XRCC5
NDRG1
ASF1A
FDXR

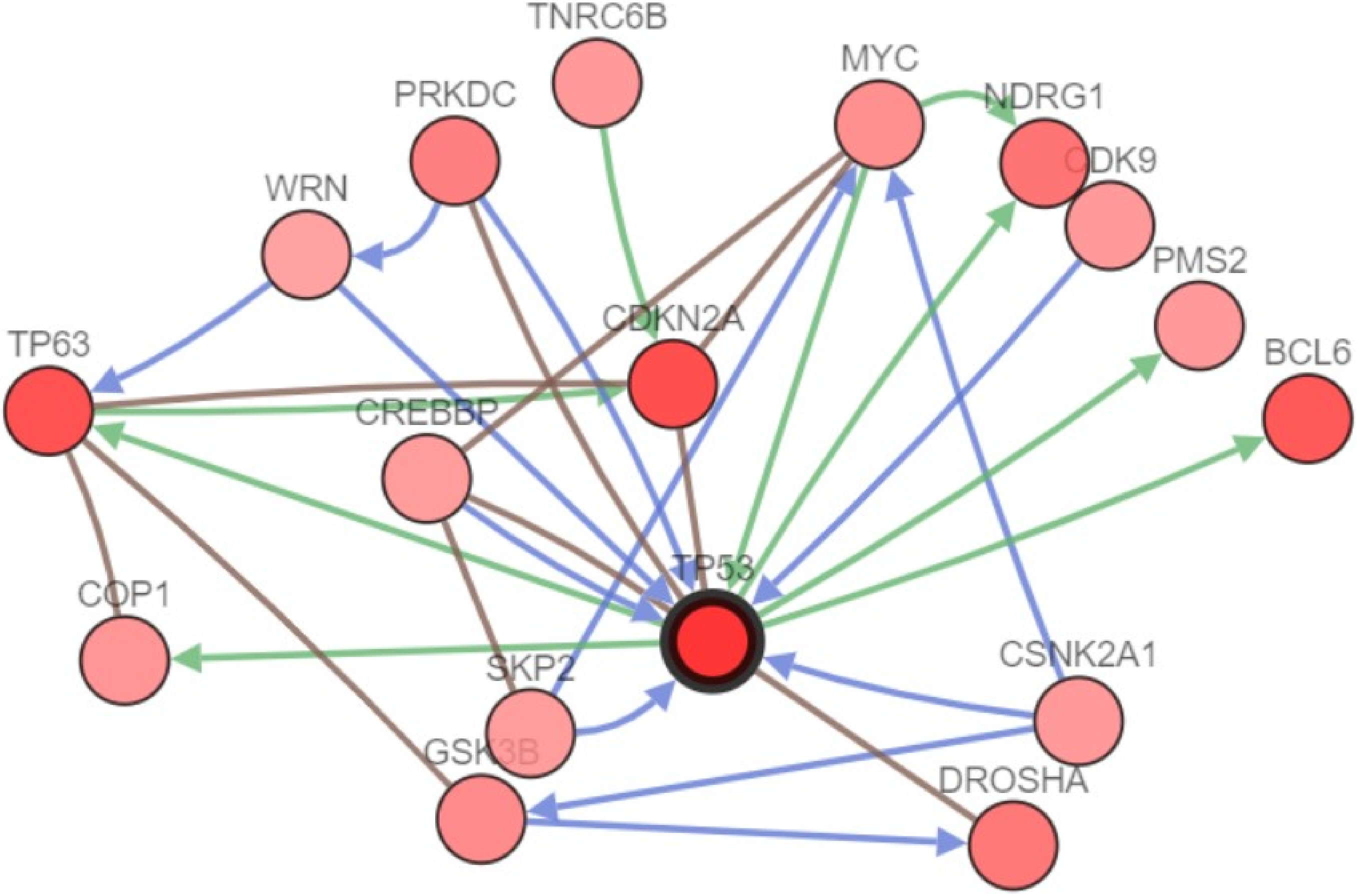

TNRC6B
MYC
PRKDC
NDRG1
CDK9
WRN
PMS2
CDKN2A
TP63
BCL6
CREBBP
TP53
COP1
CSNK2A1
SKP2
GSK3B
DROSHA

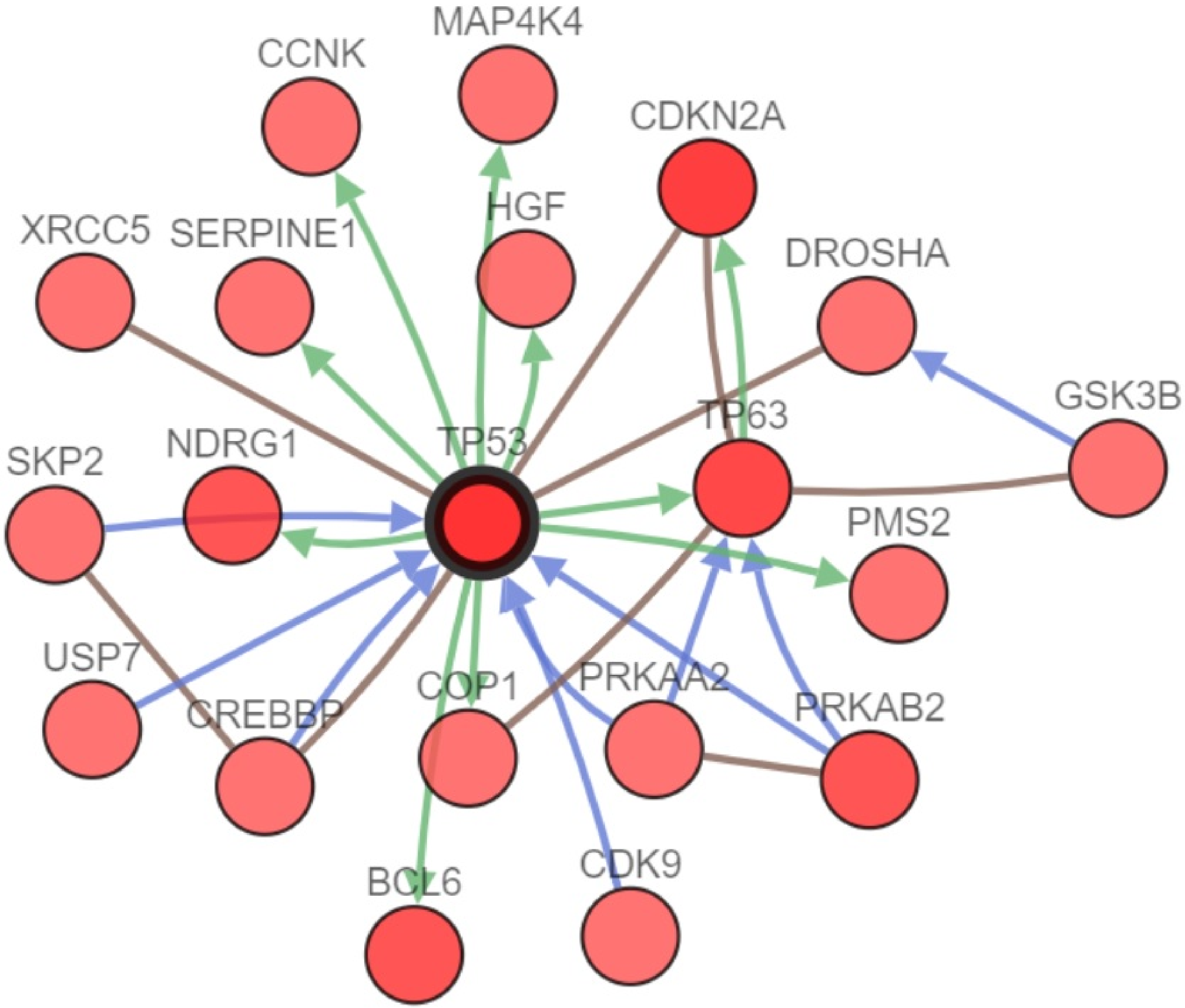
MAP4K4
CCNK
CDKN2A
HGF
XRCC5
SERPINE1
DROSHA
GSK3B
TP63
TP53
NDRG1
SKP2
PMS2
USP7
COP1
PRKAA2
CREBBP
PRKAB2
BCL6
CDK9

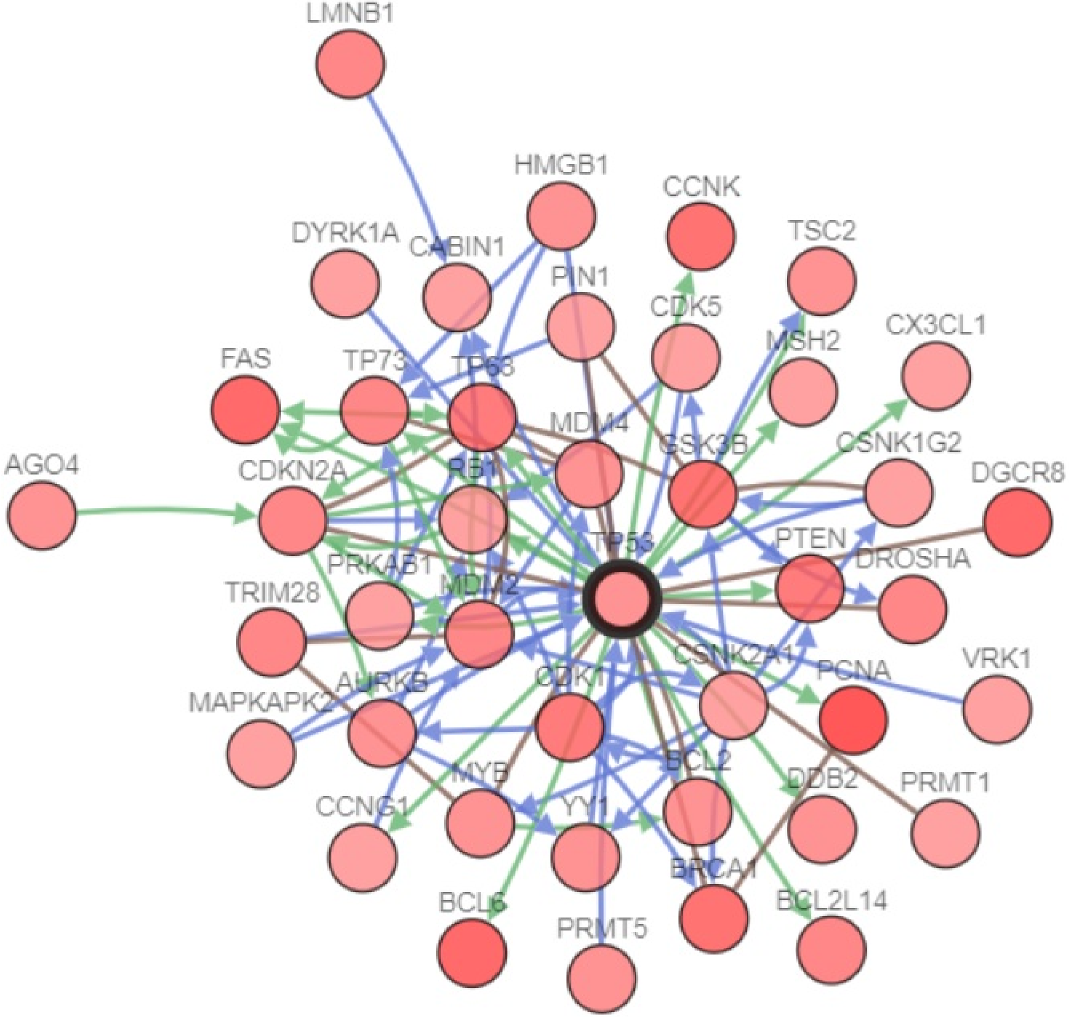
LMNB1
HMGB1
CCNK
DYRK1A
CABIN1
TSC2
PIN1
CDK5
CX3CL1
MSH2
FAS
TP73
TP63
MDM4
CSNK1G2
GSK3B
DGCR8
AGO4
CDKN2A
RB1
TP53
PTEN
DROSHA
PRKAB1
TRIM28
MDM2
CSNK2A1
VRK1
PCNA
AURKB
CDK1
MAPKAPK2
BCL2
MYB
DDB2
PRMT1
CCNG1
YY1
BRCA1
BCL2L14
BCL6
PRMT5

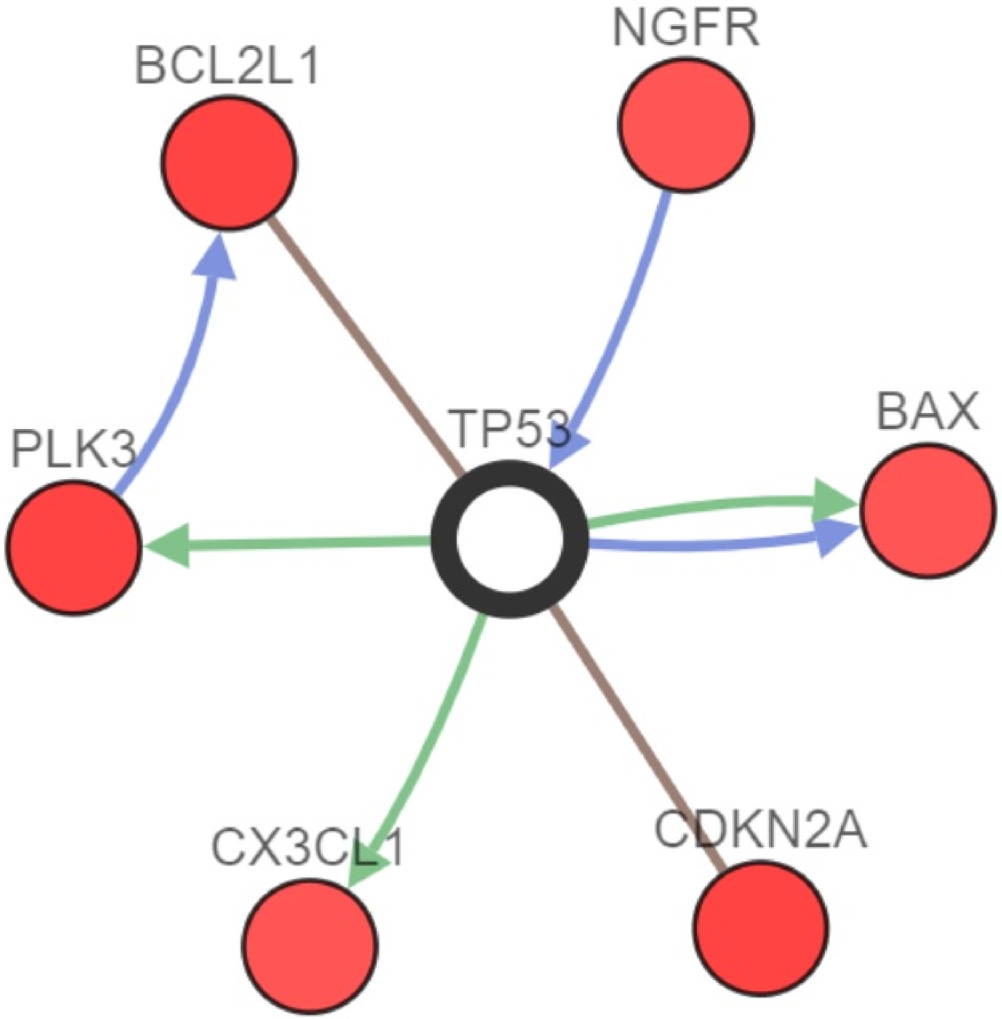
NGFR
BCL2L1
TP53
BAX
PLK3
CX3CL1
CDKN2A

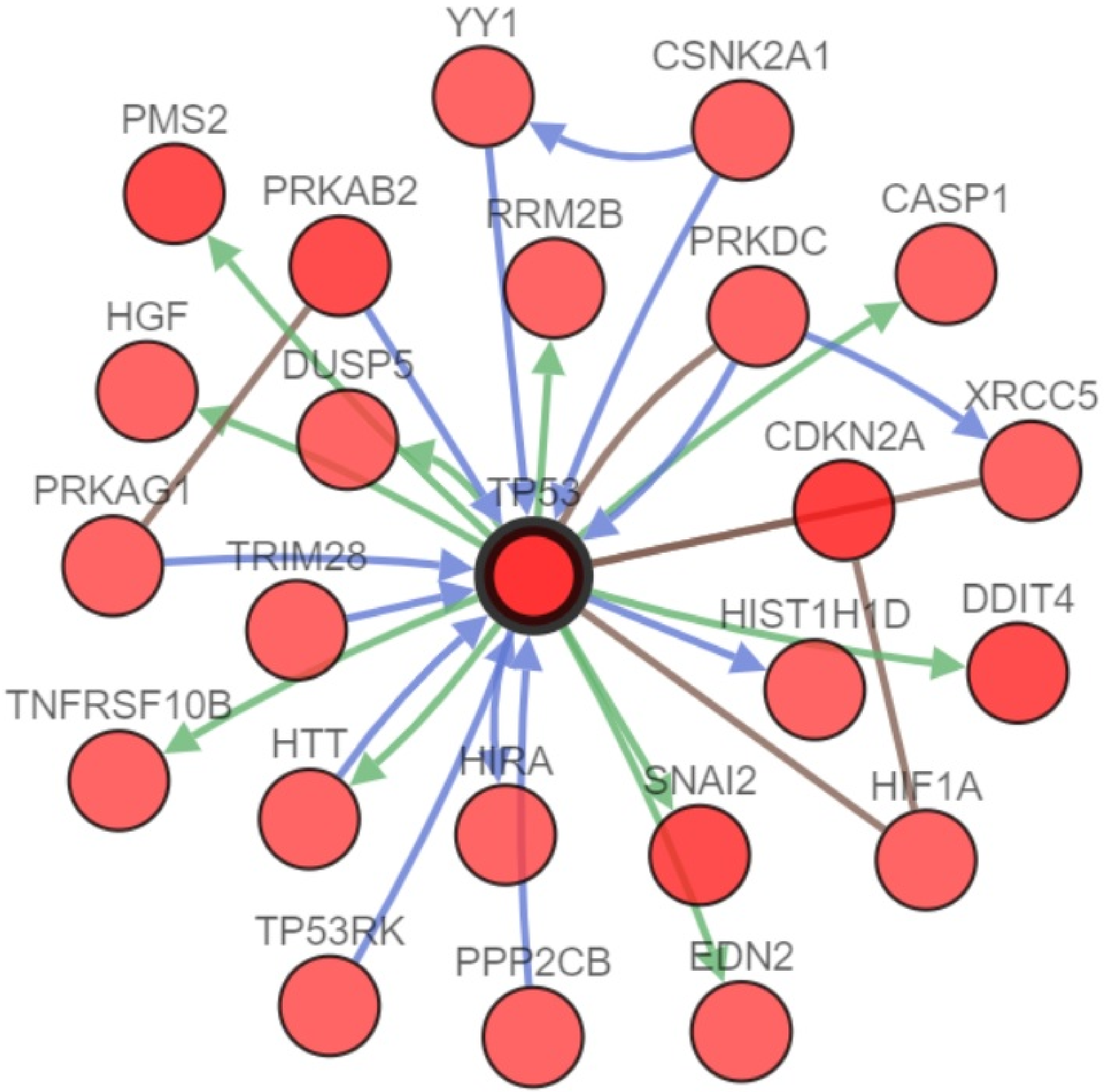

YY1
CSNK2A1
PMS2
PRKAB2
RRM2B
CASP1
PRKDC
HGF
DUSP5
XRCC5
CDKN2A
PRKAG1
TP53
TRIM28
DDIT4
HIST1H1D
TNFRSF10B
HTT
HIRA
SNAI2
HIF1A
TP53RK
PPP2CB
EDN2
